# Chinese herbal compound for multidrug-resistant or extensively drug-resistant bacterial pneumonia: a meta-analysis and trial sequential analysis with association rule mining to identify core herb combinations

**DOI:** 10.3389/fphar.2023.1282538

**Published:** 2023-12-20

**Authors:** Shuman Zhao, Yanting Geng, Jiaheng Shi, Jing Qian, Yebeilei Yang, Dan Dai, Zimin Yan, Wensheng Qi, Daxing Yu, Xin Zhao

**Affiliations:** ^1^ Guang'Anmen Hospital, China Academy of Chinese Medical Sciences, Beijing, China; ^2^ Department of Emergency, Guang'anmen Hospital, China Academy of Chinese Medical Sciences, Beijing, China; ^3^ Department of Dermatology, Guang'anmen Hospital, China Academy of Chinese Medical Sciences, Beijing, China

**Keywords:** Chinese herbal medicine, antimicrobial drug resistance, bacterial pneumonia, respiratory tract infection, meta-analysis, systematic review

## Abstract

**Purpose:** Antibiotic-resistant bacterial pneumonia poses a significant therapeutic challenge. In China, Chinese herbal compound (CHC) is commonly used to treat bacterial pneumonia. We aimed to evaluate the efficacy and safety of CHC and identify core herb combinations for the treatment of multidrug-resistant or extensively drug-resistant bacterial pneumonia.

**Methods:** Stata 16 and TSA 0.9.5.10 beta software were used for meta-analysis and trial sequential analysis (TSA), respectively. Exploring the sources of heterogeneity through meta-regression and subgroup analysis.

**Results:** Thirty-eight studies involving 2890 patients were included in the analyses. Meta-analysis indicated that CHC combined with antibiotics improved the response rate (RR = 1.24; 95% CI: 1.19–1.28; *p* < 0.0001) and microbiological eradication (RR = 1.41; 95% CI: 1.27–1.57; *p* < 0.0001), lowered the white blood cell count (MD = −2.09; 95% CI: −2.65 to −1.53; p < 0.0001), procalcitonin levels (MD = −0.49; 95% CI: −0.59 to −0.40; p < 0.0001), C-reactive protein levels (MD = −11.80; 95% CI: −15.22 to −8.39; *p* < 0.0001), Clinical Pulmonary Infection Scores (CPIS) (MD = −1.97; 95% CI: −2.68 to −1.26; *p* < 0.0001), and Acute Physiology and Chronic Health Evaluation (APACHE)-II score (MD = −4.08; 95% CI: −5.16 to −3.00; p < 0.0001), shortened the length of hospitalization (MD = −4.79; 95% CI: −6.18 to −3.40; *p* < 0.0001), and reduced the number of adverse events. TSA indicated that the response rate and microbiological eradication results were robust. Moreover, *Scutellaria baicalensis* Georgi*, Fritillaria thunbergii* Miq*, Lonicera japonica* Thunb*,* and *Glycyrrhiza uralensis* Fisch were identified as core CHC prescription herbs.

**Conclusion:** Compared with antibiotic treatment, CHC + antibiotic treatment was superior in improving response rate, microbiological eradication, inflammatory response, CPIS, and APACHE-II score and shortening the length of hospitalization. Association rule analysis identified four core herbs as promising candidates for treating antibiotic-resistant bacterial pneumonia. However, large-scale clinical studies are still required.

**Systematic Review Registration:** https://www.crd.york.ac.uk/prospero/, identifier CRD42023410587.

## 1 Introduction

Lower respiratory tract infections are the fourth leading cause of mortality worldwide, claiming 2.6 million lives in 2019 (World Health Organization, 2020). Although several organisms are implicated, bacteria remains the primary cause of pneumonia. The introduction of antibiotics considerably improved bacterial pneumonia treatment efficiency. However, antibiotic overuse and misuse in hospitals and the agricultural industry have contributed to the emergence of drug resistance (Chawla et al., 2022). In recent years (Zaman et al., 2017; Duval Raphaël et al., 2019; Salam et al., 2023), multidrug-resistant (MDR) bacteria, defined as bacteria with resistance to at least three different classes of antimicrobial agents, have become common, promoting selection pressure toward extensively drug-resistant(XDR) bacteria. One study reported that 28%–75% of patients with hospital-acquired pneumonia (HAP) carried MDR bacteria (Catia et al., 2019). These bacteria, most often *Pseudomonas aeruginosa* (*P. aeruginosa*)and methicillin-resistant *Staphylococcus aureus* (MRSA), are the main causes of early-onset and late-onset ventilator-associated pneumonia (VAP) (Torres et al., 2017). In addition to nosocomial pneumonia, recent studies have reported an increased risk of MDR and XDR bacteria spreading to the community (Van Duin and Paterson, 2020; NAIR and Niederman, 2021). In one study, the “PES” pathogens, including *P. aeruginosa*, extended-spectrum β-lactamase-positive *Enterobacteriaceae*, and MRSA, were responsible for 6% of hospitalized community-acquired pneumonia (CAP) cases and resulted in high mortality (Prina et al., 2015). Macaux et al. (Lou et al., 2018) reported that 35% of XDR bacteria carriers had not been hospitalized in the past 12 months. As the emergence of MDR and XDR bacteria reduces antibiotic efficacy, lower respiratory tract infections can not be controlled, leading to longer hospitalization durations, higher costs, and increased mortality (Venter, 2019; Yahav et al., 2021; Raycheva et al., 2022). The shortage of new antibiotics developed against MDR and XDR bacteria aggravates this problem (Terreni et al., 2021).

Currently, researchers have shifted their focus to developing new antibiotics from botanicals. Complex natural products with numerous molecular targets can reduce the incidence of resistance and thus exhibit considerable antibacterial activity against MDR bacteria (Filipa et al., 2020; Hafsa et al., 2022; Thangaiyan et al., 2022). Traditional Chinese medicine(TCM) is characterized by the use of botanicals, a rich source of bioactive phytocompounds. Many bioactive phytocompounds originating from TCM herbs, such as phenols, terpenoids, alkaloids, flavonoids, isothiocyanates, and indoles, hinder major drug-resistant factors, such as efflux pumps, enzyme activity, membrane permeability, and other virulence mechanisms, including quorum sensing (QS) and biofilm development (Gupta and Birdi, 2017; Su et al., 2020; Li et al., 2022). Chinese herbal compound (CHC) represents a prevalent therapeutic approach in TCM. Several randomized control trials (RCTs) have demonstrated the efficacy of CHC alone or in combination with antibiotics in treating MDR and XDR bacterial pneumonia, improving the eradication rate of antibiotic-resistant bacteria, reducing the inflammatory response and adverse effects, and shortening the duration of hospitalization. However, the therapeutic efficacy and safety of CHC or CHC combination therapy remains indeterminate due to the lack of comprehensive evaluation of these RCTs. Therefore, this study aimed to produce a novel meta-analysis and trial sequential analysis (TSA) to reliably estimate the clinical effects and safety of CHC for treating MDR and XDR bacterial pneumonia. Association rule mining using the Apriori algorithm was performed to identify core herbal combinations that could serve as potential therapeutic agents.

## 2 Methods

This study was conducted according to the Preferred Reporting Items for Systematic Review and Meta-Analysis Protocols (PRISMA) 2020 guidelines (Supplementary Material S1). The registration number of the study protocol is CRD42023410587 (PROSPERO: https://www.crd.york.ac.uk/prospero/).

### 2.1 Literature search strategy

RCTs that evaluated the efficacy and safety of CHC for MDR or XDR bacterial pneumonia were obtained from eight databases, including Pubmed, Embase, Cochrane Library, Web of Science (WOS), China National Knowledge Infrastructure (CNKI), Wanfang Data Knowledge Service Platform (Wanfang), VIP information resource integration service platform (VIP), and Chinese Biomedical Literature Database (CBM), and independently screened by two investigators (JS. and ZY.). The coverage dates ranged from the inception of each database (Pubmed, 1946; Embase, 1947; The Cochrane Library, 1995; WOS, 1900; CNKI, 1994; Wanfang, 1980; VIP, 1989; CBM, 2008) until 10 March 2023. Medical subject heading (MeSH) and free terms were combined for retrieval in this study, and the search terms were appropriately modified for the different databases. Keywords used for the literature search included “Drug-resistant bacterial pneumonia,” “Antibiotic-resistant bacterial pneumonia,” “Multidrug-resistant bacterial pneumonia,” “extensively drug-resistant bacterial pneumonia,” “Medicine, Chinese Traditional,” “Traditional Chinese Medicine,” “decoction,” “formula,” “randomized controlled trial,” and “RCT.” Complete retrieval methods are detailed in Supplementary Material S2.

### 2.2 Study selection

EndNote reference management software (version 20; Clarivate Analytics) was used for study selection. Two reviewers (DD. and YY.) separately screened the titles, abstracts, and full text for eligibility using the same criteria. Disagreements were resolved by a third reviewer (DY.).

#### 2.2.1 Eligibility criteria

##### 2.2.1.1 Type of studies

All RCTs, with or without blinding, were included in this study. No specified date or language restrictions were set.

##### 2.2.1.2 Type of participants

Patients with any type of pneumonia (VAP, HAP, or CAP) caused by MDR or XDR bacteria were included without restrictions for sex, age, or race. Positive cultures from endotracheal aspirates or bronchoscopic sampling techniques were necessary for the diagnosis of antibiotic-resistant bacterial pneumonia. MDR bacteria were defined as bacteria unsusceptible to at least three different antimicrobial categories, and XDR bacteria were defined as bacteria unsusceptible to at least one agent in addition to two or fewer antimicrobial classes (Magiorakos et al., 2012). The pathogenetic diagnosis was based on at least two etiological examinations of cultures from lower respiratory tract secretions, including sputum, endotracheal aspirate, or bronchoalveolar lavage fluid. Furthermore, abnormal radiological findings indicating the presence of new or progressed pulmonary infiltrate(s) and clinical signs of infection, including the onset of fever (≥38°C), increased sputum production or change in sputum color to a more purulent state, peripheral leukocytosis, and decreased oxygenation or the requirement of oxygen supplementation therapy, were required (Kalil et al.; Olson and Davis, 2020).

##### 2.2.1.3 Type of intervention

RCTs comparing CHC in the treatment group with antibiotic treatment in the control group, or comparing combined CHC + antibiotic treatment with antibiotic-only treatment, with or without a placebo, were included. All forms of oral administration or nasal feeding, such as through decoction, granules, or capsules, were included. No restrictions were placed on the dose or duration of CHC therapy.

##### 2.2.1.4 Outcome measures

The primary outcome was set as response rate, calculated as (total number of patients—number of patients without efficacy)/total number of patients. Inefficacy was defined as unaltered or worsening signs and symptoms, such as pulmonary shadows visualized on X-ray or CT, pulmonary rales, fever, cough, and chest pain.

Secondary outcomes included: 1) microbiological response, defined as bacterial eradication determined by etiological examination of clinical samples collected from the site of infection showing no bacterial growth at the end of treatment; 2) inflammatory indicators, including absolute white blood cell (WBC) count, procalcitonin (PCT) levels, and C-reactive protein (CRP) levels; 3) scoring systems that assessed the severity of pneumonia, prognosis, and drug efficacy, including the Clinical Pulmonary Infection Scores (CPIS) and Acute Physiology and Chronic Health Evaluation (APACHE)-II score; 4) safety, evaluated by the incidence of adverse events (AEs) and adverse drug reactions (ADRs).

#### 2.2.2 Exclusion criteria

Exclusion criteria included: 1) cohort studies, case reports, reviews, and conference abstracts; 2) studies that lacked primary outcomes, control groups, or full-text articles; 3) participants diagnosed without an etiological examination or a drug sensitivity test, or those diagnosed with an etiological examination indicating *chlamydia*, *mycoplasma*, or fungal infection; 4) interventions involving non-drug TCM therapy, such as acupuncture or massage.

### 2.3 Data extraction and quality assessment

A standardized data extraction form including the title, first author’s name, publication year, age, sample size, sex, interventions in the treatment and control groups, treatment duration, infection type, pathogen, setting, outcome measures, and reported AEs or ADRs was used. Five methodological quality domains were assessed using Rob 2.0 and included: 1) bias arising from the randomization process; 2) bias due to deviations from intended interventions; 3) bias due to missing outcome data; 4) bias in outcome measurements; 5) bias in the selection of the reported result. Each domain was categorized according to three levels, namely, “high risk of bias,” “low risk of bias,” or “some concerns”. Two reviewers (Y.G. and S.Z.) independently evaluated the methodological quality of each study, and disagreements were resolved by a third reviewer (JQ.).

### 2.4 Statistical analysis

Meta-analysis was performed using Stata 16.0. Binary outcomes were expressed in terms of relative risk (RR) and 95% confidence interval (CI). For continuous variables, the mean difference (MD) and 95% CI were used. When I^2^ was <50%, the fixed-model Mantel–Haenszel method was used to evaluate the pooled effect. When I^2^ was >50%, a random-effects model was used. A sensitivity analysis was performed to evaluate the stability of the research findings. To explore potential sources of heterogeneity, meta-regression and subgroup analyses were performed. Meta-regression was used when the number of included studies was greater than 10. Begg’s and Egger’s tests were used to assess publication bias, and trim-and-fill analyses were performed to confirm the results. To avoid type I and type II errors from the meta-analysis and to obtain more robust results (Pogue and Yusuf, 1998; Shah and Smith, 2019), TSA was conducted using TSA 0.9.5.10 beta software. The quality of evidence for all outcomes was evaluated using the GRADE system.

To investigate likely association rules of core herb combinations, we examined CHC constituent herb data. Preliminary information for data extraction was obtained by analyzing the frequency of each herb prior to association rule analysis. Apriori association rule analysis and plot generation were performed using the “arules” R-package (version 2023.03.0 + 386) to fit data and “arulesViz” to generate the plots (Merdun and Ozturk, 2005; Hahsler, 2017). To detect meaningful connections between variables in large databases, frequent hub item sets and association rules were mined using the Apriori algorithm. This method can identify relationships between projects and can be used in various medical studies designed to predict variable characteristics (Hsieh et al., 2020).

In the Apriori algorithm, the primary metrics used to measure associations include support, confidence, and lift. Support, defined as the proportion of transactions that contain the itemset in the dataset, measures the importance of the itemset and is expressed as P (A∩B). Confidence, which measures the probability of observing herb A in a transaction containing herb B, can be expressed as P(A∩B)/P(A) = P(B|A) but does not explain whether there is a meaningful correlation between the two transactions. Lift, which can be expressed as P (A with B)/P (A) P (B) = P(B|A)/P(B), can identify meaningful correlations between two transactions. Herb A and Herb B are unrelated when lift is close to 1, as they are probabilistically close to independent, whereas lift >1 indicates a strong relationship between Herb A and Herb B. To identify association rules, minimum support and confidence values were set at 20% and 80%, respectively, and core herb combinations demonstrating the most evident associations were identified.

## 3 Results

### 3.1 Study selection

**FIGURE 1:**
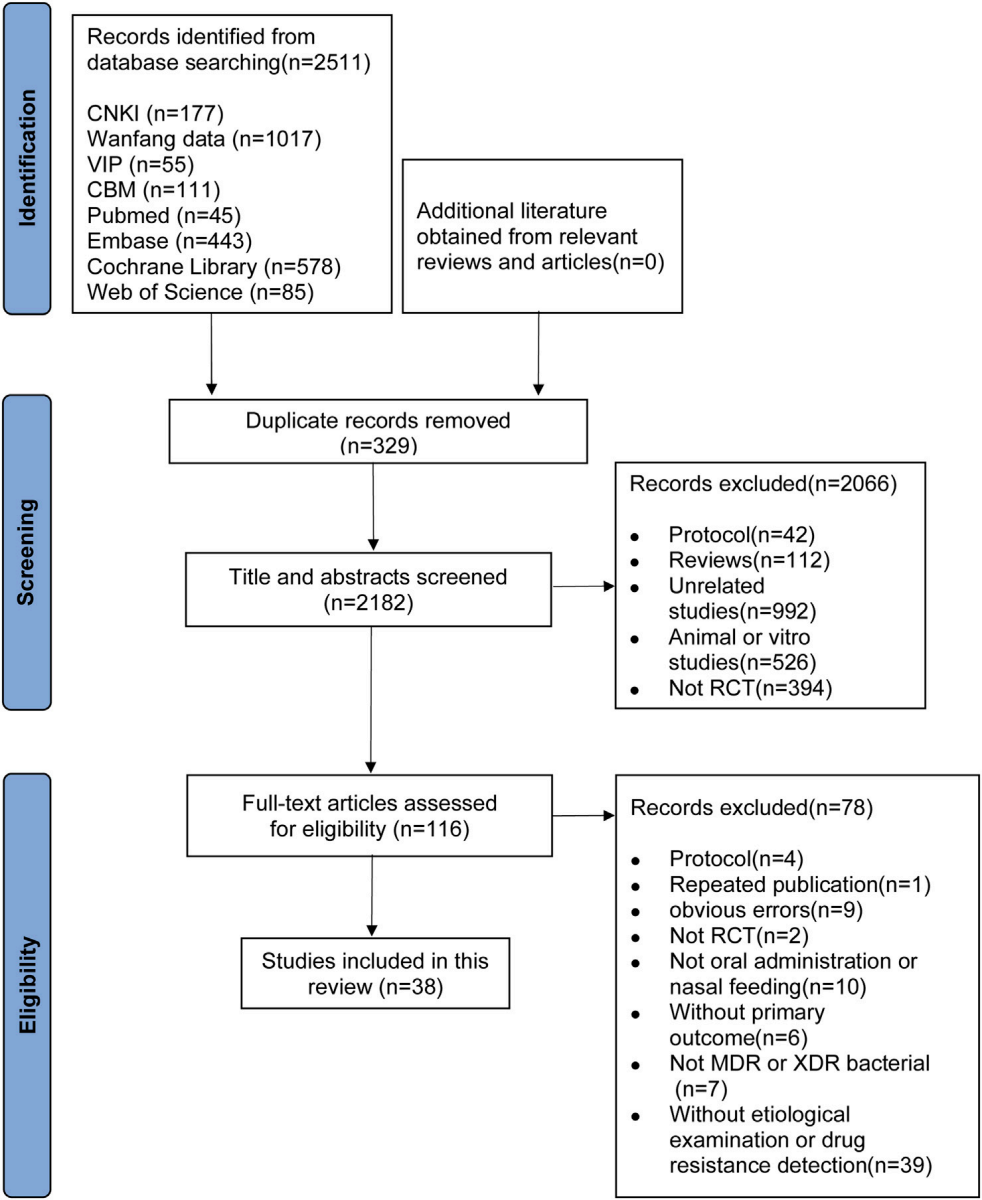
Flow chart of study selection and identification.

The initial search of the eight electronic databases yielded a total of 2511 articles. After removing duplicates, the remaining 2182 articles were screened for eligibility, and of the 116 eligible studies, 38 met the inclusion criteria (Han and Huang, 2006; Jin et al., 2015; Yang et al., 2015; Shujuan et al., 2015; Xue, 2015; Chengdu University of Traditional Chinese Medicine, 2016; Liu and Song, 2016; Liang et al., 2016; Zhu et al., 2016; Shi et al., 2017; Hu et al., 2017; Xie, 2017; Guo et al., 2017; Zhang and Liu, 2018; Xu et al., 2019; Huang et al., 2019a; Tan, 2019; Liang et al., 2019b; Liang et al., 2019a; Liu et al., 2019; Wang, 2019; Peng et al., 2019; Huang et al., 2019b; Yan et al., 2020; Beijing University of Chinese Medicine, 2020; Gao et al., 2021; Wang and Songtao, 2020; Zhong et al., 2020; Xiao et al., 2021; Clinical, 2021; Zhou and Luo, 2021; Li et al., 2022; Fu et al., 2022; Chen, 2022; Shandong University of Traditional Chinese Medicine, 2022; WANG, 2022; Yin et al., 2022) (Figure 1).

### 3.2 Study characteristics

Thirty-eight RCTs conducted in China between 2006 and 2022, involving 2890 patients, with 1431 patients in the intervention group and 1459 patients in the comparison group, were included. The median age of the patients was 59.55 years. Most of the studies included middle-aged or elderly adults, and only one study recruited toddlers. Table 1 summarizes the characteristics of the 38 included studies.

**TABLE 1:** Characteristics of the included studies.

Study ID	Age (mean ± SD)		Number of patients (male/female)		Interventions		Treatment duration	Infection type	Pathogen	Setting	Outcomes	Adverse event (cases/symptom)
	Trial	Control	Trial	Control	Trial	Control						
Han (2006)	2 ± 0.8	2∙2 ± 0.7	45 (24/21)	40 (22/18)	(1) Modified Tingli Dazao Xiefei decoction(po,bid) (2) Imipenem and Cilastatin Sodium for Injection (10–15 mg/kg,iv,bid)	Imipenem and Cilastatin Sodium for Injection (10–15 mg/kg,iv,bid)	7 days	NR	*Escherichia coli*	Department of intergrated traditional Chinese and western medicine	①②⑧	NR
Duan (2015)	52.13 ± 4.33	53.38 ± 5.14	50 (34/16)	50 (32/18)	(1) Traditional Chinese medicine decoction (100 ml,po,tid) (2) Antibiotic regimens based on drug sensitivity results	Antibiotic regimens based on drug sensitivity results	14 days	NR	*Klebsiella pneumoniae*, *Escherichia coli*, *Pseudomonas aeruginosa*, *Acinetobacter baumannii*	ICU	①	NR
Huang (2019)	49.8 ± 2.2	49.6 ± 2.3	39 (20/19)	39 (21/18)	Xia Yuhuang decoction(po,bid)	Fosfomycin Sodium for Injection (4 g,iv,tid); Cefoperazone Sodium and Sulbactam Sodium for Injection (4 g,iv,tid)	14 days	NR	*Pseudomonas aeruginosa*	NR	①③⑧⑨	T: 1 case (1 dizziness) C: 1 cases (3 dizziness, 2 nausea and vomiting, 2 dry skin)
Huang (2019)	54.16 ± 4.52	53.79 ± 4.38	60 (39/21)	60 (38/22)	(1) Qingfei Tongluo decoction (400 ml,po,bid) (2) Antibiotic regimens based on drug sensitivity results	Antibiotic regimens based on drug sensitivity results	10 days	NR	NR	NR	①⑥	NR
Qi (2015)	61 ± 3.35	58 ± 5.7	17 (10/7)	16 (8/8)	(1) Tongfu Xiefeidecoction (200 ml,po,bid) (2) Cefoperazone Sodium and Sulbactam Sodium forInjection (3 g,iv,bid)	Cefoperazone Sodium and Sulbactam Sodium forInjection (3 g,iv,bid)	7 days	NR	*Acinetobacter baumannii*	ICU	①	NR
Xue (2015)	55.71 ± 29.47	56.22 ± 28.45	40 (32/8)	40 (33/7)	(1) Traditional Chinese medicine decoction(po,bid) (2) Tigecycline (first day 100 mg, and continue 50 mg,iv,bid)	Tigecycline (first day 100 mg, and continue 50 mg,iv,bid)	7 days	NR	*Acinetobacter baumannii*	ICU	①②③⑤⑨	T: 4 cases (2 nausea, 2 vomiting) C: 9 cases (3 nausea, 2 vomiting, 2 rash, 2 thrombocytopenia)
Yang (2015)	58.32 ± 10.5	59.46 ± 10.8	46 (24/22)	46 (25/21)	(1) Yiqi Jianpi Huatan decoction(po,bid) (2) Imipenem And Cilastatin Sodium for Injection (500 mg,iv,tid)	Imipenem And Cilastatin Sodium for Injection (500 mg,iv,tid)	14 days	NR	*Pseudomonas aeruginosa*	Department of neurology	①④	NR
Liu (2016)	56.4 ± 9.1	55.6 ± 11.2	30 (20/10)	30 (18/12)	(1) Modified Xiao Qinglong decoction (200 ml,po,bid) (2) Antibiotic regimens based on drug sensitivity results	Antibiotic regimens based on drug sensitivity results	14 days	HAP	NR	Department of neurology	①⑥	NR
Liang (2016)	73.21 ± 14.38	72.43 ± 15.14	34 (20/14)	33 (18/15)	(1) Feigan 2 decoction (200 ml,po,bid) (2) Antibiotic regimens based on drug sensitivity results	Antibiotic regimens based on drug sensitivity results	14 days	CAP/HAP/VAP	*Klebsiella pneumoniae*, *Pseudomonas aeruginosa*, *Acinetobac er baumannii*, *Staphylococcus aureus*, *Escherichia coli*, *Stenotrophomonas maltophilia*, *Burkholderia cepacia*, *Staphylococcus epidermidis*, *Enterobacter cloacae*, *Enterococcus*, *Staphylococcus haemolyticus*, *Klebsiella oxytoca*	ICU	①②④⑤⑦⑨	T: 2 cases (2 diarrhea) C: 1 case (1 diarrhea) Mild elevated alanine aminotransferase was found in both groups, and liver function returned to normal after liver protection treatment and drug withdrawal during the full treatment period
Tang (2016)	74.5 ± 11.53	72.15 ± 10.01	39 (24/15)	39 (21/18)	(1) Modified Liu Junzi decoction (200 ml,po,tid) (2) conventional treatment (Antibiotics)	conventional treatment (Antibiotics)	14 days	CAP/HAP	*Klebsiella pneumoniae*, *Pseudomonas aeruginosa*, *Acinetobacter baumannii*, *Escherichia coli*, *Staphyloccocus aureus Rosenbach*, *Enterococcus*	EICU/ER/Department Respiratory and Geriatrics	①②③④⑦⑨	T: 5 cases (2 nausea, 2 diarrhea, 1 rash) C: 3 cases (3 rash)
Zhu (2016)	60.95 ± 11.57	62.69 ± 14.43	40 (29/11)	39 (22/17)	(1) Qingfei Tongluo decoction (200 ml,po,tid) (2) Antibiotic regimens based on drug sensitivity results	Antibiotic regimens based on drug sensitivity results	14 days	NR	NR	Department of rehabilitation medicine	①②③④⑤	NR
Feng (2017)	59.23 ± 7.108	58.10 ± 8.715	27 (20/7)	29 (22/7)	(1) Modified Da Chaihu decoction (100 ml,p.o,bid) (2) Antibiotic regimens based on drug sensitivity results	Antibiotic regimens based on drug sensitivity results	7 days	HAP	*Klebsiella pneumoniae*, *Pseudomonas aeruginosa*, *Acinetobacter baumannii*, *Staphylococcus aureus*, *Escherichia coli*, *serratia marcescens*	ICU	①②③⑥⑨	No adverse reactions in two groups
Guo (2017)	61.22 ± 14.02		22	40	(1) Nongdu Zheng decoction (NG,qd) (2) Tegacyclin ( first day 100 mg, and continue 50 mg,iv,bid); Imipenem And Cilastatin Sodium for Injection (1 g,iv,tid)	Tegacyclin (first day 100 mg, and continue 50 mg,iv,bid); Imipenem And Cilastatin Sodium for Injection (1 g,iv,tid)	7 days	NR	*Acinetobacter baumannii*	ICU	①②③④⑦⑧	NR
Hu (2017)	57.5 ± 9.17	57.22 ± 9.23	30 (18/12)	30 (18/12)	(1) Peitu Shengjin decoction (100 ml,po,qd) (2) Antibiotic regimens based on drug sensitivity results	Antibiotic regimens based on drug sensitivity results	14 days	CAP/HAP	*Klebsiella pneumoniae*, *Pseudomonas aeruginosa*, *Acinetobacter baumannii*, *Staphylococcus aureus*, *Escherichia coli*, *serratia marcescens*, *Stenotrophomonas maltophilia*, *Burkholderia cepacia*, *Proteus mirabilis*, *Staphylococcus epidermidis*, *Enterobacter cloacae*, *Enterococcus*	ICU	①②③④⑨	T: 4 cases (2 diarrhea, 2 Mild elevation of alanine aminotransferase) C: 3 case (3 Mild elevation of alanine aminotransferase) The 5 cases of Mild elevated alanine aminotransferase returned to normal after liver protection treatment
Shi (2017)	80.64 ± 6.22	80.85 ± 7.68	30 (17/13)	30 (19/11)	(1) Modified Baihu decoction(p.o,qd) (2) Antibiotic regimens based on drug sensitivity results	Antibiotic regimens based on drug sensitivity results	7 days	HAP	*Klebsiella pneumoniae*, *Pseudomonas aeruginosa*, *Acinetobacter baumannii*	Department of rehabilitation medicine	①③④	NR
Tan (2019)	60.34 ± 9.11	61.04 ± 8.69	43 (32/11)	43 (31/12)	(1) Modified Qingfei Huatan decoction (200 ml,p.o,bid) (2) Antibiotic regimens based on drug sensitivity results	Antibiotic regimens based on drug sensitivity results	14 days	NR	NR	NR	①③④⑤⑥	NR
Xie (2017)	62.2 ± 6.5	61.5 ± 6.2	67 (38/29)	67 (41/26)	(1) Feiyan Heji decoction (200 ml,p.o.,bid) (2) Linazolamide (300 ml,i.v.,bid)	Linazolamide (300 ml,i.v.,bid)	14 days	NR	*Staphyloccocus aureus Rosenbach*	NR	①②⑨	T: 1 case (1 nausea and vomiting) C: 4 cases (1 headache and fever, 1 Leukopenia, 1 thrombocytopenia)
Zhang (2018)	70.15 ± 5.33	71.15 ± 5.01	33 (18/15)	33 (19/14)	(1) Shashen Maidong decoction (100 ml,p.o.,tid) (2) Imipenem And Cilastatin Sodium for Injection (1 mg,iv,bid)	Imipenem And Cilastatin Sodium for Injection (1 mg,iv,bid)	14 days	VAP	NR	NR	①③④⑤⑥⑨	T: 11 cases (5 diarrhea, 3 rush, 4 Impaired renal function) C: 14 cases (4 diarrhea, 5 rush, 5 Impaired renal function)
Liang (2019)	51.04 ± 8.09	52.96 ± 8.52	28 (14/14)	28 (17/11)	(1) Qingwen Jiedu decoction (100 ml,p.o.,bid) (2) Meropenem (0.5 g,i.v.,tid); Tegacyclin (first day 100 mg, and continue 50 mg,iv,bid)	Meropenem for Injection (0.5 g,i.v.,tid); Tegacyclin for Injection (first day 100 mg, and continue 50 mg,iv,bid)	14 days	VAP	*Acinetobacter baumannii*	ICU	①②④⑧	NR
Liang (2019)	52.47 ± 9.25	48.17 ± 8.56	30 (14/16)	30 (18/12)	(1) Qingwen Jiedu decoction (200 ml,p.o.,bid) (2) Cefoperazone Sodium and Sulbactam Sodium for Injection (3 g,i.v.,tid); Amikacin sulfate for Injection (0.4 g,i.v.,qd)	Cefoperazone Sodium and Sulbactam Sodium for Injection (3 g,i.v.,tid); Amikacin sulfate for Injection (0.4 g,i.v.,qd)	7 days	HAP	*Pseudomonas aeruginosa*	ICU	①②⑤⑥⑦	NR
Liu (2019)	69.29 ± 6.18	68.98 ± 6.36	45 (29/16)	45 (28/17)	(1) Hongteng Zijin decoction (200 ml,p.o./NG,bid) (2) Cefoperazone Sodium and Sulbactam Sodium for Injection (3 g,i.v.,tid); Meropenem for Injection (1 g,i.v.,tid)	Cefoperazone Sodium and Sulbactam Sodium for Injection (3 g,i.v.,tid); Meropenem for Injection (1 g,i.v.,tid)	14 days	HAP	*Acinetobacter baumannii*	ICU	①②③④	NR
Peng (2019)	51.77 ± 2.84	50.44 ± 3.14	43 (21/22)	42 (22/20)	(1) Buzhong Yiqi decoction (150 ml,p.o.,bid) (2) Meropenem for Injection (1 g,i.v.,q8)	Meropenem for Injection (1 g,i.v.,q8)	14 days	NR	NR	NR	①	NR
Wang (2019)	53.6 ± 10.3	54.1 ± 9.8	40 (21/19)	40 (23/17)	(1) Traditional Chinese medicine decoction(po,bid) (2) Imipenem And Cilastatin Sodium for Injection (500 g,i.v.,tid)	Imipenem And Cilastatin Sodium for Injection (500 g,i.v.,tid)	14 days	NR	*Pseudomonas aeruginosa*	NR	①④⑤	NR
Xu (2019)	56.5 ± 14.8	53.7 ± 18.5	17(14/3)	17(13/4)	(1) Modified Mahuang Shengma decoction (150 ml,p.o./N.G.,bid) (2) minocycline for Injection (first day 0.4 g, and continue 0.2 g/d); Cefoperazone Sodium and Sulbactam Sodium for Injection (12 g/d); Piperacillin tazobactam for Injection (11.25 g/d)	Minocycline for Injection (first day 0.4 g, and continue 0.2 g/d); Cefoperazone Sodium and Sulbactam Sodium for Injection (12 g/d); Piperacillin tazobactam for Injection (11.25 g/d)	14 days	HAP	*Acinetobacter baumannii*	ICU	①②③⑤⑨	T: 2 cases (1 nausea and vomiting, 1 gastric regurgitation and intragastric residue > 100 ml) C: 1 case (1 nausea and vomiting)
Gao (2020)	77.53 ± 10.76	75.81 ± 11.59	30 (14/16)	30 (13/17)	(1) Fuzheng Huazhuo decoction (200 ml,p.o.,bid) (2) Piperacillin Sodium and Tazobactam Sodium and Moxifloxacin	Piperacillin Sodium and Tazobactam Sodium and Moxifloxacin	7 days	CAP	NR	ICU	①②③④⑤⑥	NR
Wang (2020)	55.82 ± 6.43	56.35 ± 5.82	43 (28/15)	43 (24/19)	(1) Mahuang Shengma decoction (150 ml,p.o./N.G.,bid) (2) Tegacyclin for Injection (first day 100 mg, and continue 50 mg,iv,q12; before the drug sensitivity test); Antibiotic regimens based on drug sensitivity results	Tegacyclin for Injection (first day 100 mg, and continue 50 mg,iv,q12; before the drug sensitivity test); Antibiotic regimens based on drug sensitivity results	14 days	NR	*Acinetobacter baumannii*	ICU	①②④⑤	NR
Xiao (2020)	71.19 ± 7.78	77.83 ± 7.69	46 (25/21)	46 (23/23)	(1) Qingjin Huatan decoction (100 ml,p.o.,bid) (2) Imipenem And Cilastatin Sodium for Injection	Imipenem And Cilastatin Sodium for Injection	14 days	NR	*Pseudomonas aeruginosa*	Department of Respiratory	①③⑤⑨	No adverse reactions in two groups
Yan (2020)	53.14 ± 24.56	52.15 ± 26.78	30 (21/9)	30 (22/8)	(1) Hongteng Zijin decoction (100 ml,p.o./NG,bid) (2) Cefoperazone Sodium and Sulbactam Sodium for Injection (3 g,i.v.,q8); Meropenem for Injection (1 g,i.v.,q8)	Cefoperazone Sodium and Sulbactam Sodium for Injection (3 g,i.v.,q8); Meropenem for Injection (1 g,i.v.,q8)	14 days	CAP	*Acinetobacter baumannii*	ICU	①③④⑤⑨	T: 3 cases (2 nausea, 1 vomiting) C: 6 cases (2 nausea, 2 vomiting, 1 rush, 1 thrombocytopenia, 1 Oliguria)
Zhu (2020)	74.55 ± 8.9	74.60 ± 9.68	49 (31/18)	48 (33/15)	(1) Xinjia Dayuan granule (p.o.,qid) (2) Empirical antibiotic therapy (before the drug sensitivity test); Antibiotic regimens based on drug sensitivity results	Empirical antibiotic therapy (before the drug sensitivity test); Antibiotic regimens based on drug sensitivity results	7 days	HAP	*Klebsiella pneumoniae*, *Acinetobacter baumannii*, *Escherichia coli*, *Pseudomonas aeruginosa*, *Taphyloccocus aureus Rosenbach*, *Stenotrophomonas maltophilia*, *Corynebacterium striatum*, *Serratia marcescens*, *Proteus mirabilis*	ICU/ER	①②③④⑤⑥⑨	No adverse reactions in two groups
Fu (2022)	64.31 ± 7.42	65.19 ± 7.86	30 (22/8)	30 (20/10)	(1) Qingfei Paidu decoction (150 ml,p.o.,bid) (2) Antibiotic regimens based on drug sensitivity results	Antibiotic regimens based on drug sensitivity results	10 days	NR	*Acinetobacter baumannii*	ICU/Department of Respiratory	①②④⑨	No adverse reactions in two groups
Xiao (2021)	65.31 ± 10.18	65.48 ± 10.22	30 (19/11)	30 (18/12)	(1) Xiao Chaihu decoction (150 ml,p.o.,bid) (2) Antibiotic regimens based on drug sensitivity results	Antibiotic regimens based on drug sensitivity results	10 days	HAP/VAP	*Acinetobacter baumannii*	ICU	①②③④⑤⑦⑧⑨	No adverse reactions in two groups
Yao (2021)	77.86 ± 8.01	76.60 ± 6.45	23 (13/10)	23 (11/12)	(1) Xue Duqing decoction (100 ml,p.o./NG,bid) (2) Antibiotic regimens based on drug sensitivity results	Antibiotic regimens based on drug sensitivity results	14 days	NR	*Pseudomonas aeruginosa*	ICU	①②③④⑤⑦	NR
Zhou (2021)	72.82 ± 4.53	72.49 ± 4.34	39 (25/14)	39 (25/14)	(1) Tongyang Xiezhuo decoction (150 ml,p.o.,bid) (2) Antibiotic regimens based on drug sensitivity results	Antibiotic regimens based on drug sensitivity results	14 days	HAP/VAP	*Klebsiella pneumoniae*, *Acinetobacter baumannii*, *Escherichia coli*	ICU	①④⑥	NR
Chen (2022)	58.62 ± 12.11	60.42 ± 14.06	60 (38/22)	60 (35/25)	(1) Fuzheng Guben decoction (200 ml,p.o./NG,bid) (2) Piperacillin Sodium and Tazobactam Sodium and Moxifloxacin	Piperacillin Sodium and Tazobactam Sodium and Moxifloxacin	14 days	NR	NR	NR	①③④⑤⑥	NR
Fang (2022)	73.69 ± 1.47	74.44 ± 1.35	36 (20/16)	34 (20/14)	(1) Modified Da Chaihu granule (9 g,p.o,bid) (2) conventional treatment (Antibiotics)	Conventional treatment (Antibiotics)	14 days	CAP/HAP	*Klebsiella pneumoniae*, *Pseudomonas aeruginosa*, *Acinetobacter baumannii*	ICU, Department of Respiratory and rehabilitation	①②③④⑤⑥⑨	T: 2 cases (2 nausea) C: 3 cases (2 diarrhea, 1 abdominal distension)
Li (2022)	62.75 ± 8.15	60.91 ± 7.06	64 (36/28)	64 (39/25)	(1) Ma Xingyin decoction (50 ml,p.o./NG,bid) (2) Tegacyclin for Injection (first day 100 mg, and continue 50 mg,iv,q12)	Tegacyclin for Injection (first day 100 mg, and continue 50 mg,iv,q12)	14 days	HAP/VAP	*Acinetobacter baumannii*	NR	①②⑨	T: 4 cases (2 nausea, 1 vomiting, 1 abdominal distension) C: 6 cases (2 nausea, 2 vomiting, 1 increased transaminase, 1 diarrhea)
Wang (2022)	58.29 ± 2.58	58.25 ± 2.53	34 (21/13)	34 (20/14)	(1) Traditional Chinese medicine decoction (100 ml,po,bid) (2) Antibiotic regimens based on drug sensitivity results	Antibiotic regimens based on drug sensitivity results	7 days	NR	NR	NR	①	NR
Chi (2022)	73.47 ± 9.043	74.4 ± 6.212	30 (16/14)	30 (12/18)	(1) Yiqi Huoxue Huatan decoction (100 ml,NG,bid) (2) Linazolamide (300 ml,i.v.,bid)	Conventional treatment (Antibiotics)	7 days	HAP/VAP	*Acinetobacter baumannii*	ICU/ER	①③④⑤⑦⑧⑨	No adverse reactions in two groups

ICU, intensive care unit; EICU, emergency intensive care unit; ER, emergency room; CAP, community-acquired pneumonia; HAP, hospital-acquired pneumonia; VAP, ventilator-associated pneumonia; T, treatment group; C, control group; NR, not reported; SD, standard deviation; po, per os; iv: intravenous; bid, bis in die; tid: ter in die. Outcome Indicators: ① Response rate, ② Microbiological response, ③ WBC, ④ PCT, ⑤ CRP, ⑥ Clinical Pulmonary Infection Scores (CPIS), ⑦ Acute Physiology and Chronic Health Evaluation Ⅱ(APACHE-Ⅱ), ⑧ Length of hospitalization, ⑨ Adverse event.

#### 3.2.1 Setting

Of the 38 studies, 22 were conducted in intensive care units (ICUs); 6 were conducted in non-ICU settings, including neurology, rehabilitation medicine, respiratory, and integrated traditional Chinese and Western medicine wards, and 10 RCTs did not provide information on the setting.

#### 3.2.2 Pathogens

The studies involved MDR/XDR *P. aeruginosa*, MDR/XDR *Acinetobacter baumanii* (*A. baumannii*), MRSA, ESBL-producing *Escherichia coli*, and mixed MDR/XDR bacteria. Nine RCTs did not provide information on pathogen type.

#### 3.2.3 Treatment

Regarding treatment groups, only one study adopted CHC monotherapy, whereas the others used combined interventions. CHC therapies were prepared as decoctions or granules. Twenty-three RCTs included patients who received Chinese herbal compound prescriptions orally, while the remaining studies involved patients receiving these prescriptions via nasogastric feeding. All of these patients were from ICUs. Eighteen studies documented antibiotic treatment regimens based on sputum-culture drug-sensitivity test results or recommended guidelines, while the remaining 20 studies provided detailed documentation of the types and dosages of antibiotics administered in treatment. The antibiotics involved in these studies included carbapenems, β-lactam antibiotics, glycopeptides, aminoglycosides, oxazolidinones, and tetracyclines. The most frequently utilized antibiotic was a combination of imipenem and cilastatin sodium.

#### 3.2.4 Outcome

All 38 studies included reported response rates. Regarding secondary outcomes, 55.26%, 47.37%, 60.53%, 47.37%, 28.95%, 18.42%, 15.79%, and 44.74% of the included RCTs reported microbiological response, WBC count, PCT levels, CRP levels, CPIS, APACHE-II score, length of hospitalization, and safety, respectively.

### 3.3 Risk of bias assessment

**FIGURE 2:**
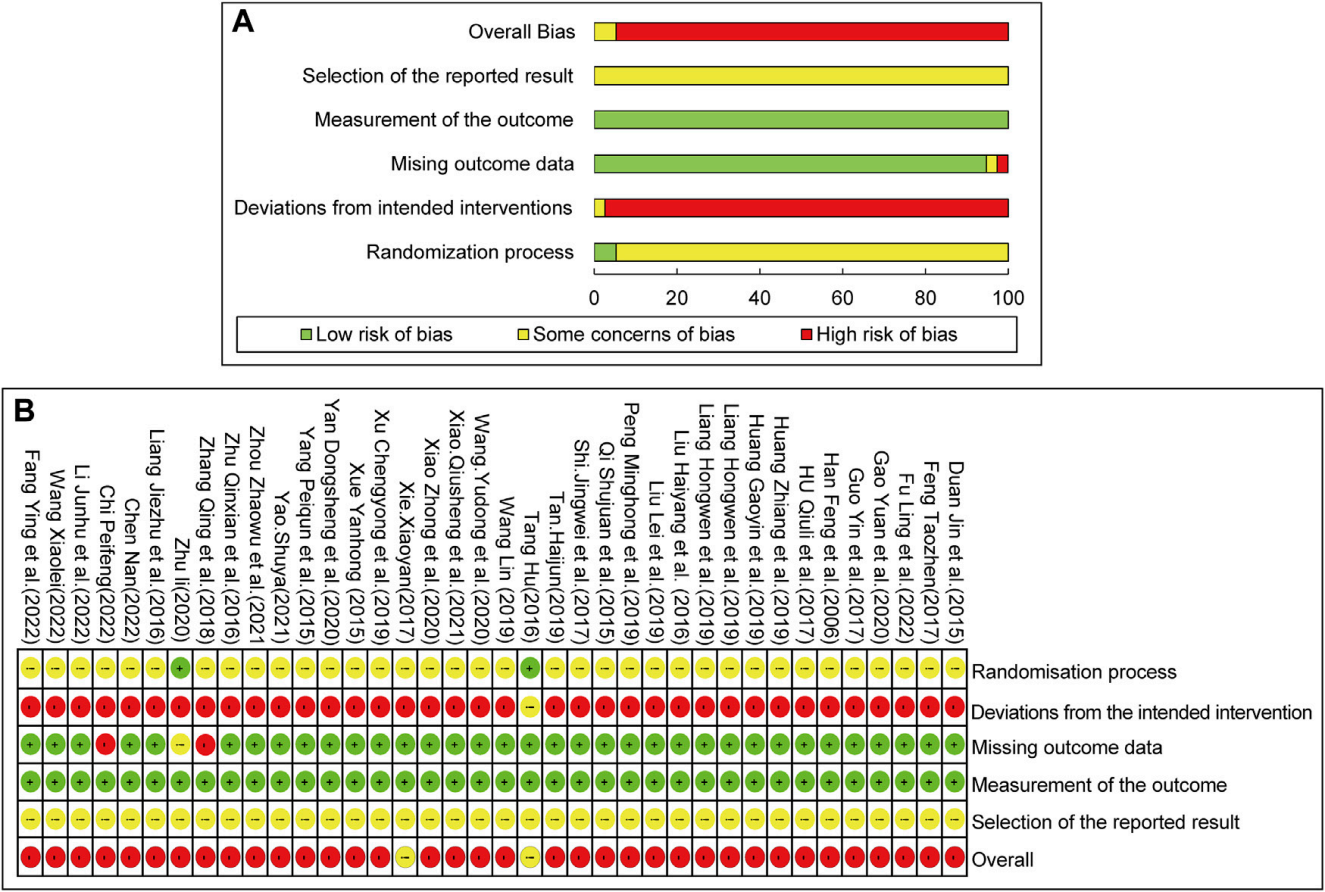
Risk of bias assessment for included studies: **(A)** Risk of bias graph. **(B)** Risk of bias summary.

The Rob 2.0 tool was used to assess the quality of the included studies. The results of the risk of bias assessment are as follows: 1) Randomization process: All included studies declared randomization, but only 25 studies described the details of the randomization methods (by software or random number table). The allocation concealment approach was illustrated by only two studies. None of the studies exhibited baseline differences between the intervention and control groups, indicating poor randomization. Consequently, two studies were assessed as low-risk, and the rest had some concerns about the risk of bias. 2) Deviations from the intended interventions: As only one study adopted a single-blind method, and none of the studies used intention-to-treat (ITT) or modified-ITT analysis to evaluate differences between the intervention and control groups, the risk of bias associated with deviation from the intended interventions was rated as high for most studies. 3) Missing outcome data: Two studies reported missing outcome data due to dropouts. The rest of the included RCTs had no incomplete data and were marked as low-risk. 4) Measurement of the outcome: All studies were rated as having a low risk of bias, mostly because participants’ symptoms and laboratory results were used to assess response rates, which were rarely influenced by their knowledge. 5) Selection of the reported result: As none of the included studies had a registered protocol, adherence to pre-planned statistical analysis methods could not be assessed. Consequently, all included studies were assessed as having unclear risks. The overall risk of bias was high. The summary of methodological quality is displayed in Figure 2.

### 3.4 Primary outcomes

#### 3.4.1 Response rate

Thirty-eight studies involving 2890 patients compared CHC + antibiotic treatment with antibiotic-only treatment and reported the response rate for treating pneumonia caused by MDR or XDR bacteria. The heterogeneity test results revealed very low heterogeneity among studies (X^2^ = 38.33; *p* = 0.41; I^2^ = 3.47%), and therefore a fixed-effects model was used for the statistical analysis of the response rate. The meta-analysis revealed a significant response rate for CHC + antibiotic treatment (response rate = 1.24; 95% CI: 1.19–1.28; *p* < 0.0001). Subgroup analysis according to the average age, treatment duration, sample size, setting, infection type, and pathogen (Table 2, Supplementary Material S4) revealed statistically significant differences between CHC + antibiotic treatment and antibiotic-only treatment in the average age, treatment duration, sample size, and setting subgroups. In contrast, subgroup analysis of various infection types revealed no statistical difference for patients with CAP. Sensitivity analyses, in which single study was systematically excluded to determine whether one study had a significant impact on the results, indicated similar aggregated statistics and robust results (Figure 4, Supplementary Material S6).

**TABLE 2:** Subgroup analysis of response rate according to average age, treatment duration, sample size, setting, infection type, and pathogen.

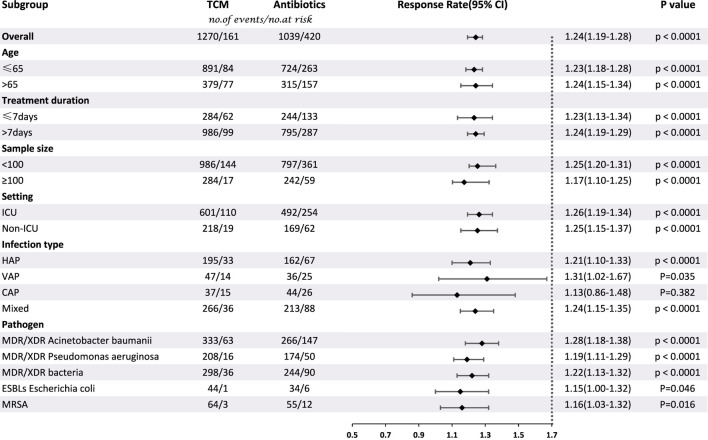

### 3.5 Secondary outcomes

#### 3.5.1 Microbiological response

Twenty-one studies involving 1542 patients reported the efficacy of CHC + antibiotic *versus* antibiotic-only treatment for microbiological eradication. The heterogeneity test revealed low heterogeneity among studies (X^2^ = 23.97; *p* = 0.24; I^2^ = 16.55%); Therefore, the statistical analysis of the microbiological response was performed using a fixed-effects model. The results indicated that CHC + antibiotic treatment significantly eradicated bacteria compared with antibiotic-only treatment (RR = 1.41; 95% CI: 1.27–1.57; *p* < 0.0001). Subgroup analysis according to total sample size, setting, and infection type revealed no statistical improvement for the large sample size, non-ICU setting, and CAP subgroups. In contrast, among the other subgroups, CHC + antibiotic treatment exhibited statistically superior bactericidal effects when compared with antibiotic-only treatment (Table 3, Supplementary Material S4). MRSA eradication in the CHC + antibiotic group was lower than that in the antibiotic-only group, although the difference was not statistically significant. However, as the number of studies within the MRSA subgroup was limited, further investigation is required. Sensitivity analysis indicated that the pooled effect was robust (Figure 4, Supplementary Material S6).

**TABLE 3:** Subgroup analysis of microbiological response according to average age, treatment duration, sample size, setting, infection type, and pathogen.

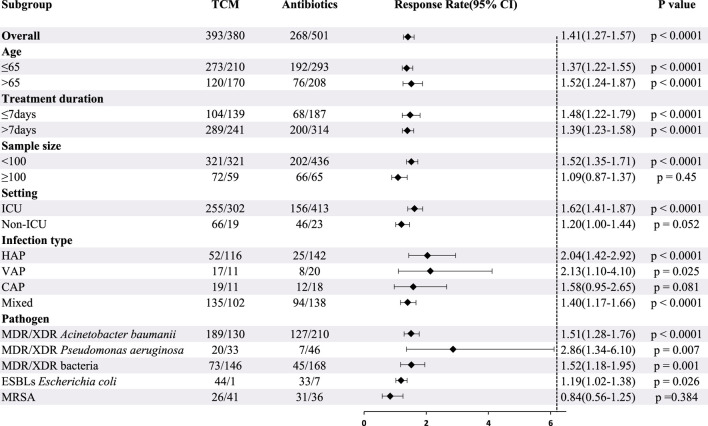

#### 3.5.2 White blood cell count

Eighteen studies involving 1284 patients reported the WBC count for CHC + antibiotic treatment and antibiotic-only treatment groups. Heterogeneity tests revealed high heterogeneity among the studies (X^2^ = 261.52; *p* < 0.0001; I^2^ = 93.49%), and statistical analysis of this outcome was conducted using a random-effects model. The results indicated superior efficacy of CHC + antibiotics in reducing the WBC count compared with that of the control group (MD: −2.09; 95% CI: −2.65 to −1.53; *p* < 0.0001). Meta-regression indicated no significant difference in WBC count based on average age (*p* = 0.055, adjusted R^2^ = 26.77%), publication year (*p* = 0.603, adjusted R^2^ = −7.77%), sample size (*p* = 0.105, adjusted R^2^ = 10.52%), or treatment duration (*p* = 0.933, adjusted R^2^ = −10.40%) (Supplementary Material S5). Although study characteristics, such as setting, infection type, and pathogen, may have also contributed to heterogeneity, meta-regression analyses were not possible due to incomplete documentation of these baseline characteristics. Subgroup analysis revealed undiminished heterogeneity within each subgroup, indicating that the heterogeneity could not be explained by setting, infection type, or pathogen. Furthermore, subgroup analysis of various settings revealed no statistical difference between CHC + antibiotics and antibiotics only in the treatment of non-ICU patients (Supplementary Material S4). Sensitivity analysis indicated that the pooled effect was robust (Figure 4, Supplementary Material S6).

#### 3.5.3 Procalcitonin levels

Twenty-three studies involving 1693 patients reported PCT levels for CHC + antibiotic and antibiotic-only groups. The heterogeneity test revealed high heterogeneity among the studies (X^2^ = 491.60; *p* < 0.0001; I^2^ = 95.52%), and statistical analysis of this outcome was conducted using a random-effects model. The results revealed a significantly lower PCT level in the CHC + antibiotic group than in the antibiotic-only group (MD = −0.49; 95% CI: −0.59 to −0.40; *p* < 0.0001). Meta-regression revealed no significant difference in PCT level based on average age (*p* = 0.92, adjusted R^2^ = −7.22%), publication year (*p* = 0.485, adjusted R^2^ = −4.17%), sample size (*p* = 0.080, adjusted R^2^ = 12.78%), or treatment duration (*p* = 0.831, adjusted R^2^ = −6.49%) (Supplementary Material S5). Subgroup analysis of various pathogens, settings, and infection types indicated that these factors were not the source of heterogeneity. Subgroup analysis of various infection types revealed that, although no statistical difference was seen between the CHC + antibiotic and antibiotic-only groups for patients with VAP, CHC + antibiotics significantly reduced the PCT level in patients with HAP, CAP, or a mixed infection type compared with antibiotics only (Supplementary Material S4). Sensitivity analysis indicated that the pooled effect was robust (Figure 4, Supplementary Material S6).

#### 3.5.4 C-reactive protein levels

Eighteen studies involving 1282 patients reported the CRP levels for CHC + antibiotic and antibiotic-only groups. High heterogeneity among studies was detected (X^2^ = 1733.56; *p* < 0.0001; I^2^ = 99.02%), and statistical analysis of this outcome was therefore conducted using a random-effects model. The results revealed significantly lower CRP levels in the CHC + antibiotic group than those in the antibiotic-only group (MD = −11.80; 95% CI: −15.22 to −8.39; *p* < 0.0001). Meta-regression revealed no significant difference in CRP levels based on average age (*p* = 0.958, adjusted R^2^ = −8.57%), publication year (*p* = 0.062, adjusted R^2^ = 22.64%), sample size (*p* = 0.092, adjusted R^2^ = 14.66%), or treatment duration (*p* = 0.355, adjusted R^2^ = −0.24%) (Supplementary Material S5). Subgroup analysis indicated that the pathogen, setting, and infection type were not the source of heterogeneity. Subgroup analysis of various pathogens revealed that there was no statistically significant difference between CHC + antibiotic treatment and antibiotic treatment only in patients with pneumonia caused by MDR *P. aeruginosa*, whereas CHC + antibiotics had a superior outcome for treating pneumonia caused by mixed MDR/XDR bacteria and MDR/XDR *A. baumannii* than antibiotics alone (Supplementary Material S4). Sensitivity analysis indicated that the pooled effect was robust (Figure 4, Supplementary Material S6).

#### 3.5.5 Clinical pulmonary infection scores

Eleven studies involving 898 patients reported CPISs for CHC + antibiotic and antibiotic-only groups. High heterogeneity (X^2^ = 332.03; *p* < 0.0001; I^2^ = 94.66%) was detected among the studies, and statistical analysis of this outcome was conducted using a random-effects model. The results indicated that CHC + antibiotics could significantly lower the CPIS more than antibiotics alone (MD = −1.97; 95% CI: −2.68 to −1.26; *p* < 0.0001). Meta-regression revealed no significant association between CPIS and average age (*p* = 0.608, adjusted R^2^ = −10.20%), publication year (*p* = 0.504, adjusted R^2^ = −7.70%), sample size (*p* = 0.240, adjusted R^2^ = 8.05%), or treatment duration (*p* = 0.399, adjusted R^2^ = −3.90%) (Supplementary Material S5). The source of heterogeneity could not be explained by the pathogen, setting, or infection type. Subgroup analysis based on the pathogen type revealed a superior outcome for CHC + antibiotics in the treatment of pneumonia caused by MDR *P. aeruginosa* but not for pneumonia caused by mixed MDR/XDR bacteria (Supplementary Material S4). Sensitivity analysis indicated that the pooled effect was robust (Figure 4, Supplementary Material S6).

#### 3.5.6 APACHE-II score

Seven studies involving 424 patients reported APACHE-II scores for CHC + antibiotic and antibiotic-only treatment. High heterogeneity among the studies (X^2^ = 16.62; *p* = 0.011; I^2^ = 63.91%) was detected, and statistical analysis of this outcome was conducted using a random-effects model. The results indicated that the CHC + antibiotic group had lower APACHE-II scores than did the antibiotic-only group (MD = −4.08; 95% CI: −5.16 to −3.00; *p* < 0.0001). Subgroup analysis showed statistical differences in the average age, treatment duration, pathogen, and infection type subgroups. However, as the number of studies within each subgroup was limited, the impact of these characteristics on the APACHE-II score could not be assessed (Supplementary Material S4). Sensitivity analysis indicated that the pooled effect was robust (Figure 4, Supplementary Material S6).

#### 3.5.7 Length of hospitalization

Six studies involving 394 patients reported the length of hospitalization for CHC + antibiotic and antibiotic-only groups. High heterogeneity among the studies (X^2^ = 27.26; *p* < 0.0001; I^2^ = 81.66%) was detected, and statistical analysis of this outcome was conducted using a random-effects model. The results revealed a shorter length of hospitalization for the CHC + antibiotic group than for the antibiotic-only group (MD = −4.79; 95% CI: −6.18 to −3.40; *p* < 0.0001). Although subgroup analyses by age, duration of treatment, pathogen, setting, and type of infection were conducted, the limited number of studies within each subgroup prevented an assessment of the impact of these characteristics on the length of hospitalization (Supplementary Material S4). Sensitivity analysis indicated that the pooled effect was robust (Figure 4, Supplementary Material S6).

#### 3.5.8 Safety

**FIGURE 3:**
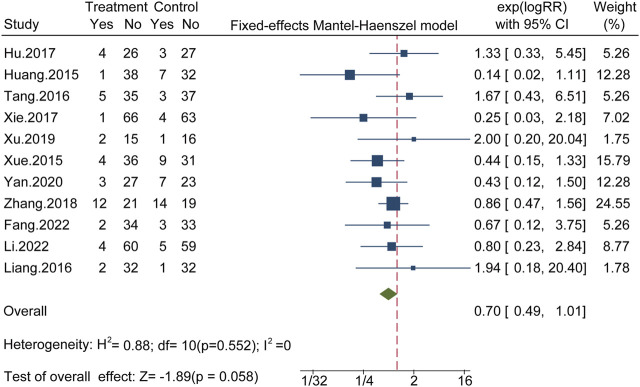
The forest plot of rate of adverse events.

**FIGURE 4:**
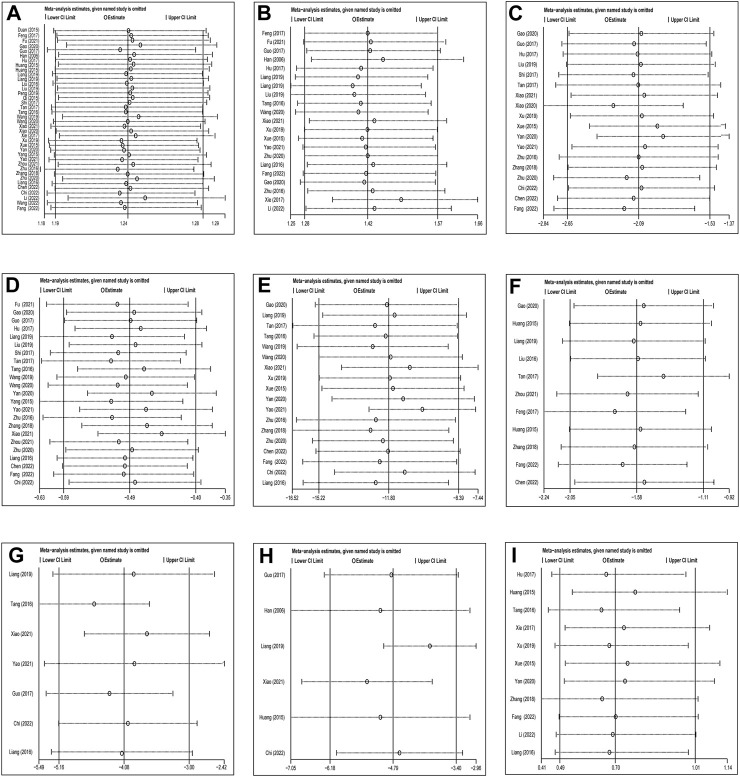
Sensitivity analysis: CHC with antibiotics vs. Antibiotics: **(A)** Response rate, **(B)** Microbiological response, **(C)** WBC, **(D)** PCT, **(E)** CRP, **(F)** CPIS scores, **(G)** APACHE‐II, **(H)** Length of hospitalization, **(I)** Adverse events.

Eleven studies involving 859 patients reported AEs, with fewer AEs reported for the CHC + antibiotic group than for the antibiotic-only group. All 11 studies reported gastrointestinal tract symptoms, such as nausea, vomiting, and diarrhea. In one study, four cases of renal dysfunction were reported in the CHC + antibiotic group. Six studies claimed that, during the study, there were no AEs in the CHC + antibiotic group. AEs were not mentioned in the remaining 21 studies. No heterogeneity was detected among the studies (X^2^ = 8.79; *p* = 0.552; I^2^ = 0%), and statistical analysis of this outcome was conducted using a fixed-effects model. The results indicated fewer AEs in the CHC + antibiotic group than in the antibiotic-only group; However, the difference was not statistically significant (RR = 0.70; 95% CI: 0.49–1.01; *p* = 0.058) (Figure 3). Sensitivity analysis indicated that the pooled effect was robust (Figure 4, Supplementary Material S6).

### 3.6 Publication bias

**FIGURE 5:**
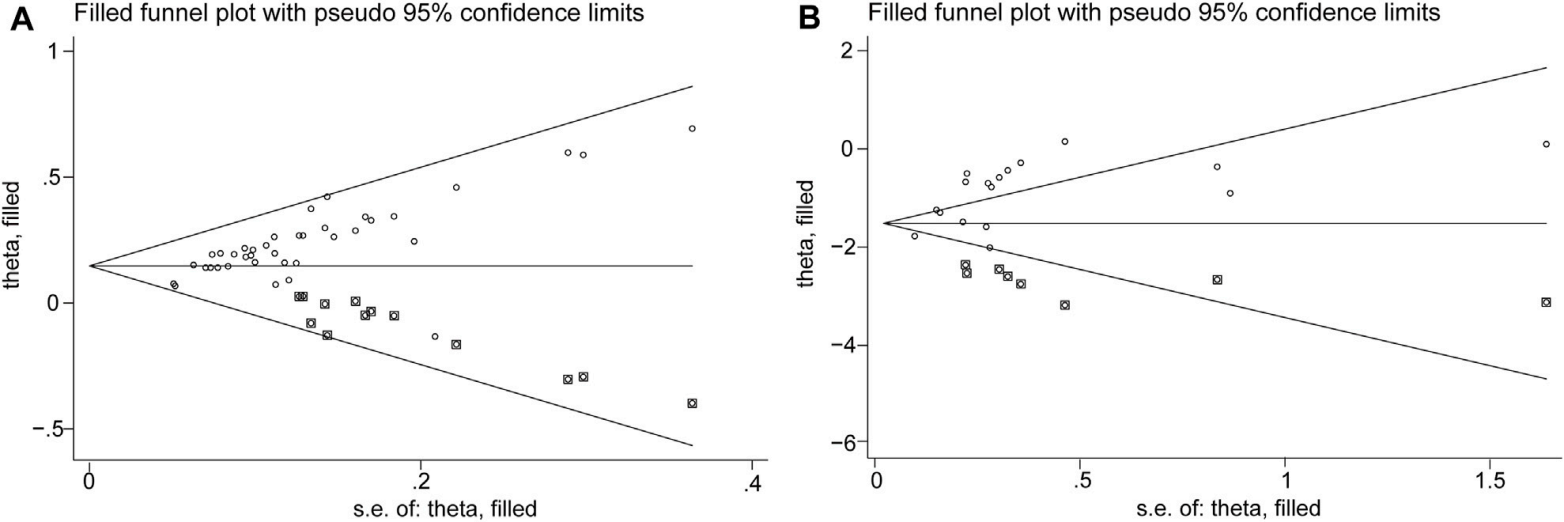
Trim-and-fill analysis for **(A)** Response rate, **(B)** Microbiological response.

We assessed the impact of publication bias on response rate using Begg’s and Egger’s tests. The *p*-value for both Begg’s and Egger’s tests were <0.0001, suggesting publication bias. However, trim-and-fill analysis performed using the fixed-effects model did not reveal any publication bias. For the microbiological response, the *p*-values for Begg’s and Egger’s tests were 0.266 and 0.004, respectively, which were not indicative of publication bias. This was supported by trim-and-fill analysis, which also did not reveal any publication bias (Figure 5, Supplementary Material S7). According to Begg’s test, there was no evidence of publication bias for “WBC count,” “PCT level,” “CRP level,” “CIPS score,” “APACHE-II score,” “length of hospitalization,” and “safety.” However, the results of Egger’s test indicated a potential risk of publication bias for “WBC count,” “CRP level,” and “CIPS score.”

### 3.7 Quality of evidence according to outcome measures

The GRADE method was used to assess the quality of the evidence. When comparing CHC + antibiotics with antibiotics-only, the overall quality of evidence for each outcome was evaluated as moderate to very poor. The high risk of bias, inconsistency across studies, and imprecision of findings contributed to the low quality of evidence. Supplementary Material S8 provides a summary of the overall quality of evidence for each outcome.

### 3.8 Trial sequential analysis

The false positive and false negative findings in systematic reviews and meta-analyses can be controlled by TSA, which has therefore become an attractive statistical method. Therefore, we used TSA 0.9.5.10 beta software to ensure that the results of our meta-analyses were reliable and conclusive. The required information size in this study was estimated by α = 0.05 (two-sided) and β = 0.20 (power of 80%), based on the O'Brien–Fleming alpha-expenditure function. The RR reduction and event rates in both experimental and control groups were calculated from the mean of the event proportions.

#### 3.8.1 Response rate

**FIGURE 6:**
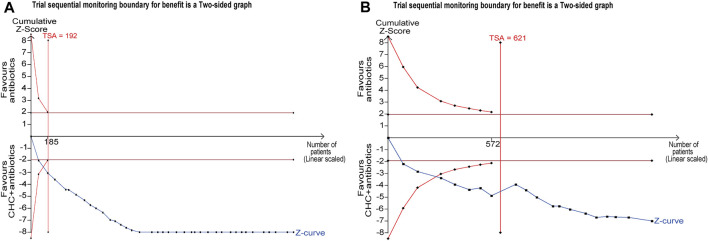
Trial sequential analysis (TSA) for response rate and microbiological response. **(A)** TSA of response rate with a control event proportion of 71.21%, type 1 error of 5%, power of 80%, and relative risk reduction of −23%. **(B)** TSA of microbiological response with a control event proportion of 34.40%, type 1 error of 5%, power of 80%, and relative risk reduction of −41%.

The TSA included 38 RCTs and was performed based on a controlled-event proportion of 71.21% and an RR reduction of −23%. The TSA identified the required information size (RIS) as 192. The cumulative Z-curve reached the RIS and crossed the RIS-adjusted boundary in support of CHC + antibiotic treatment, thus suggesting conclusive and robust results (Figure 6).

#### 3.8.2 Microbiological response

The TSA included 21 RCTs and was performed based on a controlled-event proportion of 34.40% and an RR reduction of −41%. The RIS was 621. However, the number of patients did not reach the RIS, and the cumulative Z-curve crossed the conventional boundary line and RIS-adjusted boundary, indicating that CHC + antibiotic treatment was effective in eradicating MDR or XDR bacteria in patients with pneumonia (Figure 6).

### 3.9 Apriori algorithm-based association rule analysis

**FIGURE 7:**
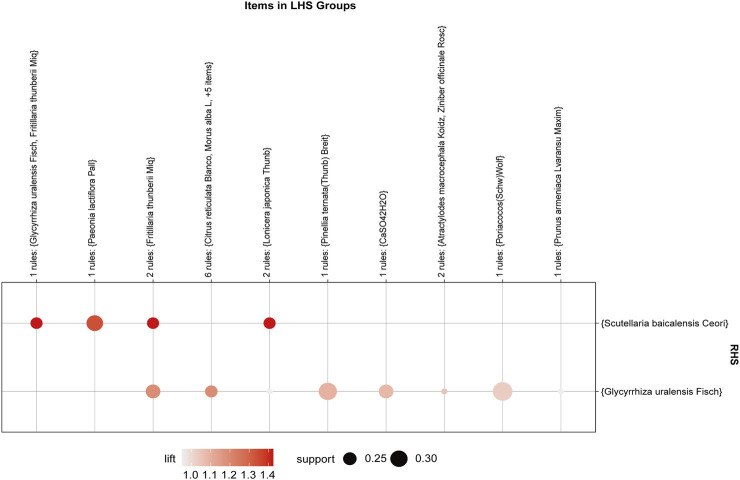
Grouping matrix of the association rules in the meta-analysis of Chinese herbs for pneumonia caused by MDR or XDR bacteria. The herb X and herb Y are called antecedent (left-hand side, LHS) and consequent (right-hand side, RHS) of the rules.

**FIGURE 8:**
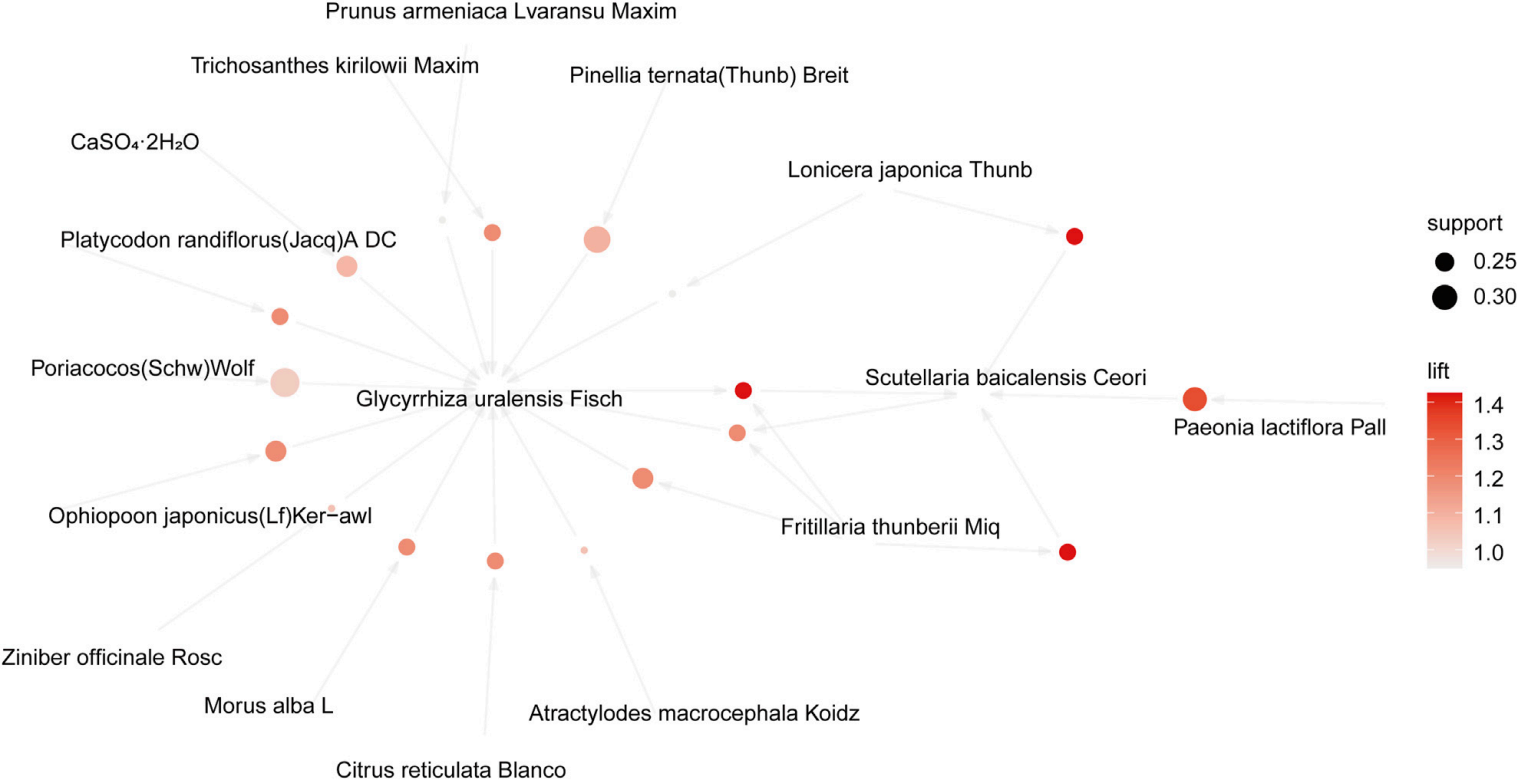
Network graph of the association rules in the meta-analysis of Chinese herbal compound prescribed for pneumonia caused by MDR or XDR bacteria.

The association rule analysis included 110 herbs from 38 CHC prescriptions. CHC prescriptions are summarized in Table 4, and the distribution frequency of the herbs is summarized in Table 5. Eighteen association rules were detected when the minimum support and confidence values were set at 0.2 and 0.8, respectively. The lift was >1, which was indicative of effective association rules, with a higher degree of lift indicative of a stronger association. The scatter plot reflected the degree of lift, indicated by color depth, of each association rule, ranging from 0.950 to 1.425 (Table 6). The general distribution of the determined association rules was evaluated using a grouping matrix (Figure 7). The horizontal ordinate represented 10clusters, which were generated by 18 association rules. According to the scatter plot and grouping matrix diagram, four association rules of {*Paeonia lactiflora* Pall [Ranunculaceae]}=>{*Scutellaria baicalensis* Georgi [Labiatae]}, {*Fritillaria thunbergii* Miq [Liliaceae]}=>{*S. baicalensis* Georgi [Labiatae]}, {*Lonicera japonica* Thunb [Caprifoliaceae]}=>{*S. baicalensis* Georgi [Labiatae]}, and {*F. thunbergii* Miq [Liliaceae], *Glycyrrhiza uralensis* Fisch [Fabaceae]}=>{*S. baicalensis* Georgi [Labiatae]} were considered. The three rules with the highest lift were {*F. thunbergii* Miq [Liliaceae]}=>{*S. baicalensis* Georgi [Labiatae]}, {*L. japonica* Thunb [Caprifoliaceae]}=>{*S. baicalensis* Georgi [Labiatae]}, and {*F. thunbergii* Miq [Liliaceae], *G. uralensis* Fisch [Fabaceae]}=>{*S. baicalensis* Georgi [Labiatae]}, which also exhibited a confidence value exceeding 0.9. Therefore, *S. baicalensis* Georgi [Labiatae], *F. thunbergii* Miq [Liliaceae], *L. japonica* Thunb [Caprifoliaceae], and *G. uralensis* Fisch [Fabaceae] were identified as core CHC ingredients for the treatment of patients with pneumonia caused by MDR or XDR bacteria. A network graph presenting the relationship between these association rules is shown in Figure 8.

**TABLE 4:** CHC ingredients used in the included studies.

Study	Prescription name	Ingredients of CHC prescription (Latin name)	Ingredients of CHC prescription (Scientific name)
Duan (2015)	TCM formula for individual research	Ephedrae Herba 10g,Cimicifugae Rhizoma 12g,Gypsum Fibrosum 30g,Angelicae Sinensis Radix 12g,Magnoliae Officinalis Corte×10g,Armeniacae semen amarum 10g,Polygonati Odorati Rhizoma 15g,Radix Ophiopogonis 20g,Paeoniae Radix Alba 15g,Codonopsis Radix 20g,Glycyrrhizae radix et rhizoma 6g	*Ephedra sinica Stapf* [Ephedraceae] 10g,*Cimicifuga heracleifolia* Kom [Ranunculaceae] 12g,CaSO4 2H2O 30g,*Angelica sinensis* (Oliv) Diels [Apiaceae] 12g,*Magnolia officinalis* Rehdet Wils [Magnoliaceae] 10g,*Prunus armeniaca* Lvaransu Maxim [Rosaceae] 10g, *Polygonatumodoratum* (Mill) Druce [Liliaceae] 15g,*Ophiopogon japonicus* (Lf) Ker-Gawl [Liliaceae] 20g,*Paeonia lactiflora* Pall [Ranunculaceae] 15g,*Codonopsis tangshen* Oliv [Campanulaceae] 20g,*Glycyrrhiza uralensis* Fisch [Fabaceae] 6g
Feng (2017)	Modified Da Chaihu decoction	Bupleuri Radix 30g,Scutellariae Radix 18g,Paeoniae Radix Alba 30g,Pinelliae rhizoma 12g,Arisaema cum bile 12g,Aurantii FructusImmaturus 12g,Zingiberis Rhizoma Recens 6g,Jujubae Fructus 6g,Rhei Radix Et Rhizoma 15g,Glycyrrhizae radix et rhizoma 6g	*Bupleurum chinensis* DC 30g,*Scutellaria baicalensis* Ceorgi [Labiatae] 18g,*Paeonia lactiflora* Pall [Ranunculaceae] 30g,*Pinellia ternata* (Thunb) Breit [Araceae] 12g,Dannaxing 12g,*Citrus aurantium* L [Rutaceae] 12g,*Zingiber officinale* Rosc [Zingiberaceae] 6g,*Ziziphus jujuba* Mill [Rhamnaceae] 6g,*Rheum officinale* Bail1 [Polygonaceae] 15g,*Glycyrrhiza uralensis* Fisch [Fabaceae] 6g
Fu (2022)	Qingfei Paidu decoction	Scutellariae Radix 15g,Armeniacae semen amarum 10g,Platycodonis Radix 12g,Trichosanthis Fructus 15g,Pinelliae rhizoma 12g,Pheretima 10g,Forsythiae Fructus 15g,Mori Cortex 12g,Citri exocarpium rubrum 15g,Poria 10g,Fritillariae Thunbergii Bulbus 10g,Descurainiae semen lepidii semen 10g,Perillae fructus 10g,Pseudostellariae Radix 30g,Radix Ophiopogonis 10g,Schisandrae chinensis fructus 5g,Glycyrrhizae radix et rhizoma 6g	*Scutellaria baicalensis* Ceorgi [Labiatae] 15g,*Prunus armeniaca* Lvaransu Maxim [Rosaceae] 10g,*Platycodon grandiflorus* (Jacq) A DC [Campanulaceae] 12g,*Trichosanthes kirilowii* Maxim [Cucurbitaceae] 15g,*Pinellia ternata* (Thunb) Breit [Araceae] 12g,*Pheretima aspergillum* (E Perrier) [Megascolecidae] 10g,*Forsytniasuspensa* (Thunb) Vahl [Oleaceae Hoffmanns & Link] 15g,*Morus alba* L [Moraceae] 12g,*Citrus reticulata* Blanco [Rutaceae] 15g,*Poriacocos* (Schw) Wolf [Polyporaceae] 10g,*Fritillaria thunbergii* Miq [Liliaceae] 10g,*Descurainia sophia* (L) Webb ex Prantl [Brassicaceae] 10g,*Perilla frutescens* (L) Britt [Labiatae] 10g,*Pseudostellaria heterohylla* (Miq) pax ex pax et Hoffm [Caryophyllaceae] 30g,*Ophiopogon japonicus* (Lf) Ker-Gawl [Liliaceae] 10g,*Schisandra chinensis* (Turcz) Baill [Magnoliaceae] 5g,*Glycyrrhiza uralensis* Fisch [Fabaceae] 6g
Gao (2020)	Fuzheng Huazhuo decoction	Astragali Radix 30g,Ginseng Root and Rhizome 12g,Rehmanniae radix praeparata 15g,Poria 15g,Mori Cortex 15g,Asteris radix et rhizoma 15g,Radix Ophiopogonis 15g,Citri Reticulatae Pericarpium 15g,Angelicae Sinensis Radix 10g,Dioscoreae Rhizoma 12g,Corni fructus 12g,Cinnamomi cortex 6g,Schisandrae chinensis fructus 5g,Glycyrrhizae radix et rhizoma 10g,Zingiberis Rhizoma Recens 10g,Jujubae Fructus 6g	*Astragalus memeranaceus* (Fisch) Bge Var mongholicus (Bge) Hsiao [Fabaceae] 30g,*Panaxginseng* CAMey 12g,*Rehmannia glutinosa* Libosch [Scrophulariaceae] 15g,*Poriacocos* (Schw) Wolf [Polyporaceae] 15g,*Morus alba* L [Moraceae] 15g,*Aster tataricus* Lf [Compositae] 15g,*Ophiopogon japonicus* (Lf) Ker-Gawl [Liliaceae] 15g,*Citrus reticulata* Blanco [Rutaceae] 15g,*Angelica sinensis* (Oliv) Diels [Apiaceae] 10g,*Dioscorea opposita* Thunb [Dioscoreaceae] 12g,*Cornus officinalis* Sieb et Zucc [Cornaceae] 12g,*Cinnamomum cassia* Presl [Lauraceae] 6g,*Schisandra chinensis* (Turcz) Baill [Magnoliaceae] 5g,*Glycyrrhiza uralensis* Fisch [Fabaceae] 10g,*Zingiber officinale* Rosc [Zingiberaceae] 10g,*Ziziphus jujuba* Mill [Rhamnaceae] 6g
Guo (2017)	Nongdu Zheng decoction	Astragali Radix 30g,Pseudostellariae Radix 15g,Ophiopogonis 15g,Scutellariae Radix 15g,Gypsum Fibrosum 30g,Rhei Radix Et Rhizoma 15g,Moutan Cortex 15g,Coptidis Rhizoma 5g,Anemarrhenae Rhizoma 10g,Platycodonis Radix 15g,Mori Cortex 15g,Saigae tataricae cornu 15g,Glycyrrhizae radix et rhizoma 5g	*Astragalus memeranaceus* (Fisch) Bge Var mongholicus (Bge) Hsiao [Fabaceae] 30g,*Pseudostellaria heterohylla* (Miq) pax ex pax et Hoffm [Caryophyllaceae] 15g,*Ophiopogon japonicus* (Lf) Ker-Gawl [Liliaceae] 15g,*Scutellaria baicalensis* Ceorgi [Labiatae] 15g,CaSO4 2H2O 30g,*Rheum officinale* Bail1 [Polygonaceae] 15g,*Paeonia suffruticosa* Andr [Ranunculaceae] 15g,*Coptis chinensis* Franch [Ranunculaceae] 5g,*Anemarrhena asphodeloides* Bge [Liliaceae] 10g,*Platycodon grandiflorus* (Jacq) A DC [Campanulaceae] 15g,*Morus alba* L [Moraceae] 15g,*Saiga tatarica* Linnaeus [Bovidae] 15g,*Glycyrrhiza uralensis* Fisch [Fabaceae] 5g
HU (2017)	Peitu Shengjin decoction	Rhodiolae crenulatae radix et rhizoma 10g,Codonopsis Radix 15g,Poria 15g,Citri Reticulatae Pericarpium 12g,Pinelliae rhizoma 9g,Asteris radix et rhizoma 10g,Ophiopogonis 10g,Mori Cortex 10g,Gypsum Fibrosum 20g,Chuanxiong Rhizoma 10g,Platycodonis Radix 10g,Glycyrrhizae radix et rhizoma 6g	*Rhodiola crenulata* (Hook f et Thoms)H Ohba [Crassulaceae] 10g,*Codonopsis tangshen* Oliv [Campanulaceae] 15g,*Poriacocos* (Schw)Wolf [Polyporaceae] 15g,*Citrus reticulata* Blanco [Rutaceae] 12g,*Pinellia ternata* (Thunb) Breit [Araceae] 9g,*Aster tataricus* L f [Compositae] 10g,*Ophiopogon japonicus* (Lf) Ker-Gawl [Liliaceae] 10g,*Morus alba* L [Moraceae] 10g,CaSO4 2H2O 20g,*Ligusticum chuanxiong* Hort [Apiaceae] 10g,*Platycodon grandiflorus* (Jacq) A DC [Campanulaceae] 10g,*Glycyrrhiza uralensis* Fisch [Fabaceae] 6g
Huang (2019)	Xia Yuhuang decoction	Houttuyniae Herba 30g,Coptidis Rhizoma 10g,Scutellariae Radix 15g,Phellodendri Chinensis Cortex 15g,Prunellae Spica 30g	*Houttuynia cordata* thunb [Saururaceae] 30g,*Coptis chinensis* Franch [Ranunculaceae] 10g,*Scutellaria baicalensis* Ceorgi [Labiatae] 15g,*Phellodendron chinense* Schneid [Rutaceae] 15g,*Prunella vulgaris* L [Labiatae] 30g
Huang (2019)	Qingfei Tongluo decoction	Fagopyri dibotryis rhizoma 30g,Astragali Radix 20g,Belamcandae Rhizoma 15g,Codonopsis Radix 15g,Lonicerae Japonicae 15g,Chuanxiong Rhizoma 15g,Pseudostellariae Radix 15g,Scutellariae Radix 15g,Armeniacae semen amarum 12g,Poria 12g,Fritillariae Cirrhosae Bulbus 10g,Radix Padoniae Rubra 10g	*Fagopyrum dibotrys* (DDon) Hara [Polygonaceae] 30g,*Astragalus memeranaceus* (Fisch) Bge Var mongholicus (Bge) Hsiao [Fabaceae] 20g,*Belamcanda chinensis* (L) DC [Iridaceae] 15g,*Codonopsis tangshen* Oliv [Campanulaceae] 15g,*Lonicera japonica* Thunb [Caprifoliaceae] 15g,*Ligusticum chuanxiong* Hort [Apiaceae] 15g,*Pseudostellaria heterohylla* (Miq) pax ex pax et Hoffm [Caryophyllaceae] 15g,*Scutellaria baicalensis* Ceorgi [Labiatae] 15g,*Prunus armeniaca* Lvaransu Maxim [Rosaceae] 12g,*Poriacocos* (Schw) Wolf [Polyporaceae] 12g,*FritiLlaria cirrhosa* DDon [Liliaceae] 10g,*Paeonia lactiflora* Pall [Ranunculaceae] 10g
Liang (2019)	Qingwen Jiedu decoction	Fibrosum 30g,Lonicerae Japonicae 15g,Forsythiae Fructus 15g,Fritillariae Thunbergii Bulbus 15g,Coicis Semen 15g,Arctii Fructus 10g,AurantiiFructus 10g,Paridis Rhizoma 10g,Belamcandae Rhizoma 15g,Eriobotryae Folium 10g,Scutellariae Radix 15g,Dioscoreae Rhizoma 10g,Glycyrrhizae radix et rhizoma 5g	CaSO4 2H2O 30g,*Lonicera japonica* Thunb [Caprifoliaceae] 15g,*Forsytniasuspensa* (Thunb) Vahl [Oleaceae Hoffmanns & Link] 15g,*Fritillaria thunbergii* Miq [Liliaceae] 15g,*Coix lacryma jobiLvarmayuen* (Roman) Stapf [Poaceae] 15g,*Arctium lappa* L [Compositae] 10g,*Citrusaurantium* L [Rutaceae] 10g,*Paris polyphylla* Smith varYunnanensis (Franch) Hand-Mazz [Liliaceae] 10g,*Belamcanda chinensis* (L) DC [Iridaceae] 15g,*Eriobotrya japonica* (Thunb)Lindl [Rosaceae] 10g,*Scutellaria baicalensis* Ceorgi [Labiatae] 15g,*Dioscorea opposita* Thunb [Dioscoreaceae] 10g,*Glycyrrhiza uralensis* Fisch [Fabaceae] 5g
Liang (2019)	Qingwen Jiedu decoction	Fibrosum 30g,Lonicerae Japonicae 15g,Forsythiae Fructus 15g,Fritillariae Thunbergii Bulbus 15g,Coicis Semen 15g,Arctii Fructus 10g,AurantiiFructus 10g,Paridis Rhizoma 10g,Belamcandae Rhizoma 10g,Eriobotryae Folium 10g,Scutellariae Radix 10g,Dioscoreae Rhizoma 10g,Glycyrrhizae radix et rhizoma 5g	CaSO4 2H2O 30g,*Lonicera japonica* Thunb [Caprifoliaceae] 15g,*Forsytniasuspensa* (Thunb) Vahl [Oleaceae Hoffmanns & Link] 15g,*Fritillaria thunbergii* Miq [Liliaceae] 15g,*Coix lacryma jobiLvarmayuen* (Roman) Stapf [Poaceae] 15g,*Arctium lappa* L [Compositae] 10g,CitrusaurantiumL [Rutaceae] 10g,*Paris polyphylla* Smith varYunnanensis (Franch) Hand-Mazz [Liliaceae] 10g,*Belamcanda chinensis* (L) DC [Iridaceae] 10g,*Eriobotrya japonica* (Thunb) Lindl [Rosaceae] 10g,*Scutellaria baicalensis* Ceorgi [Labiatae] 10g,*Dioscorea opposita* Thunb [Dioscoreaceae] 10g,*Glycyrrhiza uralensis* Fisch [Fabaceae] 5g
Liu (2016)	Modified Xiao Qinglong decoction	Pinelliae rhizoma 25g,Zingiberis Rhizoma 15g,Schisandrae chinensis fructus 15g,Cinnamomi Ramulus 15g,Paeoniae Radix Alba 30g,Poria 20g,Atractylodes Macrocephala 30g,Glycyrrhizae radix et rhizoma 10g	*Pinellia ternata* (Thunb) Breit [*Araceae*] 25g,*Zingiber officinale Rosc* [*Zingiberaceae*] 15g,*Schisandra chinensis* (Turcz) Baill [*Magnoliaceae*] 15g,*Cinnamomum cassia Presl* [Lauraceae] 15g,*Paeonia lacD13:D17tiflora Pall* [*Ranunculaceae*] 30g,*Poriacocos* (Schw) Wolf [*Polyporaceae*] 20g,*Atractylodes macrocephala Koidz* [*Compositae*] 30g,*Glycyrrhiza uralensis* Fisch [Fabaceae] 10g
Liu (2019)	Hongteng Zijin decoction	Sargentodoxae caulis 30g,Violae Herba 30g,Lonicerae Japonicae 15g,Forsythiae Fructus 15g,Moutan Cortex 15g,Pinelliae rhizoma 12g,Trichosanthis Fructus 15g,Fritillariae Thunbergii Bulbus 12g,Rhei Radix Et Rhizoma 15g,Herba Patrinine 30g,Coicis Semen 30g,Radix Padoniae Rubra 15g,Rehmannia Glutinosa Libosch 10g,Bupleuri Radix 10g,Scutellariae Radix 15g,Glycyrrhizae radix et rhizoma 5g	*Sargentodoxa cuneata* (Oliv) Rehd et Wils [Lardizabalaceae] 30g,*Viola philipica* Cav [Vconfusa Champ [Violaceae]) 30g,*Lonicera japonica* Thunb [Caprifoliaceae] 15g,*Forsytniasuspensa* (Thunb) Vahl [Oleaceae Hoffmanns & Link] 15g,*Paeonia suffruticosa* Andr [Ranunculaceae] 15g,*Pinellia ternata* (Thunb) Breit [Araceae] 12g,*Trichosanthes kirilowii* Maxim [Cucurbitaceae] 15g,*Fritillaria thunbergii* Miq [Liliaceae] 12g,*Rheum officinale* Bail [Polygonaceae] 15g,*Patrinia scabiosaefolia* Fisch ex Link [Valerianaceae] 30g,*Coix lacryma jobiLvarmayuen* (Roman) Stapf [Poaceae] 30g,*Paeonia lactiflora* Pall [Ranunculaceae] 15g,*Rehmannia glutinosa* Libosch [Scrophulariaceae]10g,*Bupleurum chinensie* DC [Campanulaceae] 10g,*Scutellaria baicalensis* Ceorgi [Labiatae] 15g,*Glycyrrhiza uralensis* Fisch [Fabaceae] 5g
Peng (2019)	Buzhong Yiqi decoction	Bupleuri Radix 8g,Cimicifugae Rhizoma 8g,Angelicae Sinensis Radix 10g,Rhei Radix Et Rhizoma 10g,Glycyrrhizae radix et rhizoma 10g,Codonopsis Radix 15g,Pheretima 15g,Atractylodes Macrocephala 15g,Citri Reticulatae Pericarpium 15g,Citri reticulatae pericarpium viride 15g,Astragali Radix 30g	*Bupleurum chinensie* DC [Campanulaceae] 8g,*Cimicifuga heracleifolia* Kom [Ranunculaceae] 8g,*Angelica sinensis* (Oliv) Diels [Apiaceae] 10g,*Rheum officinale* Bail1 [Polygonaceae] 10g,*Glycyrrhiza uralensis* Fisch [Fabaceae] 10g,*Codonopsis tangshen* Oliv [Campanulaceae] 15g,*Pheretima aspergillum* (E Perrier) [Megascolecidae] 15g,*Atractylodes macrocephala* Koidz [Compositae] 15g,*Citrus reticulata* Blanco [Rutaceae] 15g,*Citri reticulatae pericarpium viride* [Rutaceae] 15g,*Astragalus memeranaceus* (Fisch) Bge Var mongholicus (Bge) Hsiao [Fabaceae] 30g
Qi (2015)	Tongfu Xiefei decoction	Rhei Radix Et Rhizoma 10g,Natrii Sulfas,FructusImmaturus 10g,Magnoliae Officinalis Corte×10g,Atractylodes Macrocephala 30g,Salviae miltiorrhizae radix et rhizoma 15g	*Rheum officinale* Bail1 [Polygonaceae] 10g,Na2SO4•Og,2O,*Citrus aurantium* L [Rutaceae] 10g,*Magnolia officinalis* Rehdet Wils [Magnoliaceae] 10g,*Atractylodes macrocephala* Koidz [Compositae] 30g,*Salvia miltiorrhiza* Bge [Labiatae] 15g
Shi (2017)	Modified Baihu decoction	Gypsum Fibrosum 30g,Anemarrhenae Rhizoma 10g,Glycyrrhizae radix et rhizoma 9g,Ginseng Radix Et Rhizoma 5g,Poria 10g,Macrocephala 10g,Salviae miltiorrhizae radix et rhizoma 10g	CaSO4 2H2O 30g,*Anemarrhena asphodeloides* Bge [Liliaceae] 10g,*Glycyrrhiza uralensis* Fisch [Fabaceae] 9g,*Panax ginseng* CAMey [Araliaceae] 5g,*Poriacocos* (Schw) Wolf [Polyporaceae] 10g,*Atractylodes macrocephala* Koidz [Compositae] 10g,*Salvia miltiorrhiza* Bge [Labiatae] 10g
Tan (2019)	Modified Qingfei Huatan decoction	Gardeniae Fructus,Anemarrhenae Rhizoma,Radix Ophiopogonis,Platycodonis Radix,Citri exocarpium rubrum,Glycyrrhizae radix et rhizoma,Fritillariae Thunbergii Bulbus,Mori Cortex,Poria,Trichosanthis semen	*Cardenia jasminoides* Ellis [Rubiaceae],*Anemarrhena asphodeloides* Bge [Liliaceae],*Ophiopogon japonicus* (Lf) Ker-Gawl [Liliaceae],*Platycodon grandiflorus* (Jacq) A DC [Campanulaceae],Citrus reticulata Blanco [Rutaceae],*Glycyrrhiza uralensis* Fisch [Fabaceae],*Fritillaria thunbergii* Miq [Liliaceae],*Morus alba* L [Moraceae],*Poriacocos* (Schw) Wolf [Polyporaceae],*Trichosanthes kirilowii* Maxim [Cucurbitaceae]
Tang (2016)	Modified Liu Junzi decoction	Ginseng Root and Rhizome 30g,Macrocephala 30g,Poria 30g,Citri Reticulatae Pericarpium 15g,Pinelliae rhizoma 12g,Ephedrae Herba 10g,Armeniacae semen amarum 10g,Glycyrrhizae radix et rhizoma 15g,Trichosanthis fructus 30g,Allii Macrostemonis Bulbus 15g,Platycodonis Radix 30g	*Panaxginseng* CAMey 30g,*Atractylodes macrocephala* Koidz [Compositae] 30g,*Poriacocos* (Schw) Wolf [Polyporaceae] 30g,*Citrus reticulata* Blanco [Rutaceae] 15g,*Pinellia ternata* (Thunb) Breit [Araceae] 12g,*Ephedra sinica* Stapf [Ephedraceae] 10g,*Prunus armeniaca* Lvaransu Maxim [Rosaceae] 10g,*Glycyrrhiza uralensis* Fisch [Fabaceae] 15g,*Trichosanthes kirilowii* Maxim [Cucurbitaceae] 30g,*Allii Allium macrostemon* Bge [Liliaceae] 15g,*Platycodon grandiflorus* (Jacq) A DC [Campanulaceae] 30g
Wang (2019)	TCM formula for individual research	Codonopsis Radix 15g,Poria 12g,Macrocephala 15g,Glycyrrhizae radix et rhizoma 6g,Pinelliae rhizoma 15g,Citri exocarpium rubrum 10g,Scutellariae Radix 10g,Trichosanthis fructus 30g,Gardeniae Fructus 10g,Anemarrhenae Rhizoma 10g,Fritillariae Thunbergii Bulbus 10g,Mori Cortex 30g,Houttuyniae Herba 30g,Lonicerae Japonicae 30g	*Codonopsis tangshen* Oliv [Campanulaceae] 15g,*Poriacocos* (Schw) Wolf [Polyporaceae] 12g,*Atractylodes macrocephala* Koidz [Compositae] 15g,*Glycyrrhiza uralensis* Fisch [Fabaceae] 6g,*Pinellia ternata* (Thunb) Breit [Araceae] 15g,*Citrus reticulata* Blanco [Rutaceae] 10g,*Scutellaria baicalensis* Ceorgi [Labiatae] 10g,*Trichosanthes kirilowii* Maxim [Cucurbitaceae] 30g,*Cardenia jasminoides* Ellis [Rubiaceae] 10g,*Anemarrhena asphodeloides* Bge [Liliaceae] 10g,*Fritillaria thunbergii* Miq [Liliaceae] 10g,*Morus alba* L [Moraceae] 30g,*Houttuynia cordata thunb* [Saururaceae] 30g,*Lonicera japonica* Thunb [Caprifoliaceae] 30g
Wang (2020)	Mahuang Shengma decoction	Ephedrae Herba 12g,Cimicifugae Rhizoma 6g,Cinnamomi Ramulus 15g,Polygonati Odorati Rhizoma 15g,Scutellariae Radix 9g,Anemarrhenae Rhizoma 9g,Angelicae Sinensis Radix 9g,Asparagi Radix 6g,Paeoniae Radix Alba 6g,Zingiberis Rhizoma 6g,Glycyrrhizae radix et rhizoma 6g	*Ephedra sinica* Stapf [Ephedraceae] 12g,*Cimicifuga heracleifolia* Kom [Ranunculaceae] 6g,*Cinnamomum cassia* Presl [Lauraceae]15g,*Polygonatumodoratum* (Mill) Druce [Liliaceae] 15g,*Scutellaria baicalensis* Ceorgi [Labiatae] 9g,*Anemarrhena asphodeloides* Bge [Liliaceae] 9g,*Angelica sinensis* (Oliv)Diels [Apiaceae] 9g,*Asparaguscochinchinensis* (Lour) Merr [Liliaceae] 6g,*Paeonia lactiflora* Pall [Ranunculaceae] 6g,*Zingiber officinale* Rosc [Zingiberaceae] 6g,*Glycyrrhiza uralensis* Fisch [Fabaceae] 6g
Xiao (2021)	Xiao Chaihu decoction	Bupleuri Radix 25g,Scutellariae Radix 15g,Pinelliae rhizoma 15g,Zingiberis Rhizoma Recens 15g,Jujubae Fructus 15g,Glycyrrhizae radix et rhizoma 15g,Pinelliae rhizoma 15g	*Bupleurum chinensie* DC [Campanulaceae] 25g,*Scutellaria baicalensis* Ceorgi [Labiatae] 15g,*Pinellia ternata* (Thunb) Breit [Araceae] 15g,*Zingiber officinale* Rosc [Zingiberaceae] 15g,*Ziziphus jujuba* Mill [Rhamnaceae] 15g,*Glycyrrhiza uralensis* Fisch [Fabaceae] 15g,*Pinellia ternata* (Thunb) Breit [Araceae] 15g
Xiao (2020)	Qingjin Huatan decoction	Fritillariae Thunbergii Bulbus 15g,Scutellariae Radix 10g,Platycodonis Radix 10g,Gardeniae Fructus 10g,Radix Ophiopogonis 10g,Poria 10g,Mori Cortex 10g,Glycyrrhizae radix et rhizoma 6g	*Fritillaria thunbergii* Miq [Liliaceae] 15g,*Scutellaria baicalensis* Ceorgi [Labiatae] 10g,*Platycodon grandiflorus* (Jacq)A DC [Campanulaceae] 10g,*Cardenia jasminoides* Ellis [Rubiaceae] 10g,*Ophiopogon japonicus* (Lf) Ker-Gawl [Liliaceae] 10g,*Poriacocos* (Schw)Wolf [Polyporaceae] 10g,*Morus alba* L [Moraceae] 10g,*Glycyrrhiza uralensis* Fisch [Fabaceae] 6g
Xie (2017)	Feiyan Heji decoction	Isatidis Radix 30g,Salviae miltiorrhizae radix et rhizoma 20g,Stemonae Radix 15g,Scutellariae Radix 15g,Gardeniae Fructus 10g,Fritillariae Thunbergii Bulbus 15g,Glycyrrhizae radix et rhizoma 10g,Arisaema cum bile 6g,Fritillariae Cirrhosae Bulbus 5g	*Isatis indigotica* Fort [Brassicaceae] 30g,*Salvia miltiorrhiza* Bge [Labiatae] 20g,*Stemona ja ponica* (BL) Miq [Stemonaceae] 15g,*Scutellaria baicalensis* Ceorgi [Labiatae] 15g,*Cardenia jasminoides* Ellis [Rubiaceae] 10g,*Fritillaria thunbergii* Miq [Liliaceae] 15g,*Glycyrrhiza uralensis* Fisch [Fabaceae] 10g,Dannaxing 6g,*FritiLlaria cirrhosa* DDon [Liliaceae] 5g
Xu (2019)	Modified Mahuang Shengma decoction	Ephedrae Herba 12g,Cimicifugae Rhizoma 6g,Angelicae Sinensis Radix 12g,Anemarrhenae Rhizoma 9g,Scutellariae Radix 9g,Polygonati Odorati Rhizoma 15g,Paeoniae Radix Alba 12g,Asparagi Radix 12g,Cinnamomi Ramulus 6g,Poria 12g,Gypsum Fibrosum 30g,Glycyrrhizae radix et rhizoma 3g,Zingiberis Rhizoma 3g,Armeniacae semen amarum 10g,Fritillariae Cirrhosae Bulbus 10g	*Ephedra sinica* Stapf [Ephedraceae] 12g,*Cimicifuga heracleifolia* Kom [Ranunculaceae] 6g,*Angelica sinensis* (Oliv) Diels [Apiaceae] 12g,*Anemarrhena asphodeloides* Bge [Liliaceae] 9g,*Scutellaria baicalensis* Ceorgi [Labiatae] 9g,*Polygonatumodoratum* (Mill)Druce [Liliaceae] 15g,*Paeonia lactiflora* Pall [Ranunculaceae] 12g,*Asparaguscochinchinensis* (Lour)Merr [Liliaceae] 12g,*Cinnamomum cassia* Presl [Lauraceae] 6g,*Poriacocos* (Schw)Wolf [Polyporaceae] 12g,CaSO4 2H2O 30g,*Glycyrrhiza uralensis* Fisch [Fabaceae] 3g,*Zingiber officinale* Rosc [Zingiberaceae] 3g,*Prunus armeniaca* Lvaransu Maxim [Rosaceae] 10g,*FritiLlaria cirrhosa* DDon [Liliaceae] 10g
Xue (2015)	TCM formula for individual research	Ephedrae Herba 6g,Armeniacae semen amarum 10g,Gypsum Fibrosum 40g,Polygoni cuspidati rhizoma et radix 15g,Lonicerae Japonicae 20g,Isatidis Folium 15g,Bupleuri Radix 15g,Scutellariae Radix 15g,Houttuyniae Herba 20g,Indigo Naturalis 15g,Dryopteridis crassirhizomatis rhizoma 15g,Paridis rhizoma 12g,Pheretima,Bombyx Batryticatus 10g,Chrysanthemi Flos 15g,Glycyrrhizae radix et rhizoma 6g	*Ephedra sinica* Stapf [Ephedraceae] 6g,*Prunus armeniaca* Lvaransu Maxim [Rosaceae] 10g,CaSO4 2H2O 40g,*Polygonum cuspidatum* Sieb et Zucc 15g,*Lonicera japonica* Thunb [Caprifoliaceae] 20g,*Isatis indigotica* Fort [Brassicaceae] 15g,*Bupleurum chinensie* DC [Campanulaceae] 15g,*Scutellaria baicalensis* Ceorgi [Labiatae] 15g,*Houttuynia cordata thunb* [Saururaceae] 20g,*Artemisia annua* L [Compositae] 15g,*Dryopteris crassirhizoma* Nakai [Dryopteridaceae] 15g,*Paris polyphylla* Smith varYunnanensis (Franch) Hand-Mazz [Liliaceae] 12g,*Pheretima aspergillum* (E Perrier) [Megascolecidae],*Bombyx mori linnaeus* [Bombycidae] 10g,*Chrysanthemum morifolium* Ramat [Compositae] 15g,*Glycyrrhiza uralensis* Fisch [Fabaceae] 6g
Yan (2020)	Hongteng Zijin decoction	Violae Herba 30g,Lonicerae Japonicae 15g,Forsythiae Fructus 15g,Moutan Cortex 15g,Pinelliae rhizoma 12g,Trichosanthis Fructus 15g,Fritillariae Thunbergii Bulbus 12g,Rhei Radix Et Rhizoma 10g,Herba Patrinine 30g,Coicis Semen 30g,Radix Padoniae Rubra 15g,Rehmannia Glutinosa Libosch 10g,Bupleuri Radix 10g,Scutellariae Radix 15g,Glycyrrhizae radix et rhizoma 5g	*Viola philipica* CavVconfusa Champ [Violaceae] 30g,*Lonicera japonica* Thunb [Caprifoliaceae] 15g,*Forsytniasuspensa* (Thunb) Vahl [Oleaceae Hoffmanns & Link] 15g,*Paeonia suffruticosa* Andr [Ranunculaceae] 15g, *Pinellia ternata* (Thunb) Breit [Araceae] 12g,*Trichosanthes kirilowii* Maxim [Cucurbitaceae] 15g,*Fritillaria thunbergii* Miq [Liliaceae] 12g,*Rheum officinale* Bail1 [Polygonaceae] 10g,*Patrinia scabiosaefolia* Fisch ex Link [Valerianaceae] 30g,*Coix lacryma jobiLvarmayuen* (Roman) Stapf [Poaceae] 30g,*Paeonia lactiflora* Pall [Ranunculaceae] 15g,*Rehmannia glutinosa* Libosch [Scrophulariaceae]10g,*Bupleurum chinensie* DC [Campanulaceae] 10g,*Scutellaria baicalensis* Ceorgi [Labiatae] 15g,*Glycyrrhiza uralensis* Fisch [Fabaceae] 5g
Yang (2015)	Yiqi Jianpi Huatan decoction	Codonopsis Radix 25g,Macrocephala 10g,Pinelliae rhizoma 10g,Citri Reticulatae Pericarpium 6g,Poria 15g,Aucklandiae Radix 5g,Amomi Fructus 5g,Armeniacae semen amarum 10g,Asari Radix Et Rhizoma Kitag.)3g,Glycyrrhizae radix etSchisandrae chinensis fructus 6g,Zingiberis Rhizoma 10g,Farfarae Flos 10g,Asteris radix et rhizoma 10g,Glycyrrhizae radix et rhizoma 6g	*Codonopsis tangshen* Oliv [Campanulaceae] 25g,*Atractylodes macrocephala* Koidz [Compositae] 10g,*Pinellia ternata* (Thunb) Breit [Araceae] 10g,*Citrus reticulata* Blanco [Rutaceae] 6g,*Poriacocos* (Schw) Wolf [Polyporaceae] 15g,*Aucklandia lappa* Decne [Compositae] 5g,*Amomum villosum* Lour [Zingiberaceae] 5g,*Prunus armeniaca* Lvaransu Maxim [Rosaceae] 10g,*Asarum heterotropoides* Fr Schmidt var mandshurium (Maxim) Kitag [Aristolochiaceae] 3g,*Glycyrrhizae radix* et Schisandra chinensis (Turcz) Baill [Magnoliaceae] 6g,*Zingiber officinale* Rosc [Zingiberaceae] 10g,*Tussilago farfara* L [Compositae] 10g,*Aster tataricus* Lf [Compositae] 10g,*Glycyrrhiza uralensis* Fisch [Fabaceae] 6g
Yao (2021)	Xue Duqing decoction	Rhei Radix Et Rhizoma 10g,Scutellariae Radix 10g,Gardeniae Fructus 10g,Radix Ophiopogonis 10g,Pseudostellariae Radix 30g,Moutan Cortex 30g,Schisandrae chinensis fructus 10g,Hirudo 10g,Glycyrrhizae radix et rhizoma 10g	*Rheum officinale* Bail1 [Polygonaceae] 10g,*Scutellaria baicalensis* Ceorgi [Labiatae] 10g,*Cardenia jasminoides* Ellis [Rubiaceae] 10g,*Ophiopogon japonicus* (Lf) Ker-Gawl [Liliaceae] 10g,*Pseudostellaria heterohylla* (Miq) pax ex pax et Hoffm [Caryophyllaceae] 30g,*Paeonia suffruticosa* Andr [Ranunculaceae] 30g,*Schisandra chinensis* (Turcz)Baill [Magnoliaceae] 10g,*Whitmania* Pigra Whitman 10g,*Glycyrrhiza uralensis* Fisch [Fabaceae] 10g
Zhou (2021)	Tongyang Xiezhuo decoction	Astragali Radix 15g,Pinelliae rhizoma 12g,Trichosanthis fructus 10g,Macrostemonis Bulbus 10g,Cinnamomi Ramulus 10g,Poria 10g,Atractylodes Macrocephala 10g,Rhei Radix Et Rhizoma 10g,Aconiti lateralis radix praeparata 6g,Asari Radix Et Rhizoma Kitag.)3g,Glycyrrhizae radix et rhizoma 6g	*Astragalus memeranaceus* (Fisch) Bge Var mongholicus (Bge) Hsiao [Fabaceae] 15g,*Pinellia ternata* (Thunb) Breit [Araceae] 12g,*Trichosanthes kirilowii* Maxim [Cucurbitaceae] 10g,*Allium macrostemon* Bge [Liliaceae] 10g,*Cinnamomum cassia* Presl [Lauraceae]10g,*Poriacocos* (Schw) Wolf [Polyporaceae] 10g,*Atractylodes macrocephala* Koidz [Compositae] 10g,*Rheum officinale* Bail1 [Polygonaceae] 10g,*Aconitum carmichaelii* Debx [Ranunculaceae] 6g,*Asarum heterotropoides* Fr Schmidt var mandshurium (Maxim) Kitag [Aristolochiaceae] 3g,*Glycyrrhiza uralensis* Fisch [Fabaceae] 6g
Zhu (2016)	Qingfei Tongluo decoction	Astragali Radix 20g,Codonopsis Radix 15g,Pseudostellariae Radix 15g,Poria 12g,Chuanxiong Rhizoma 10g,Radix Padoniae Rubra 10g,Fagopyri dibotryis rhizoma 30g,Scutellariae Radix 15g,Lonicerae Japonicae 15g,Armeniacae semen amarum 12g,Fritillariae Cirrhosae Bulbus 10g,Belamcandae Rhizoma 15g	*Astragalus memeranaceus* (Fisch) Bge Var mongholicus (Bge) Hsiao [Fabaceae] 20g,*Codonopsis tangshen* Oliv [Campanulaceae] 15g,*Pseudostellaria heterohylla* (Miq) pax ex pax et Hoffm [Caryophyllaceae] 15g,*Poriacocos* (Schw) Wolf [Polyporaceae] 12g,*Ligusticum chuanxiong* Hort [Apiaceae] 10g,*Paeonia lactiflora* Pall [Ranunculaceae] 10g,*Fagopyrum* dibotrys (DDon) Hara [Polygonaceae] 30g,*Scutellaria baicalensis* Ceorgi [Labiatae] 15g,*Lonicera japonica* Thunb [Caprifoliaceae] 15g,*Prunus armeniaca* Lvaransu Maxim [Rosaceae] 12g,*FritiLlaria cirrhosa* DDon [Liliaceae] 10g,*Belamcanda chinensis* (L) DC [Iridaceae] 15g
Zhang (2018)	Shashen Maidong decoction	Glehniae radix 15g,Radix Ophiopogonis 20g,Polygonati Odorati Rhizoma 15g,Lablab semen albuM 15g,Trichosanthis Radix 15g,Glycyrrhizae radix et rhizoma 10g,Mori Folium 15g	*Glehnia littoralis* Fr Schmidtex Miq 15g,*Ophiopogon japonicus* (Lf) Ker-Gawl [Liliaceae] 20g,*Polygonatumodoratum* (Mill) Druce [Liliaceae] 15g,*Dolichos lablab* L [Fabaceae] 15g,*Trichosanthes kirilovoii* Maxim [Cucurbitaceae] 15g,*Glycyrrhiza uralensis* Fisch [Fabaceae] 10g,*Morus alba* L [Moraceae] 15g
Zhu (2020)	Xinjia Dayuan granule	Indigo naturalis 6g,Radix Padoniae Rubra 30g,Fibrosum 30g,Tsaoko fructus 10g,Olibanum 10g,Angelicae dahuricae radix 15g,Scutellariae Radix 15g,Bupleuri Radix 15g,Cicadae periostracum 10g,Rhei Radix Et Rhizoma 3g	*Baphicacanthus cusia* (Nees)Bremek [Acanthaceae] 6g,*Paeonia lactiflora* Pall [Ranunculaceae] 30g,CaSO4 2H2O 30g,*Amomum tsao-ko* Crevost et Lemaire [Zingiberaceae] 10g,*Boswellia carterii* Birdw [Burseraceae Kunth] 10g,*Angelica dahurica* (Fischex Hoffm) Benthet Hookf [Apiaceae] 15g,*Scutellaria baicalensis* Ceorgi [Labiatae] 15g,*Bupleurum chinensie* DC [Campanulaceae] 15g,*Cryptotympana pustulata* Fabricius [Cicadidae] 10g,*Rheum officinale* Bail1 [Polygonaceae] 3g
Liang (2016)	Feigan 2 decoction	Scutellariae Radix 15g,Houttuyniae Herba 30g. Lonicerae Japonicae 20g,Forsythiae Fructus 15g,Carthami flos 5g,Persicae semen 15g,Salviae miltiorrhizae radix et rhizoma 15g,Astragali Radix 30g. Atractylodes Macrocephala 15g,Thunbergii Bulbus 10g,Ophiopogonis 15g,Platycodonis Radix 10g,Bubali cornu 30g,Trionycis carapax 30g,Glycyrrhizae radix et rhizoma 6 g	*Scutellaria baicalensis* Ceorgi [Labiatae] 15g,*Houttuynia cordata* thunb [Saururaceae] 30g *Lonicera japonica* Thunb [Caprifoliaceae] 20g,Forsytniasuspensa (Thunb) Vahl [Oleaceae Hoffmanns & Link] 15g,*Carthamus tinctorius* L 5g,*Prunus persica* (L Batsch 15g,Salvia miltiorrhiza Bge [Labiatae] 15g,*Astragalus memeranaceus* (Fisch) Bge Var mongholicus (Bge) Hsiao [Fabaceae] 30g,*Atractylodes macrocephala* Koidz [Compositae] 15g,*Fritillaria thunbergii* Miq [Liliaceae] 10g,*Ophiopogon japonicus* (Lf) Ker-Gawl [Liliaceae] 15g, *Platycodon grandiflorus* (Jacq) A DC [Campanulaceae] 10g,*Bubalus bubalis* Linnaeus [Bovidae] 30g,*Trionyx sinensis* Wiegmann [Trionychidae]30g,*Glycyrrhiza uralensis* Fisch [Fabaceae] 6g
Chen (2022)	Fuzheng Guben decoction	Glycyrrhizae radix et rhizoma 6g,Poria 15g,Scutellariae Radix 15g,Lonicerae Japonicae 15g,Armeniacae semen amarum 15g,Atractylodes Macrocephala 20g,Pseudostellariae Radix 30g,Astragali Radix 30g,Fagopyri dibotryis rhizoma 30g	*Glycyrrhiza uralensis* Fisch [Fabaceae] 6g,Poriacocos (Schw)Wolf [Polyporaceae] 15g,*Scutellaria baicalensis* Ceorgi [Labiatae] 15g,*Lonicera japonica* Thunb [Caprifoliaceae] 15g,*Prunus armeniaca* Lvaransu Maxim [Rosaceae] 15g,*Atractylodes macrocephala* Koidz [Compositae] 20g,*Pseudostellaria heterohylla* (Miq) pax ex pax et Hoffm [Caryophyllaceae] 30g,*Astragalus memeranaceus* (Fisch) Bge Var mongholicus (Bge) Hsiao [Fabaceae] 30g,*Fagopyrum dibotrys* (DDon)Hara [Polygonaceae] 30g
Fang (2022)	Modified Da Chaihu granule	Bupleuri Radix 24g,Scutellariae Radix 15g,Pinelliae rhizoma 9g,Trichosanthis Fructus 15g,Radix Padoniae Rubra 10g,Pseudostellariae Radix 30g,Jujubae Fructus 10g,Glycyrrhizae radix et rhizoma 9g,Zingiberis Rhizoma Recens 10g,Bombyx Batryticatus 12g,Cicadae periostracum 9g,Curcumae longae rhizoma 12g,Indigo Naturalis 15g,Platycodonis Radix 6g,Rhei Radix Et Rhizoma 3g	*Bupleurum chinensie* DC [Campanulaceae] 24g,*Scutellaria baicalensis* Ceorgi [Labiatae] 15g,*Pinellia ternata* (Thunb) Breit [Araceae] 9g,*Trichosanthes kirilowii* Maxim [Cucurbitaceae] 15g,*Paeonia lactiflora* Pall [Ranunculaceae] 10g,*Pseudostellaria heterohylla* (Miq) pax ex pax et Hoffm [Caryophyllaceae] 30g,*Ziziphus jujuba* Mill [Rhamnaceae] 10g,*Glycyrrhiza uralensis* Fisch [Fabaceae] 9g, *Zingiber officinale* Rosc [Zingiberaceae] 10g,*Bombyx mori linnaeus* [Bombycidae] 12g,*Cryptotympana pustulata* Fabricius [Cicadidae]9g,*Curcuma Longa* L [Zingiberaceae]12g,*Artemisia annua* L [Compositae] 15g,*Platycodon grandiflorus* (Jacq) A DC [Campanulaceae] 6g,*Rheum officinale* Bail1 [Polygonaceae] 3g
Li (2022)	Ma Xingyin decoction	Ephedrae Herba 9g,Armeniacae semen amarum 9g,Glycyrrhizae radix et rhizoma 4g,Fibrosum 15g,Phragmitis rhizoma 12g,Bambusae caulis in taenias 12g,Coicis Semen 12g,Lonicerae Japonicae 10g,Forsythiae Fructus 8g,Menthae haplocalycis herba 5g,Schizonepetae herba 5g,Eriobotryae Folium 4g,Descurainiae semen lepidii semen 4g,Asteris radix et rhizoma 4g	*Ephedra sinica* Stapf [Ephedraceae] 9g,*Prunus armeniaca* Lvaransu Maxim [Rosaceae] 9g,*Glycyrrhiza uralensis* Fisch [Fabaceae] 4g,CaSO4 2H2O 15g,*Phragmites communis* Trin [Poaceae Barnhart]12g,*Bambusa tuldoides* Munro [Poaceae Barnhart],*Coix lacryma jobiLvarmayuen* (Roman)Stapf [Poaceae] 12g,*Lonicera japonica* Thunb [Caprifoliaceae] 10g,*Forsytniasuspensa* (Thunb)Vahl [Oleaceae Hoffmanns & Link] 8g,*Mentha haplocalyx* Briq [Labiatae]5g,*Perilla frutescens* (L) Britt [Labiatae]5g,*Schizonepeta tenuifolia* Briq [Labiatae]5g,*Eriobotrya japonica* (Thunb)Lindl [Rosaceae]4g,*Descurainia sophia* (L) Webb ex Prantl [Brassicaceae] 4g,*Aster tataricus* (Lf) [Compositae] 4g
Wang (2022)	TCM formula for individual research	Bupleuri Radix 30g,Paeoniae Radix Alba 30g,Scutellariae Radix 18g,Arisaema cum bile 12g,Pinelliae rhizoma 12g,Jujubae Fructus 6g,Zingiberis Rhizoma Recens 6g,Glycyrrhizae radix et rhizoma 6g,Rhei Radix Et Rhizoma 15g	*Bupleurum chinensie* DC [Campanulaceae] 30g,*Paeonia lactiflora* Pall [Ranunculaceae] 30g,*Scutellaria baicalensis* Ceorgi [Labiatae] 18g,Dannaxing 12g,*Pinellia ternata* (Thunb) Breit [Araceae] 12g,*Ziziphus jujuba* Mill [Rhamnaceae] 6g,*Zingiber officinale* Rosc [Zingiberaceae] 6g,*Glycyrrhiza uralensis* Fisch [Fabaceae] 6g,*Rheum officinale* Bail1 [Polygonaceae] 15g
Chi (2022)	Yiqi Huoxue Huatan decoction	Astragali Radix 100g,Angelicae Sinensis Radix 20g,Citri exocarpium rubrum 9g,Fritillariae Cirrhosae Bulbus 9g,Trichosanthis semen 15g,Platycodonis Radix 9g,Scutellariae Radix 12g,Gardeniae Fructus 12g,Mori Cortex 15g,Glycyrrhizae radix et rhizoma 3g	*Astragalus memeranaceus* (Fisch) Bge Var mongholicus (Bge)Hsiao [Fabaceae]100g,*Angelica sinensis* (Oliv) Diels [Apiaceae] 20g,*Citrus reticulata* Blanco [Rutaceae] 9g,*FritiLlaria cirrhosa* DDon [Liliaceae] 9g,*Trichosanthes kirilowii* Maxim [Cucurbitaceae] 15g,*Platycodon grandiflorus* (Jacq) A DC [Campanulaceae] 9g,*Scutellaria baicalensis* Ceorgi [Labiatae] 12g,*Cardenia jasminoides* Ellis [Rubiaceae] 12g,Morus alba L [Moraceae] 15g,*Glycyrrhiza uralensis* Fisch [Fabaceae] 3g

**TABLE 5:** Top 10 herbs frequently prescribed for pneumonia caused by MDR or XDR bacteria.

CHC (Scientific name)	Frequency of utilization	Relative frequency (%)
*Glycyrrhiza uralensis* Fisch [Fabaceae]	32	84.21
*Scutellaria baicalensis* Georgi [Labiatae]	24	63.16
*Poriacocos* (Schw) Wolf [Polyporaceae]	15	39.47
*Paeonia lactiflora* Pall [Ranunculaceae]	13	34.21
*Pinellia ternate* (Thunb) Breit [Araceae ]	13	34.21
CaSO_4_ 2H_2_O	11	28.95
*Fritillaria thunbergii* Miq [Liliaceae]	10	26.32
*Lonicera japonica* Thunb [Caprifoliaceae]	10	26.32
*Ophiopoon japonicus* (Lf) Ker-awl [Liliaceae]	10	26.32
*Prunus armeniaca* Lvaransu Maxim [Rosaceae]	10	26.32

**TABLE 6:** Apriori algorithm-based association rules in the meta-analysis of CHC prescribed for pneumonia caused by MDR or XDR bacteria.

No	Associations rules		Support	Confidence	Lift
[1]	{*Poriacocos*(Schw)Wolf[Polyporaceae]}=>{*Glycyrrhiza uralensis* Fisch[Fabaceae]}		0.342	0.867	1.029
[2]	{*Pinellia ternata*(Thunb) Breit[Araceae ]}=>{*Glycyrrhiza uralensis* Fisch[Fabaceae]}		0.316	0.923	1.096
[3]	{*Paeonia lactiflora* Pall[Ranunculaceae]}=>{*Scutellaria baicalensis* Georgi[Labiatae]}		0.289	0.846	1.340
[4]	{*Ophiopoon japonicus*(Lf)Ker-awl[Liliaceae]}=>{*Glycyrrhiza uralensis* Fisch[Fabaceae]}		0.263	1.000	1.188
[5]	{*Fritillaria thunbergii* Miq[Liliaceae]}=>{*Glycyrrhiza uralensis* Fisch[Fabaceae]}		0.263	1.000	1.188
[6]	{CaSO42H2O}=>{*Glycyrrhiza uralensis* Fisch[Fabaceae]}		0.263	0.909	1.080
[7]	{*Morus alba* L[Moraceae]}=> {*Glycyrrhiza uralensis* Fisch[Fabaceae]}		0.237	1.000	1.188
[8]	{*Platycodon randiflorus*(Jacq)A DC[Campanulaceae]}=>{*Glycyrrhiza uralensis* Fisch[Fabaceae]}		0.237	1.000	1.188
[9]	{*Citrus reticulata* Blanco[Rutaceae]}=>{*Glycyrrhiza uralensis* Fisch[Fabaceae]}		0.237	1.000	1.188
[10]	{*Trichosanthes kirilowii* Maxim[Cucurbitaceae]}=>{*Glycyrrhiza uralensis* Fisch[Fabaceae]}		0.237	1.000	1.188
[11]	{*Fritillaria thunbergii* Miq[Liliaceae]}=>{*Scutellaria baicalensis* Georgi[Labiatae]}		0.237	0.900	.425
[12]	{*Lonicera japonica* Thunb[Caprifoliaceae]}=>{*Scutellaria baicalensis* Geri[Labiatae]}		0.237	0.900	1.425
[13]	{*Fritillaria thunbergii* Miq[Liliaceae], *Scutellaria baicalensis* Geri[Labiatae]}=>{*Glycyrrhiza uralensis* Fisch[Fabaceae]}		0.237	1.000	1.188
[14]	{*Fritillaria thunbergii* Miq[Liliaceae], *Glycyrrhiza uralensis* Fisch[Fabaceae]}=> {*Scutellaria baicalensis* Georgi[Labiatae]}		0.237	0.900	1.425
[15]	{*Atractylodes macrocephala* Koidz[Compositae]}=>{*Glycyrrhiza uralensis* Fisch[Fabaceae]}		0.211	0.889	1.056
[16]	{*Ziniber officinale* Rosc[Ziniberaceae]}=>{*Glycyrrhiza uralensis* Fisch[Fabaceae]}		0.211	0.889	1.056
[17]	{*Prunus armeniaca* Lvaransu Maxim[Rosaceae]}=>{*Glycyrrhiza uralensis* Fisch[Fabaceae]}		0.211	0.800	0.950
[18]	{*Lonicera japonica* Thunb[Caprifoliaceae]}=>{*Glycyrrhiza uralensis* Fisch[Fabaceae]}		0.211	0.800	0.950

## 4 Discussion

Traditional Chinese medicine(TCM) has been used to treat infectious diseases for thousands of years. The classic works of TCM, such as the “Inner Canon of Huangdi,” “Treatise on Febrile Disease,” and “Febrile Disease Differentiation,” record the rich theory behind treating infectious diseases and the effective methods of treatment. Recently, antibioticresistance has emerged as a global threat. The incidence of MDR and XDR bacteria increases annually, limiting the efficacy of antibiotic agents for the treatment of severe refractory infections (Ayukekbong et al., 2017). Accumulating evidence from clinical studies indicates that patients with pneumonia caused by MDR or XDR bacteria may achieve better clinical outcomes using CHC.

### 4.1 Summary of the main finding

In this study, 38 RCTs were analyzed, of which 37 compared CHC + antibiotics with the same antibiotics alone, and one study compared CHC alone with antibiotics. We found that the combination of CHC and antibiotics significantly increased the response rate. Sensitivity analysis demonstrated that the results were robust, and there was no evidence of publication bias as assessed by trim-and-fill analysis. TSA further explained that the clinical evidence of the efficacy rate of CHC combination therapy for pneumonia caused by MDR or XDR bacteria was determinate; thus, the analytic result is worth asserting. CHC + antibiotic combination treatment was more effective than antibiotics alone in improving the microbiological eradication rate. Subgroup analysis revealed no statistically significant difference in the CAP subgroups. TSA demonstrated that the results were conclusive and robust.

For inflammatory indicators, the combination treatment led to significant improvements in WBC, CRP, and PCT. We identified high heterogeneity in WBC, PCT, and CRP levels; however, the source of heterogeneity remains unclear. We speculated that large individual differences in WBC, PCT, and CRP levels may account for the high heterogeneity, leading to high variation in baseline levels, as well as the degree of improvement. In addition, inadequate study protocols, such as the lack of blinding and allocation concealment, may have contributed to heterogeneity.

In terms of score systems, we also found the CHC combination therapy group had lower CPIS and APACHE-II scores after treatment than the antibiotics-alone group. The CPIS and APACHE-II score were recommended to predict the clinical outcomes of HAP/VAP by recent guidelines (Torres et al., 2017; Martin-Loeches et al., 2018); Lower scores are associated with better outcomes (Zhou et al., 2015). According to our findings, combining CHC and antibiotics could eradicate MDR/XDR bacteria, reduce inflammatory indicator levels, and lower the CPIS and APACHE-II scores, resulting in better clinical outcomes for patients with pneumonia caused by MDR/XDR bacteria. Our results also indicated that patients receiving CHC combination therapy had a shorter length of hospitalization than those receiving antibiotic therapy alone.

### 4.2 Implications of mechanism research

Evidence from this study suggests that the concomitant use of CHC may be more effective than antibiotics alone in the treatment of pneumonia caused by MDR or XDR bacteria. During the antibiotic resistance crisis, many researchers shifted their attention to TCM and investigated the inhibitory effect of TCM herbs or bioactive phytocompounds on antibiotic resistance (Liu et al., 2020; Mohamed et al., 2020; Lan et al., 2021). As the main source of TCM herbs, natural products (NP) are characterized by more complex phytochemicals and spatial configurations than synthetic drugs (Koehn and Carter, 2005). Different extracts from the same herbs vary wildly, which may lead to distinct antimicrobial activity against various antibiotic-resistant bacteria. In one study, the high antimicrobial activity of *L. ethanol* extracts from *Hypericum perforatum* Linn against *Lactobacillus plantarum* (*L. plantarum*), *Enterococcus faecalis* (*E. faecalis*), *Streptococcus mutans* (*S. mutans*), and *Streptococcus sobrinus* (*S. sobrinus*)strains was observed. While *S. sobrinus* and *L. plantarum* were highly inhibited by their water extracts, a moderate antibacterial effect was observed in *S. mutans* and *E. faecalis* (Süntar et al., 2015). Antibiotic-resistance mechanisms of TCM herbs may involve the efflux pump system, enzyme activity, inhibition of biofilm (Tsai et al., 2018), drug target changes, permeability of the bacterial cell membrane (Zhao et al., 2019), and other drug-resistant mechanisms (Anand et al., 2020). Baicalin, a bioactive compound derived from *S. baicalensis* Georgi, could hinder the formation of *P. aeruginosa* biofilms by suppressing QS-related genes, including lasI, lasR, rhlI, rhlR, pqsR, and pqsA (Luo et al., 2017). Moreover, *in vivo* experiments demonstrated the bioactive anti-inflammatory effects of this compound in an acute pneumonia rat model induced by MDR *P. aeruginosa* (Lei et al., 2023). Other studie demonstrated that lonicerin inhibited biofilm formation and subsequently ameliorated *P. aeruginosa* infections in A549 cells by directly inhibiting AlgE activity without affecting bacterial viability or AlgE expression (Xu et al., 2019). Licorice extract was found to stimulate excessive intracellular reactive oxygen species production, inducing oxidative stress and generating a significant number of free radicals. This disrupted the cell wall membrane structure and functionality of *Staphylococcus aureus (S. aureus)*. Additionally, post-treatment of *S. aureus* with licorice extract resulted in a notable reduction in the enzymatic activity of two critical cellular metabolic enzymes: succinate dehydrogenase and succinate dehydrogenase ATPase, thereby interfering with the organism’s energy metabolism system (Singh et al., 2015; Zhang et al., 2023). In one study, under the intervention of 1/4 MIC berberine and 1/8 MIC imipenem (Su and Wang, 2018), the MexXY-OprM efflux pump in *P. aeruginosa* was blocked due to the downregulation of MexZ, MexX, MexY, and the outer membrane protein OprM. Chelerythrine, isolated from the root of *Toddalia asiatica* (Linn) Lam, has been reported to damage the bacterial cell membrane and destroy channels across cell membranes, allowing protein leakage from the cell and suppressing protein biosynthesis. Therefore, it has a strong inhibitory effect on *S. aureus* and MRSA and on the beta-lactamases produced by these bacteria (He et al., 2018). Additionally, the synergistic effect of TCM herbs can enhance the sensitivity of antibiotic-resistant bacteria to antibiotics, leading to their use as sensitizers. For example, there is no significant difference in the antibacterial effect of pterostilbene and gentamycin alone. However, when combined, the two compounds completely inhibited bacterial growth and exhibited synergistic antibacterial effects (Lee et al., 2017). Research reported that the antimicrobial effect of mupirocin against strains of *S. aureus* and MRSA was enhanced when combined with piperine isolated from black pepper through the inhibition of ethidium bromide efflux (Mirza et al., 2011).

### 4.3 Limitations

Our study had some limitations. First, the randomization process of most studies was unclear, and blinding and allocation concealment was often lacking, which introduced a high risk of bias and lowered the methodological quality. Furthermore, none of these studies were registered; therefore, a study protocol was not obtained. Second, the sample sizes of all included studies were small, and therefore, the findings of these studies may not be generalizable. Third, some studies lacked comprehensive reports regarding research characteristics, such as pathogens, setting, and infection type, which limited the analyses of heterogeneity and exploration of the dominant population. Furthermore, the included studies lacked long-term outcome evaluation indicators for CHC + antibiotic treatment in drug-resistant bacterial pneumonia, such as potential relapse rates. Fourth, although we utilized meta-regression and subgroup analysis to investigate the sources of heterogeneity in this article, we were unable to identify them. Therefore, in future research, we aim to establish comprehensive protocols that yield more stable and reliable results. Fifth, all participants included in this study were exclusively sourced from China limiting the generalizability of the findings to other populations. We look forward to the emergence of future large-sample RCTs conducted in various regions to enhance the external validity and reliability of research findings.

## 5 Conclusion

Patients with pneumonia caused by MDR and XDR bacteria could benefit from CHC + antibiotic combination therapy, which improved response and microbiological eradication rates, reduced the inflammatory response, reduced the CPIS and APACHE-II score, and shortened the length of hospitalization. *Scutellaria baicalensis* Georgi [Labiatae], *F. thunbergii* Miq [Liliaceae], *G. uralensis* Fisch [Fabaceae], and *L. japonica* Thunb [Caprifoliaceae] were identified as core herbs for the treatment of antibiotic-resistant bacterial pneumonia.

## Data Availability

The original contributions presented in the study are included in the article/Supplementary Material, further inquiries can be directed to the corresponding authors.
