# Supplementary material for: Chinese herbal compound for multidrug-resistant or extensively drug-resistant bacterial pneumonia: a meta-analysis and trial sequential analysis with association rule mining to identify core herb combinations
Source: Front Pharmacol. 2023 Dec 20;14:1282538. doi: 10.3389/fphar.2023.1282538 (PMC10761442; doi:10.3389/fphar.2023.1282538)
Supplement: Supplementary file 1 [file DataSheet1.docx]

**Contents Page**

[Supplementary Material S1. PRISMA 2020 checklist 1](#_Toc14584)

[Supplementary Material S2. Database and Search Strategies 4](#_Toc10831)

[Supplementary Material S3. Literature excluded after reading the full text and reasons 10](#_Toc26298)

[Supplementary Material S4. Subgroup analysis 24](#_Toc29333)

[Supplementary Material S5. Meta-regression analysis 63](#_Toc14901)

[Supplementary Material S6. Sensitivity analysis of TCMOLs + antibiotics versus antibiotics 69](#_Toc2762)

[Supplementary Material S7 Publication Bias 74](#_Toc25201)

[Supplementary Material S8. Quality of Evidence According to Outcome Measures-GRADE 78](#_Toc10621)

**Supplementary Material S1. PRISMA 2020 checklist**

| **Section and Topic** | **Item #** | **Checklist item** | **Location where item is reported** |
| --- | --- | --- | --- |
| **TITLE** | | |  |
| Title | 1 | Identify the report as a systematic review. | P1 |
| **ABSTRACT** | | |  |
| Abstract | 2 | See the PRISMA 2020 for Abstracts checklist. | P2 |
| **INTRODUCTION** | | |  |
| Rationale | 3 | Describe the rationale for the review in the context of existing knowledge. | P1 |
| Objectives | 4 | Provide an explicit statement of the objective(s) or question(s) the review addresses. | P1 |
| **METHODS** | | |  |
| Eligibility criteria | 5 | Specify the inclusion and exclusion criteria for the review and how studies were grouped for the syntheses. | P3 |
| Information sources | 6 | Specify all databases, registers, websites, organisations, reference lists and other sources searched or consulted to identify studies. Specify the date when each source was last searched or consulted. | P3 |
| Search strategy | 7 | Present the full search strategies for all databases, registers and websites, including any filters and limits used. | Supplementary material 2 |
| Selection process | 8 | Specify the methods used to decide whether a study met the inclusion criteria of the review, including how many reviewers screened each record and each report retrieved, whether they worked independently, and if applicable, details of automation tools used in the process. | P3 |
| Data collection process | 9 | Specify the methods used to collect data from reports, including how many reviewers collected data from each report, whether they worked independently, any processes for obtaining or confirming data from study investigators, and if applicable, details of automation tools used in the process. | P3 |
| Data items | 10a | List and define all outcomes for which data were sought. Specify whether all results that were compatible with each outcome domain in each study were sought (e.g. for all measures, time points, analyses), and if not, the methods used to decide which results to collect. | P4 |
|  | 10b | List and define all other variables for which data were sought (e.g. participant and intervention characteristics, funding sources). Describe any assumptions made about any missing or unclear information. | P4 |
| Study risk of bias assessment | 11 | Specify the methods used to assess risk of bias in the included studies, including details of the tool(s) used, how many reviewers assessed each study and whether they worked independently, and if applicable, details of automation tools used in the process. | P4-5 |
| Effect measures | 12 | Specify for each outcome the effect measure(s) (e.g. risk ratio, mean difference) used in the synthesis or presentation of results. | P3 |
| Synthesis methods | 13a | Describe the processes used to decide which studies were eligible for each synthesis (e.g. tabulating the study intervention characteristics and comparing against the planned groups for each synthesis (item #5)). | P3 |
|  | 13b | Describe any methods required to prepare the data for presentation or synthesis, such as handling of missing summary statistics, or data conversions. | P3 |
|  | 13c | Describe any methods used to tabulate or visually display results of individual studies and syntheses. | P3-4 |
|  | 13d | Describe any methods used to synthesize results and provide a rationale for the choice(s). If meta-analysis was performed, describe the model(s), method(s) to identify the presence and extent of statistical heterogeneity, and software package(s) used. | P3-4 |
|  | 13e | Describe any methods used to explore possible causes of heterogeneity among study results (e.g. subgroup analysis, meta-regression). | P3-4 |
|  | 13f | Describe any sensitivity analyses conducted to assess robustness of the synthesized results. | P3-4 |
| Reporting bias assessment | 14 | Describe any methods used to assess risk of bias due to missing results in a synthesis (arising from reporting biases). | P3-4 |
| Certainty assessment | 15 | Describe any methods used to assess certainty (or confidence) in the body of evidence for an outcome. | P3-4 |
| **RESULTS** | | |  |
| Study selection | 16a | Describe the results of the search and selection process, from the number of records identified in the search to the number of studies included in the review, ideally using a flow diagram. | P4, figure 1 |
|  | 16b | Cite studies that might appear to meet the inclusion criteria, but which were excluded, and explain why they were excluded. | P4,Supplementary material 3 |
| Study characteristics | 17 | Cite each included study and present its characteristics. | P4, table 1 |
| Risk of bias in studies | 18 | Present assessments of risk of bias for each included study. | P4-5, figure 2 |
| Results of individual studies | 19 | For all outcomes, present, for each study: (a) summary statistics for each group (where appropriate) and (b) an effect estimate and its precision (e.g. confidence/credible interval), ideally using structured tables or plots. | P5-15 |
| Results of syntheses | 20a | For each synthesis, briefly summarise the characteristics and risk of bias among contributing studies. | P22 |
|  | 20b | Present results of all statistical syntheses conducted. If meta-analysis was done, present for each the summary estimate and its precision (e.g. confidence/credible interval) and measures of statistical heterogeneity. If comparing groups, describe the direction of the effect. | P5-15 |
|  | 20c | Present results of all investigations of possible causes of heterogeneity among study results. | P5-15 |
|  | 20d | Present results of all sensitivity analyses conducted to assess the robustness of the synthesized results. | P5-15 |
| Reporting biases | 21 | Present assessments of risk of bias due to missing results (arising from reporting biases) for each synthesis assessed. | P22 |
| Certainty of evidence | 22 | Present assessments of certainty (or confidence) in the body of evidence for each outcome assessed. | P22 |
| **DISCUSSION** | | |  |
| Discussion | 23a | Provide a general interpretation of the results in the context of other evidence. | P24 |
|  | 23b | Discuss any limitations of the evidence included in the review. | P26 |
|  | 23c | Discuss any limitations of the review processes used. | P26 |
|  | 23d | Discuss implications of the results for practice, policy, and future research. | P26 |
| **OTHER INFORMATION** | | |  |
| Registration and protocol | 24a | Provide registration information for the review, including register name and registration number, or state that the review was not registered. | P3,CRD42023410587 |
|  | 24b | Indicate where the review protocol can be accessed, or state that a protocol was not prepared. | CRD42023410587 |
|  | 24c | Describe and explain any amendments to information provided at registration or in the protocol. | - |
| Support | 25 | Describe sources of financial or non-financial support for the review, and the role of the funders or sponsors in the review. | P26 |
| Competing interests | 26 | Declare any competing interests of review authors. | P26 |

*From:* Page MJ, McKenzie JE, Bossuyt PM, Boutron I, Hoffmann TC, Mulrow CD, et al. The PRISMA 2020 statement: an updated guideline for reporting systematic reviews. BMJ 2021;372:n71. doi: 10.1136/bmj.n71 For more information, visit: http://www.prisma-statement.org/

**Supplementary Material S2. Database and Search Strategies**

Search terms used in each database and the results of the search.

**PubMed**

|  | Searches | Results |
| --- | --- | --- |
| 1 | (("Pneumonia, Bacterial"[Mesh]) OR (Pneumonia, Bacterial[Title/Abstract]) OR (Bacterial Pneumonia[Title/Abstract]) OR (drug-resistant bacterial pneumonia[Title/Abstract]) OR (drug-resistant pneumoni*[Title/Abstract]) OR (Antibiotic-Resistant pneumonia[Title/Abstract]) OR (Antibiotic-Resistant Bacterial Pneumonia[Title/Abstract]) OR (multidrug-Resistant pneumonia[Title/Abstract]) OR (multidrug-Resistant bacterial pneumonia[Title/Abstract]) OR (extensively drug-resistant pneumonia[Title/Abstract])) | 26,365 |
| 2 | (("Medicine, Chinese Traditional"[Mesh]) OR (Medicine, Chinese Traditional[Title/Abstract]) OR (Traditional Chinese Medicine[Title/Abstract]) OR (Chinese Medicine[Title/Abstract]) OR (TCM[Title/Abstract]) OR (Chinese herbal medicine[Title/Abstract]) OR (decoction[Title/Abstract]) OR (formula[Title/Abstract]) OR (Prescription[Title/Abstract]) OR (Chinese patent medicine[Title/Abstract]) OR (Chinese patent drug[Title/Abstract]) OR (Chinese herbal compound prescription[Title/Abstract]) OR (integrated traditional chinese and western medicine[Title/Abstract])) | 256,716 |
| 3 | (("Randomized Controlled Trial" [Publication Type]) OR (Randomized Controlled Trial[Title/Abstract]) OR (randomized controlled trial[Title/Abstract]) OR (randomized[Title/Abstract]) OR (RCT[Title/Abstract]) OR (controlled clinical trial[Title/Abstract]) OR (Randomly[Title/Abstract]) OR (Random*[Title/Abstract])) | 1,537,085 |
| 4 | #1 AND #2 AND #3 | **45** |

**Web of Science**

|  | Searches | Results |
| --- | --- | --- |
| 1 | TS=(Pneumonia, Bacterial OR Bacterial Pneumonia OR drug-resistant bacterial pneumonia OR drug-resistant pneumoni* OR Antibiotic-Resistant pneumonia OR Antibiotic-Resistant Bacterial Pneumonia OR multidrug-Resistant pneumonia OR multidrug-Resistant bacterial pneumonia OR extensively drug-resistant pneumonia) | 25744 |
| 2 | TS=(Medicine, Chinese Traditional OR Medicine, Chinese Traditional OR Traditional Chinese Medicine OR Chinese Medicine OR TCM OR Chinese herbal medicine OR decoction OR formula OR Prescription OR Chinese patent medicine OR Chinese patent drug OR Chinese herbal compound prescription OR integrated traditional chinese and western medicine) | 569176 |
| 3 | TS=(Randomized Controlled Trial OR randomized controlled trial OR randomized OR RCT OR controlled clinical trial OR Randomly OR Random*) | 2186147 |
| 4 | #1 AND #2 AND #3 | **85** |

**EMBASE**

|  | Searches | Results |
| --- | --- | --- |
| 1 | 'antibiotic resistance'/exp | 214414 |
| 2 | 'antibacterial drug resistance' OR 'antibacterial resistance' OR 'antibiotic non-susceptibility' OR 'antimicrobial drug resistance' OR 'antimicrobial resistance' OR 'bacterial drug resistance' OR 'bacterial resistance' OR 'bacterium resistance' OR 'microbial drug resistance' | 65617 |
| 3 | 'multidrug resistance'/exp | 54442 |
| 4 | 'drug resistance, multiple' OR 'drug resistance, multiple, bacterial' OR 'mdr resistance' OR 'multi-drug resistance' OR 'multiple drug resistance' | 10740 |
| 5 | #1 OR #2 OR #3 OR #4 | 277200 |
| 6 | 'bacterial pneumonia'/exp | 37272 |
| 7 | 'pneumonia, bacterial' OR 'secondary bacterial pneumonia' | 573 |
| 8 | 'respiratory tract infection'/exp | 552750 |
| 9 | #6 OR #7 OR #8 | 552849 |
| 10 | ('medicine, chinese traditional':ab,ti OR 'traditional chinese medicine':ab,ti OR 'chinese medicine':ab,ti OR tcm:ab,ti OR 'chinese herbal medicine':ab,ti OR decoction:ab,ti OR formula:ab,ti OR prescription:ab,ti OR 'chinese patent medicine':ab,ti OR 'chinese patent drug':ab,ti OR 'chinese herbal compound prescription':ab,ti OR 'integrated traditional chinese and western medicine':ab,ti) | 391129 |
| 11 | #5 AND #9 AND #10 AND [humans]/lim AND [clinical study]/lim | 443 |

**COCHRANE**

|  | Searches | Results |
| --- | --- | --- |
| 1 | Pneumonia, Bacterial OR Bacterial Pneumonia OR drug-resistant bacterial pneumonia OR drug-resistant pneumoni* OR Antibiotic-Resistant pneumonia OR Antibiotic-Resistant Bacterial Pneumonia OR multidrug-Resistant pneumonia OR multidrug-Resistant bacterial pneumonia OR extensively drug-resistant pneumonia in Title Abstract Keyword AND Medicine, Chinese Traditional OR Medicine, Chinese Traditional OR Traditional Chinese Medicine OR Chinese Medicine OR TCM OR Chinese herbal medicine OR decoction OR formula OR Prescription OR Chinese patent medicine OR Chinese patent drug OR Chinese herbal compound prescription OR integrated traditional chinese and western medicine in Title Abstract Keyword AND Randomized Controlled Trial OR randomized controlled trial OR randomized OR RCT OR controlled clinical trial OR Randomly OR Random* in Title Abstract Keyword | **578** |

**CNKI**

|  | Searches | Results |
| --- | --- | --- |
| 1 | (SU=('耐药'+' MRSA '+'ESBLs'+'β-内酰胺酶'+'碳青霉烯') OR TKA =('耐药'+' MRSA '+'ESBLs'+'β-内酰胺酶'+'碳青霉烯')) AND TKA=('肺炎'+'肺部感染'+'下呼吸道'+‘耐药菌感染’) AND TKA=('中医'+'中西医结合'+'中成药'+’中药’+’汤’+‘方’+’散’+‘丸’+‘膏’+‘胶囊’+‘自拟’+'颗粒') AND TKA=('随机'*'对照') NOT TI=('鼠'+'兔'+'meta'+'针刺'+'针灸') | **177** |

**Wanfang**

|  | Searches | Results |
| --- | --- | --- |
| 1 | (主题:(耐药) or 主题:(MRSA) or 主题:(ESBLs) or 主题:(β-内酰胺酶) or 主题:(碳青霉烯)) and(主题:(耐药菌肺炎) or 主题:(肺炎) or 主题:(肺部感染) or 主题:(下呼吸道) or 主题:(耐药菌感染)) and(主题:(中药) or 主题:(中医) or 主题:(中西医结合) or 主题:(中成药) or 主题:(中药) or 主题:(汤) or 题名:(方) or 主题:(散) or 主题:(丸) or 主题:(膏) or 主题:(胶囊) or 主题:(自拟) or 主题:(颗粒)) and(摘要:(随机) or 摘要:(随机对照)) not (题名:(鼠) or 题名:(兔) or 题名:(针刺) or 题名:(针灸)) | **1017** |

**VIP**

|  | Searches | Results |
| --- | --- | --- |
| 1 | M=(耐药 OR MRSA OR ESBLs OR β-内酰胺酶 OR 碳青霉烯) AND M=(肺炎 OR 肺部感染 OR 下呼吸道 OR 耐药菌感染) AND M=(中医 OR 中西医结合 OR 中成药 OR 中药 OR 汤 OR 方 OR 散 OR 丸 OR 膏 OR 胶囊 OR 自拟 OR 颗粒) AND R=(随机 OR 随机对照 OR RCT) NOT T=(鼠 OR 兔 OR meta OR 针刺 OR 针灸) | **55** |

**SINOMED**

|  | Searches | Results |
| --- | --- | --- |
| 1 | (( "耐药"[核心字段:智能] OR "MRSA"[核心字段:智能] OR "ESBLs"[核心字段:智能] OR "β-内酰胺酶"[核心字段:智能] OR "碳青霉烯"[核心字段:智能]) AND( "肺炎"[核心字段:智能] OR "肺部感染"[核心字段:智能] OR "下呼吸道"[核心字段:智能] OR "耐药菌感染"[核心字段:智能]) AND( "中医"[核心字段:智能] OR "中西医结合"[核心字段:智能] OR "中成药"[核心字段:智能] OR "中药"[核心字段:智能] OR "汤"[核心字段:智能] OR "方"[标题:智能] OR "散"[核心字段:智能] OR "丸"[核心字段:智能] OR "膏"[核心字段:智能] OR "胶囊"[核心字段:智能] OR "自拟"[核心字段:智能] OR "颗粒"[核心字段:智能]) AND( "随机"[摘要:智能] OR "随机对照"[摘要:智能] OR "RCT"[摘要:智能])) NOT( "鼠"[标题:智能] OR "兔"[标题:智能] OR "meta"[标题:智能] OR "针刺"[标题:智能] OR "针灸"[标题:智能]) | **111** |

#

# Supplementary Material S3. Literature excluded after reading the full text and reasons

1. **Protocols:**
2. CHICTR.Treatment of hospital-acquired pneumonia with multi-drug resistant organism by Buzhong Yiqi decoction based on Fuzheng Quxie classical prescription: study protocol for a randomized controlled trial[J]. Trials, 2019, 20(1):1-8.

[2] CHICTR. Taking placebo as the control, to evaluate the randomized, double-blind, double-blind, randomized, double-blind, randomized, double-blind, randomized, double-blind, evaluation of the early intervention of Qingjie Xuantou Feiwei Recipe (Yinhua Pinggan Granules) in the early and mid-stage of community-acquired drug-resistant bacterial pneumonia [Z]. https://trialsearchwhoint/Trial2aspx?TrialID=ChiCTR2100047501. 2021

[3] NCT. TCM Syndrome Differentiation Treatment on Discharged Elderly Patients With CAP [Z]. https://clinicaltrialsgov/show/NCT04702074. 2021

[4] Wei L, Guo Y, Fei Y, et al. A randomized, double-blind, placebo-controlled, multicenter clinical trial for efficacy and safety of traditional Chinese medicine combined with antibiotics in the treatment of bacterial pneumonia in children [J]. Medicine, 2020, 99(50): e23217

**2) The study design was not a randomized controlled trial:**

[1]Liu Q.Q, Sun H.Y, Gao J, Kong L.B Discussion on TCM pathogenesis of drug-resistant bacteria infection and observation of clinical curative effect of traditional Chinese medicine [j] Modern distance education of Chinese traditional medicine, 2010 (17): 08

[2]Dai Z.Y, Huang Y.P, Li W.J, et al. efficacy analysis of Zhisou powder combined with Sanzi Yangqin Decoction in the adjuvant treatment of senile pneumonia [j] China clinical practical medicine, 2010 (5): 2

**3)There are obvious errors:**

[1] Huang F.Gi, Zhang Q.Y, Tan min Study on clinical efficacy of Ditan Decoction on super drug-resistant bacteria [j] Sino foreign medical research, 2015, (3): 145-, 6

[2] Liu Y.L, Wang Y.Y, Li H, Effect of Shashen Maimendong decoction combined with double dose tigecycline on serum T cell subsets in elderly patients with multidrug-resistant ventilator-associated pneumonia [j] Journal of Baotou Medical College, 2019, 35 (1): 96-7

[3] Peng B.X,Clinical observation on 27 cases of encephalopathy complicated with multidrug-resistant pneumonia treated with Xiaoqinglong Decoction [j] Hunan Journal of traditional Chinese medicine, 2017, 33 (6): 57-8

[4] Wang H.Y Clinical study of Shashen Maimendong Decoction in the treatment of elderly patients with multidrug-resistant ventilator-associated pneumonia [j] Journal of traditional Chinese medicine, 2017, 32 (8): 1403-6

[5] Wu C.H, Wu W.T, Lu Y.B, et al. clinical study of Qingjin Huatan Decoction Combined with western medicine in the treatment of multidrug-resistant Acinetobacter baumannii [j] Smart health, 2019, 5 (22): 159-60+68

[6] Xu M.J, Huang R.L, Qiao Q.J, et al. efficacy analysis of Qingjin Huatan Decoction Combined with western medicine in the treatment of multidrug-resistant Acinetobacter baumannii [j] Clinical research of traditional Chinese medicine, 2013, 5 (12)

[7] Zhao J.S Clinical study of Qingfei Tongluo Decoction in the treatment of pneumonia caused by multidrug-resistant bacteria after stroke [j] Chinese community physician, 2017, 33 (22): 106-7

[8] Zhou G.X Clinical study of heat clearing and phlegm resolving therapy in the treatment of lung distension with phlegm heat stagnation and multidrug-resistant bacterial infection [j] Health care guide, 2017, (41): 235

[9] Lin Y.J, Xu H.R, Wang C.X, et al. effect of traditional Chinese medicine for activating blood circulation and removing blood stasis on cytokines in elderly patients with drug-resistant bacterial pneumonia [j] Contemporary medicine, 2011, 17 (25): 11-2

**4)Duplicate literature:**

[1] DUAN J, ZHOU W X, TIAN Y. 50 cases with multidrug resistant bacteria infection of lower respiratory tract treated with integrative Chinese and western medicine [J]. Henan traditional chinese medicine [he nan zhong yi], 2015, 35(4): 882‐3.

**5)Not oral administration or nasal feeding**

[1] Cai L, Xu H.Y, Wang D.G Combination of traditional Chinese and Western medicine in the treatment of Obstetrics and Gynecology β- Clinical observation of lactamase bacterial infection associated pneumonia [j] China primary medicine, 2017, 24 (3): 326-9

[2] Cheng X.J, Lin Chao L,Zhu H.L, et al. clinical study of xiefei Tongfu formula in the intervention of patients with multidrug-resistant Pseudomonas aeruginosa pneumonia in ICU [j] Chinese medical emergency, 2019, 28 (1): 47-50

[3] Shi W, Song H.J Clinical observation of modified Dachaihu Decoction in the treatment of pulmonary multi drug resistant bacterial infection after cerebral hemorrhage [j] Hebei Traditional Chinese medicine, 2019, 41 (9): 1331-6,42

[4] Yang M, Shu C.J, Yu X.Q, et al. effect of Supplementing Qi and nourishing Yin on clinical efficacy, pathogen clearance rate and inflammatory response of patients with drug-resistant pneumonia in intensive care unit [j] Advances in modern biomedicine, 2020, 20 (4): 710-3

[5] Yu G.D, Peng Z.Y, Clinical observation of Baihu plus Renshen Decoction in the treatment of lower respiratory tract multi drug resistant bacterial infection [j] New Chinese medicine, 2012, 44 (5): 31-2

[6] Zhou W.J, Li Y.Q, Xiao L, Clinical observation of Tongfu Qingfei Decoction Combined with western medicine in the treatment of multi drug resistant Pseudomonas aeruginosa associated pneumonia [j] Chinese medical emergency, 2020, 29 (11): 2028-30

[7] SONG Y, YAO C, SHANG H, et al. Intravenous infusion of Chinese medicine xuebijing significantly improved clinical outcome in severe pneumonia patients in a multiple center randomized controlled clinical trials [J]. European respiratory journal, 2016, 48.

[8] SONG Y, YAO C, SHANG H, et al. Intravenous infusion of Chinese medicine Xuebijing for patients with severe pneumonia: a multicenter, randomised, double-blind controlled trial [J]. Lancet, 2017, 390(SPEC. 1): 34

[9] Li R, Clinical study on the treatment of stroke associated pneumonia with the new method of removing blood stasis and generating blood [d]; Guangzhou University of traditional Chinese medicine, 2014

[10] Zhou Y.Z, Clinical observation of Jianpi Yishen Huoxue Huatan Decoction in the treatment of ventilator-associated pneumonia caused by fully drug-resistant bacteria [j] Hebei Traditional Chinese medicine, 2017, 39 (8): 1157-61

**6)Studies without primary outcomes**

[1] Duan S.Q, Ni W.J, Application of Baihu Decoction in multi drug resistant bacteria infection with fever [j] Chinese folk therapy, 2020, 28 (4): 36-7

[2] Gao Y, Liu H, Mu X.J, et al. clinical effect of Fuzheng Huazhuo Decoction in the treatment of elderly patients with drug-resistant bacterial pneumonia with Qi deficiency and phlegm turbidity obstructing lung syndrome [j] Chinese contemporary medicine, 2021, 28 (5): 206-10

[3] Liu C, Ma S.Z, Yuan G, et al. a randomized controlled study of xinjiadayuan powder in the treatment of hospital acquired pneumonia caused by multidrug-resistant bacteria infection [j] Chinese medical emergency, 2021, 30 (10): 1773-6

[4] Sun Y, Wang X.Y, Clinical observation of Maxing Shigan Decoction and Qianjin Weijing Decoction in the treatment of stroke associated pneumonia [j] Shenzhen Journal of integrated traditional Chinese and Western medicine, 2020, 30 (9): 35-7

[5] Zheng W.H, Huang H.B, Clinical study of antibiotics combined with sequential traditional Chinese medicine in the treatment of multi drug resistant Acinetobacter baumannii pneumonia in ICU [j] Zhongwai Zhong Yi, 2016, 35 (23): 159-60,63

[6] Ju L.N, Clinical study of Fuzheng Quxie Decoction in the treatment of pulmonary infection caused by multidrug-resistant bacteria [j] Research on Integrated Traditional Chinese and Western medicine, 2021, 13 (4): 230-3

**7) The pathogen were not MDR or XDR bacterial**

[1] Dong K.D, Dong Mi, Zhang Y.L, et al. clinical analysis and discussion of 109 cases of elderly patients with Gram negative bacilli pneumonia treated with integrated traditional Chinese and Western medicine [z] Proceedings of the 2008 National Symposium on the prevention and treatment of common diseases of the elderly Xining 2008: 144-6

[2] Guo H, Study on the treatment of senile pneumonia with the method of dispersing lung and clearing Qi [d]; Nanjing University of traditional Chinese medicine, 2010

[3] Wang Yan, Yang Xueqing, Chen Xiaoguang Effect of heat clearing and phlegm resolving traditional Chinese medicine on antibiotic resistance in elderly patients with pulmonary infection [j] Chinese medicine, 2011, 06 (7): 865-6

[4] Yu D.X, The effect of Qinghua formula on the condition related indicators of patients with severe pneumonia and the exploration of its inflammatory mechanism [d]; Chinese Academy of traditional Chinese medicine, 2016

[5] RONG-BING W, JUN-MIN L, YU-YONG J, et al. Therapeutic effects of integrated traditional Chinese medicine and western medicine in treating severe acute respiratory syndrome [J]. Chinese journal of integrative medicine, 2003, 9(4): 259‐62.

[6] Xie D, Clinical observation of integrated traditional Chinese and Western medicine in the treatment of infantile Klebsiella pneumoniae pneumonia [d]; Hubei University of traditional Chinese medicine, 2012

[7] Yang Y.H, Clinical efficacy of xiefei Zhike mixture in the treatment of community-acquired pneumonia (wind heat attacking the lung type) [m]

**8)Without etiological examination or drug resistance detection**

[1] Zeng Y.M, Efficacy evaluation of sequential antibiotics combined with Qingjin Huatan Decoction in the treatment of patients with pneumonia [j] Everyone health (mid Edition), 2016, 10 (12): 65

[2] Chen J.Y, Zhu J.L, Chen Y.J, Clinical observation on 184 cases of pneumonia treated with Zhike Dingchuan Decoction [j] Chinese Journal of Pediatrics, 1994, (01): 45

[3] Chen X, Gong L.P, Efficacy and safety evaluation of Xiebai powder combined with Weigan Decoction in the treatment of pneumonia [j] Science and Technology Innovation Herald, 2015, (4): 215

[4] Dong D.W, Clinical observation of Liuhuang Qingfei Decoction in the treatment of wind warm lung heat disease (heat toxin accumulated in the lung, Qi and Yin injury) [d]; Changchun University of traditional Chinese medicine, 2016

[5] Du H.B, Clinical study of guaqin Qingfei Decoction in the treatment of wind warm lung heat (phlegm heat obstructing the lung syndrome) [d]; Changchun University of traditional Chinese medicine, 2009

[6] Hao J, Curative effect of Maxing Shigan Decoction on ventilator-associated pneumonia and serum MPO, TNF- α Research on level changes [d]; Heilongjiang University of traditional Chinese medicine, 2011

[7] He H.G, Observation on the curative effect of Qianjin Weigan decoction combined with Maxing Shigan Decoction in the treatment of phlegm heat obstructing lung type cap [d]; Guangzhou University of traditional Chinese medicine, 2017

[8] He X.Y, Observation on the curative effect of Maxing Shigan decoction combined with antibiotics in the treatment of elderly community-acquired pneumonia [d]; Beijing University of traditional Chinese medicine, 2011

[9] Hou W.X, Observation on the clinical efficacy of modified Erchen decoction combined with conventional western medicine in the treatment of ICU pneumonia [d]; Beijing University of traditional Chinese medicine, 2019

[10] To P, Clinical observation of modified Chaiqin Wendan Decoction in the treatment of community-acquired pneumonia with phlegm heat obstructing the lung syndrome [d]; Hunan University of traditional Chinese medicine, 2021

[11] Jia Z.Y, Observation on the therapeutic effect of Kangai Decoction on 82 cases of AIDS complicated with pulmonary infection [J] Chinese Community Physician, 2014, (26): 89-90

[12] Jiang J, Clinical value of moxifloxacin combined with modified Yinqiao powder in the treatment of hospital acquired pneumonia [D]; Hubei University of Traditional Chinese Medicine, 2010

[13] Li H.L,Observation on the therapeutic effect of Jiawei Liujunzi decoction on elderly community acquired pneumonia [D]; Guangzhou University of Traditional Chinese Medicine, 2012

[14] Li R, Clinical Observation on the Treatment of Elderly Community Acquired Pneumonia (Syndrome of Qi and Yin Deficiency and Residual Heat Not Exhausted) with Qinghao Biejia Tang [D]; Changchun University of Traditional Chinese Medicine, 2008

[15] Li R, Clinical observation on the treatment of community acquired pneumonia (dampness heat accumulation syndrome) with modified Haoqin Qingdan Tang [D]; Guangzhou University of Traditional Chinese Medicine, 2017

[16] Li S.Q, Xue Q, Deng X.Y, et al. Exploration of anti infection strategies for patients with chronic obstructive pulmonary disease group D complicated with severe lower respiratory tract infection [J] Journal of Modern Integrated Traditional Chinese and Western Medicine, 2016, (8): 865-7

[17] Li Y.Q, Clinical observation on the treatment of 60 cases of non disappearance of rales during the recovery period of pediatric pneumonia with modified Shengmai Powder [J] Chinese Journal of Practical Rural Doctors, 2013, (11): 51-3

[18] Lin C.L, Cheng X.J, Li C.Q, et al. Clinical observation of Sangxinglihuang decoction in the treatment of elderly drug-resistant bacterial pneumonia (phlegm heat accumulation in the lung syndrome) in ICU [J] Chinese Traditional Chinese Medicine Emergency, 2021, 30 (12): 2146-8

[19] Liu K.Q, Evaluation of the therapeutic effect of sequential combination of antibiotics and Qingjin Huatan Tang on severe pneumonia (phlegm heat obstructing lung syndrome) [D]; Nanjing University of Traditional Chinese Medicine, 2015

[20] Lv J.Y, Professor Huang Liping's academic experience and clinical research in the treatment of encephalopathy complicated with pulmonary infection [D]; Guangxi University of Traditional Chinese Medicine, 2016

[21] Luo X.Q, Exploring the clinical application of the traditional Chinese medicine method of cultivating soil and generating gold in patients with drug-resistant bacterial pneumonia [D]; Chengdu University of Traditional Chinese Medicine, 2019

[22] Peng X.H, Clinical efficacy study on the treatment of community acquired pneumonia (lung heat syndrome) with the method of promoting lung and clearing heat [D]; Guangzhou University of Traditional Chinese Medicine, 2013

[23]Pu X.M, Observation on the therapeutic effect of Jiawei Liujunzi decoction on the recovery stage of pediatric bacterial pneumonia with lung spleen deficiency type [D]; Shandong University of Traditional Chinese Medicine, 2012

[24] Xie X.J, Zheng Z.F, Wu Xiaolin Pneumonia mixture for the treatment of 164 cases of pediatric pneumonia [J] Journal of Pediatric Pharmacy, 2000, (04): 48

[25] Xu Y.J, Clinical observation on the therapeutic effect of Shiwei Longdanhua granules on children with pneumonia and asthma (wind heat closed lung syndrome) [D]; Shandong University of Traditional Chinese Medicine, 2013

[26] Yang J.H, Sun H.P, Chen Z, et al. Clinical efficacy evaluation of modified Maxing Huayu Tang in the treatment of pediatric pneumonia with phlegm heat closed lung type asthma [J] Chinese Journal of Traditional Chinese Medicine, 2018, 36 (7): 1714-6

[27] Yang L.L, Clinical Observation on the Efficacy of Qingfei Huatan Formula in Treating Pneumonia with Asthma and Phlegm Heat Type (Community Acquired Pneumonia) [D]; Guangzhou University of Traditional Chinese Medicine, 2010

[28] Yang M,H, Zhang L, Liu J.L, et al. Clinical Observation on the Treatment of 30 Cases of Elderly Pneumonia with Self made Shengmai Jujie Tang [J] Yunnan Journal of Traditional Chinese Medicine, 2013, 34 (04): 37-8

[29] Yang Z.R, Clinical Study on the Treatment of Elderly Pneumonia with Yinqiao San and Buzhong Yiqi Tang Modified [J] Traditional Chinese Medicine Research, 2001, 14 (1): 25-6

[30]E.S, Observation on the therapeutic effect of a comprehensive treatment plan combining traditional Chinese and Western medicine on acute stroke complicated with pulmonary infection [D]; Chengdu University of Traditional Chinese Medicine, 2008

[31] Zhang B.T, Clinical treatment effect analysis of diffuse pulmonary infection [J] Massage and Rehabilitation Medicine (Second Edition), 2012, 3 (12): 193-4

[32] Zhang G.J, Li W.M, Jiang N.M, et al. Clinical observation on 50 cases of elderly bacterial pneumonia treated with integrated traditional Chinese and Western medicine [J] World's Latest Medical Information Abstracts (Continuous electronic journal), 2016, 16 (56): 137 -, 44

[33]Zhao H, Clinical Observation on Traditional Chinese Medicine Syndrome Differentiation and Comprehensive Treatment of Ventilator Associated Pneumonia [M]

1. Zhao Y, Wang H, Zhang S.W et al. Clinical Study on the Treatment of Acute Pneumonia with Compound Qingre Granules [Z] Proceedings of the 2006 National Symposium on Innovative Drugs and New Varieties Research and Development Yantai 2006: 363-9

[35] Li J, Yu X, Li S, et al. Randomized controlled multicenter clinical trial for integrated treatment of community-acquired pneumonia based on traditional Chinese medicine syndrome differentiation [J]. Journal of traditional chinese medicine = chung i tsa chih ying wen pan, 2012, 32(4): 554‐60.

[36] Zhang C.J, Wang S.P, Chen H.H, et al. Clinical study of wind-warm and pulmonary heat syndrome treated with integrated traditional Chinese and Western medicine [J]. Zhong xi yi jie he xue bao [Journal of Chinese integrative medicine], 2005, 3(2): 108‐10.

[37]Dong K.D, Zhang Y.L, Clinical analysis and exploration of 50 cases of hospital acquired elderly pneumonia; Proceedings of the 8th National Geriatrics Academic Conference of the Chinese Medical Association, Xiamen, Fujian, China, F, 2007 [C]

[38]Xu H, Li.M, Wang.C, et al. Evaluation on clinical efficacy of Fuzheng Jiedu

Huayu Decoction combined with antibiotics in the treatment of pneumonia in the

elderly - A multi-center, double-blind, parallel, randomized controlled trial [J].

Complementary therapies in medicine, 2018, 37: 127‐32.

1. Di R,Clinical observation on the treatment of community acquired pneumonia (phlegm heat stagnation lung type) with modified Qingjin Huatan Tang [D]; Heilongjiang University of Traditional Chinese Medicine, 2014

**Final Included References**

[1] F H. Clinical observation on the treatment of ESBLs producing Escherichia coli pneumonia in children with integrated traditional Chinese and Western medicine [J]. Hubei Traditional Chinese Medicine, 2006, 28(6): 9-10.

[2] J D, W.X Z, TIANY. Combined Traditional Chinese and Western Medicine for the Treatment of 50 Cases of Multiple Drug Resistant Bacterial Infection in the Lower Respiratory Tract [J]. Henan Traditional Chinese Medicine, 2015, 35(4): 882-3.

[3] P.Q Y, C.J C, X.L B, et al. Clinical observation on the treatment of multi drug resistant Pseudomonas aeruginosa pulmonary infection after cerebral infarction using the method of supplementing qi, strengthening spleen, and resolving phlegm [J]. Chinese Journal of Integrative Medicine on Cardio-Cerebrovascular Disease, 2015, (12): 1444-5.

[4] S.J Q, Y.Y Z, X.L L, et al. Clinical Observation on Tongfu Jiefei Formula in Treating Pan-resistant Acinetobacter baumannii Pneumonia in ICU [J]. Journal of North Pharmacy, 2015, (3): 73-.

[5] Y.H X. Clinical Observation on the Combination of Traditional Chinese Medicine Pneumonia Mixture and Western Medicine in the Treatment of Extensive drug-resistant Acinetobacter baumannii Pulmonary Infection [J]. Journal of Emergency in Traditional Chinese Medicine, 2015, 24(6): 1076-8.

[6] H T. The clinical research about treating the multidrug-resistence of bacterial pneumonia by the reinforce healthy qi and purge toxins [D]; Chengdu University of Traditional Chinese Medicine, 2016.

[7] H.Y L, H.J S. Modified Xiaoqinglong Decoction for the Treatment of 30 Cases of Encephalopathy Complicated with Multidrug-resistant Bacterial Pneumonia [J]. Henan Traditional Chinese Medicine, 2016, 36(5): 759-61.

[8] J.Z L, S L, Z.N X, et al. Two TCM Decoctions in Treatment of Pulmonary Patients with Multi Drug-Ｒesistant Bacteria Infection [J]. Acta Chinese Medicine, 2016, 31(5): 642-5.

[9] Q.X Z, G.Q Z, B.L G, et al. Clinical efficacy of Qingfei Tongluo decoction in the treatment of pneumonia caused by multidrug-resistant bacteria after stroke [J]. Journal of Shanghai University of Traditional Chinese Medicine, 2016, 30(5): 26-9.

[10] J.W S, T Z, H.P Z, et al. The effect of modified Baihu decoction on the treatment of pneumonia caused by multidrug-resistant bacteria in stroke patients [J]. Guangdong Medical Journal, 2017, 38(z2): 148-50.

[11] Q.L H, L C, C.W L. Curative Effect Dbsernation of observation Peitushengjin method Combined Treatment Intervention of Multi Drug Resistant Bacteria of Pulmonary Infection [J]. Journal of Basic Chinese Medicine, 2017, 23(12): 1728-30.

[12] T.Z F. Dachaihu decoction in the treatment of encephalopathy after multiple resistance clinical observation on the Effect of drug bacteria infection on phlegm heat accumulation [D]; Shaanxi University of Traditional Chinese Medicine, 2017.

[13] X.Y X. Clinical Effect of TCM Pneumonia Mixture Combined with Linezolid on MRSA － induced Pneumonia and the Impact on Serum Inflammatory Cytokines Levels [J]. Practical Journal of Cardiac Cerebral Pneumal and Vascular Disease, 2017, 25(7): 96-9.

[14] Y G, J.Q S, B.J L, et al. Clinical observation of seizures combined with tigecycline and imipenem/cilastatin in the treatment of severe pan-re sistant Acinetobacter baumannii pneumonia [J]. Chinese Journal of Clinical Rational Drug Use, 2017, 10(23).

[15] Q Z, H L. Efficacy and Safety of Decoction of Glehnia and Ophiopogon in the Treatment of Ventilator - associated Pneumonia with Multidrug resistance in the Elderly [J]. International Journal of Geriatrics, 2018, 39(2): 77-80,100.

[16] C.Y X, W.X X, C.H W, et al. Observation on the curative effect of minocycline and other combination drugs plus modified ephedra cimicif ugae decoction on multidrug-resistant Acinetobacter baumannii pneumonia [J]. Chinese Journal of New Clinical Medicine, 2019, 12(10): 1079-82.

[17] G.Y H, M Z, J.W L. Clinical efficacy of the Qingfei Tongluo decoction on pneumonia with multidrug-resistant bacteria after stroke [J]. Clinical Journal of Chinese Medicine, 2019, 11(25): 82-4.

[18] H.J T. Clinical Observation on the Treatment of Poststroke Pneumonia Caused by Multiple Drug Resistant Bacteria (Phlegm Heat Stasis in the Lung) with Self developed Qingfei Huatan Formula [J]. Journal of North Pharmacy, 2019, 16(1): 57-8.

[19] H.W L, F.Z T, K L, et al. Effect of Qingwen Jiedu Decoction on Extensive drug-resistant Pseudomonas Aeruginosa Associated Severe Pneumonia [J]. Journal of Emergency in Traditional Chinese Medicine, 2019, 28(1): 44-6,50.

[20] H.W L, K L, G.F C, et al. Effect of Qingwen Jiedu Decoction on ventilator-associated pneumonia of syndrome of phlegm-heat congesting in the lung caused by extensive drug-resistant acinetobacter baumannii [J]. Modern Journal of Integrated Traditional Chinese and Western Medicine, 2019, 28(23): 2509-13.

[21] L L, D.S Y, Z.J Z. Clinical Observation on The Combined Treatment of Ｒed Vine Zijin Decoction and Western Medicines for Extensive drug-resistance of Baumannicobacter Baumannii Pulmonary Infection [J]. Traditional Chinese Medicinal Research, 2019, 32(4): 22-4.

[22] L W. Clinical observation on the treatment of pulmonary infection caused by multidrug-resistant Pseudomonas aeruginosa after cerebral infarction by supplementing qi, invigorating spleen, clearing heat and resolving phlegm in traditional Chinese medicine [J]. Modern Journal of Integrated Traditional Chinese and Western Medicine, 2019, 28(1): 56-9.

[23] M.H P, X.B C, X.M R, et al. Observation on the therapeutic effect of Buzhong Yiqi decoction in the treatment of multidrug-resistant pneumonia after stroke [J]. Journal of Practical Traditional Chinese Medicine, 2019, 35(12): 1457-8.

[24] Z.A H, X.H H, G.Y G. Therapeutic effect of Xia Yu Huang Decoction on multidrug-resistant Pseudomonas aeruginosa infection in lower respiratory tract [J]. Guide of China Medicine, 2019, 17(13): 161-2.

[25] D.S Y, L L, Z.S Y, et al. Clinical Observation on The Combined Treatment of Ｒed Vine Zijin Decoction and Western Medicines for Extensive Drug-resistance of Baumannicobacter Baumannii Pulmonary Infection [J]. Journal of Basic Chinese Medicine, 2020, 26(1): 76-7,84.

[26] L Z. Exploration on the treatment of hospital acquired pneumonia caused by drug-resistant bacteria with Xinjiada Yuansan [D]; Beijing University of Chinese Medicine, 2020.

[27] Y G, H L, X.J M, et al. The Clinical Effects of Fuzheng Huazhuo Decoction Combined with Western Medicine in the Treatment of Senile Drug-resistant Bacterial Pneumonia with Deficiency of Qi, Phlegm and Obstruction of Lung [J]. Chinese Pharmacoeconomics, 2020, 15(1): 104-7.

[28] Y.D W, S.T R. Efficacy Evaluationof Sequential Traditional Chinese Medicine in Adjuvant Treatment of Multidrug－resistant Acinetobacter Baumannii Pneumonia in ICU [J]. Liaoning Journal of Traditional Chinese Medicine, 2020, 47(7): 87-90.

[29] Z X, F C, J X. Clinical Study on Qingjin Huatan Tang in the Treatment of Pulmonary Infection Caused by Multidrug -Resistance Pseudomonas aeruginosa [J]. Journal of Medical Forum, 2020, 41(6): 155-8.

[30] Q.S X, M.Y M, M.H D, et al. Clinical Observation of Xiaochaihu Decoction on Elderly Patients with Pulmonary Extensive Drug-resistant Acinetobacter Baumannii Infection in ICU [J]. Journal of Emergency in Traditional Chinese Medicine, 2021, 30(5): 861-4.

[31] S.Y Y. Clinical Study of Xueduqing,A Traditional Chinese Medicine Compound,in the Treatment of Multidrug-Resistant Pseudomonas Aeruginosa Pneumoniain Elderly Patients with Phlegm-heat Accumulation in Lung [D]; Anhui University of Chinese Medicine, 2021.

[32] Z.W Z, X.X L. Effect of Tongyang Xiezhuo Prescription on Cytokines，Serum Pulmonary Surfactant Protein，Endothelial Function and Immunosuppression in Elderly Patients with Encephalopathy Complicated with Pulmonary Multidrug-Ｒesistant Bacteria Infection [J]. Acta Chinese Medicine, 2021, 36(5): 1078-83.

[33] J.H L, F.Y Z, S.Z L, et al. Efficacy of modified Maxingyin decoction combined with tigecycline in the treatment of XDRAB ventilator-associated pneumonia and its effect on T lymphocyte subsets [J]. China Journal of Modern Medicine, 2022, 32(13): 75-80.

[34] L F, H.QI W, J Y, et al. Study on the Intervention Effect of Qingfei Shengmai Decoction on Multidrug- resistant Acinetobacter baumannii Infection in AECOPD [J]. Traditional Chinese Drug Research & Clinical Pharmacology, 2022, 33(1): 120-5.

[35] N C. Clinical Study on Fuzheng Guben Tang in the Treatment of Multiple Drug Resistance Pneumonia after Stroke [J]. Modern Medicine and Health Research Electronic Journal, 2022, (020): 93-96.

[36] P.F C. The Clinical Research of Yiqi Huoxue Huatan Decoction in Cure Drug-Resistance Acinetobacter Baumann Pneumonia [D]; Shandong University of Traditional Chinese Medicine, 2022.

[37] WANG X L. Therapeutic Value of Modified Dachaihu Decoction in the Treatment of Phlegm-heat Fu-organs after Encephalopathy [J]. Systems Medicine, 2022, 7(003): 45-48.

[38] Y F, J Z, S.L C, et al. Clinical observation on the treatment of Drug-resistant Bacterial Pneumonia with modified Dachaihu Decoction and Shengjiang Powder [J]. Chinese journal of geriatric care, 2022, 20(5): 75-80.

# Supplementary Material S4. Subgroup analysis

# Response Rate for TCM + antibiotics treatment vs. Antibiotics treatment

**A**

**B**

**C**

**D**

**E**

**F**

**Subgroup analysis of Microbiological response for TCM + antibiotics treatment vs. Antibiotics treatment**

**A**

**B**

**C**

**D**

**E**

**F**

**Subgroup analysis of WBC count for TCM + antibiotics treatment vs. Antibiotics treatment**

**A**

**B**

**C**

**D**

**E**

**F**

**Subgroup analysis of PCT level for TCM + antibiotics treatment vs. Antibiotics treatment**

**A**

**B**

**C**

**D**

**E**

**F**

**Subgroup analysis of CRP level for TCM + antibiotics treatment vs. Antibiotics treatment**

**A**

**B**

**C**

**D**

**E**

**F**

**Subgroup analysis of CPIS score for TCM + antibiotics treatment vs. Antibiotics treatment**

**A**

**B**

**C**

**D**

**E**

**F**

**Subgroup analysis of APACHEII score for TCM + antibiotics treatment vs. Antibiotics treatment**

**A**

**B**

**C**

**D**

.

**Subgroup analysis of Length of hospitalization for TCM + antibiotics treatment vs. Antibiotics treatment**

**A**

**B**

**C**

**D**

**E**

**Supplementary Material S5. Meta-regression analysis**

**Meta-regression analysis of WBC count**

**1.The results of meta-regression analysis of WBC count on average age.**

**
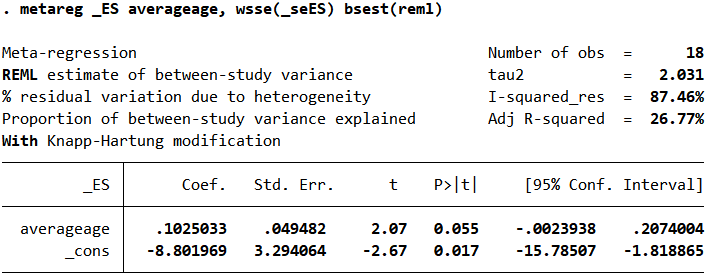
**

**2.The results of meta-regression analysis of WBC count on publication year.**


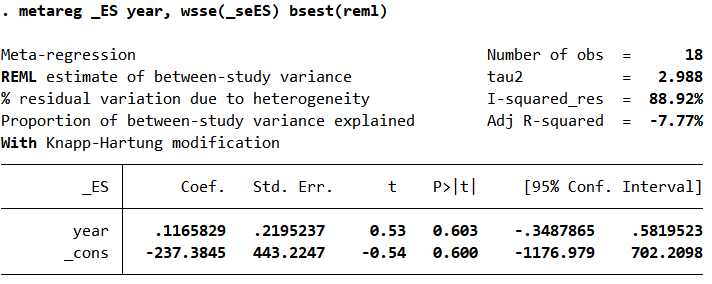


**3.The results of meta-regression analysis of WBC count on sample size.**


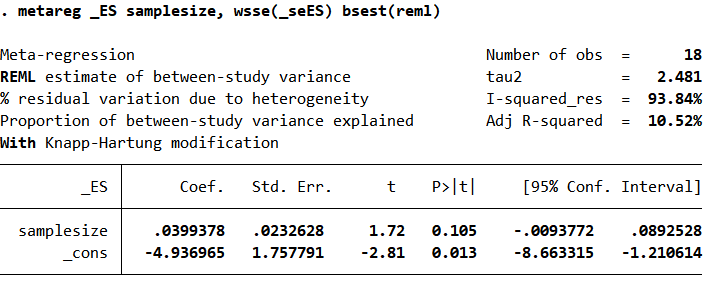


**4.The results of meta-regression analysis of WBC count on treatment duration.**


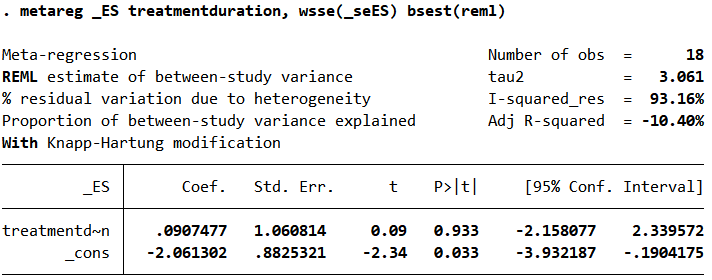


**Meta-regression analysis of PCT level**

**1.The results of meta-regression analysis of PCT level on average age.**

**
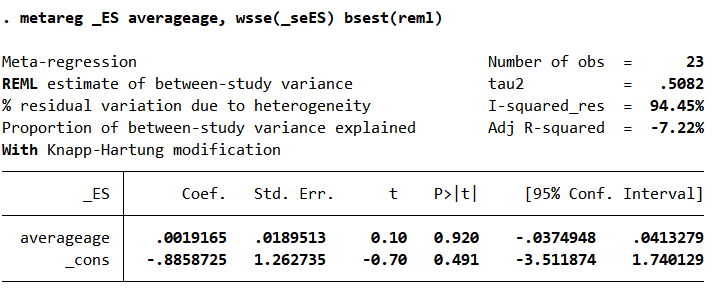
**

**2.The results of meta-regression analysis of PCT level on publication year.**


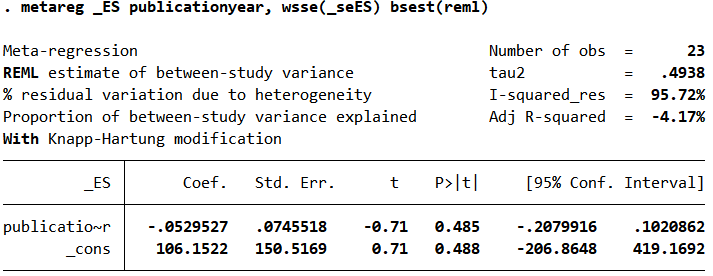


**3.The results of meta-regression analysis of PCT level on sample size.**


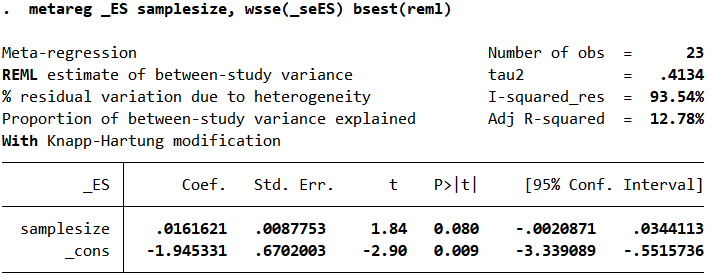


**4.The results of meta-regression analysis of PCT level on treatment duration.**


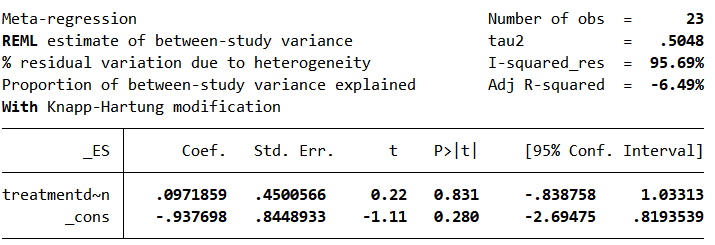


**Meta-regression analysis of CRP level**

**1.The results of meta-regression analysis of CRP level on average age.**

**
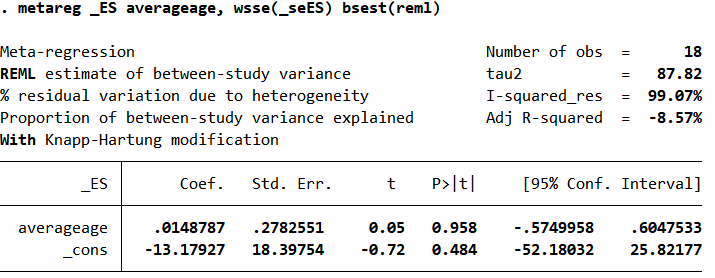
**

**2.The results of meta-regression analysis of CRP level on publication year.**


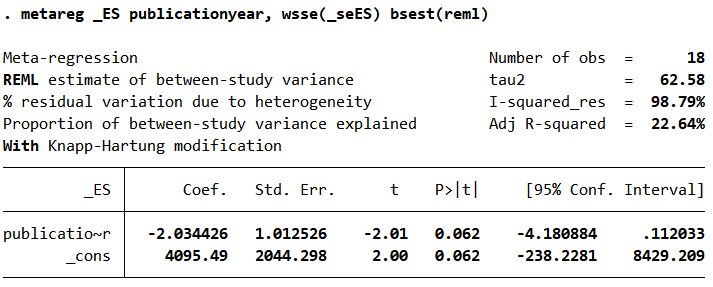


**3.The results of meta-regression analysis of CRP level on sample size.**


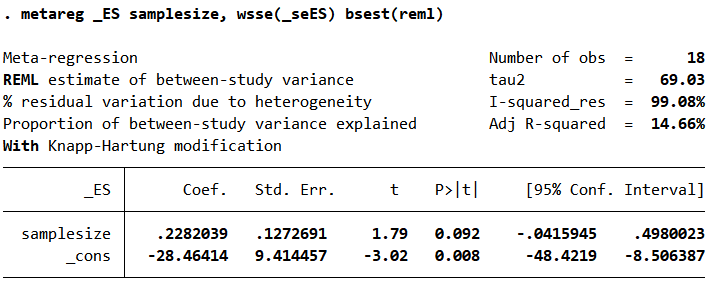


**4.The results of meta-regression analysis of CRP level on treatment duration.**

**
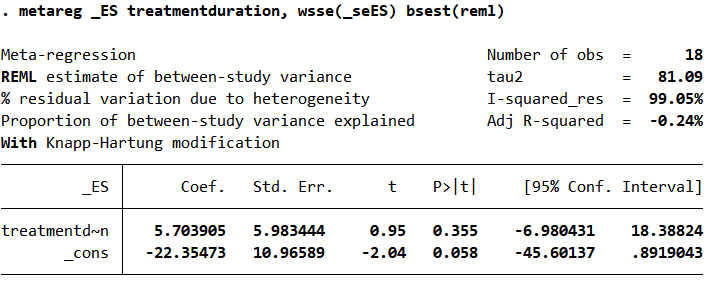
**

**Meta-regression analysis of CPIS score**

**1.The results of meta-regression analysis of CPIS score on average age.**

**
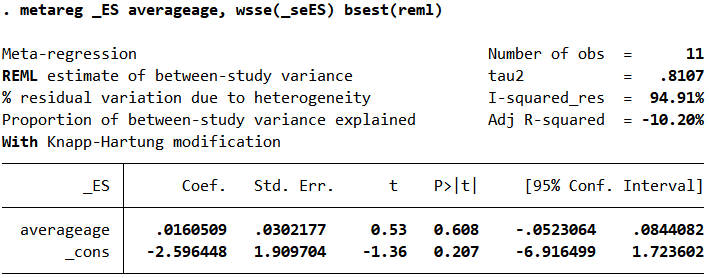
**

**2.The results of meta-regression analysis of CPIS score on publication year.**


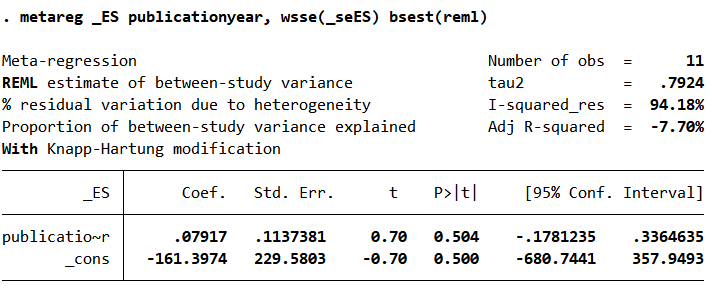


**3.The results of meta-regression analysis of CPIS score on sample size.**


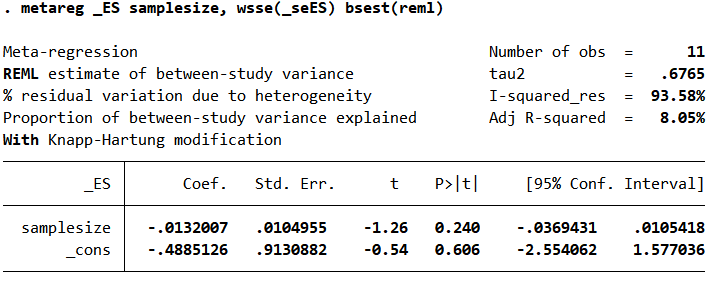


**4.The results of meta-regression analysis of CPIS score on treatment duration.**

**
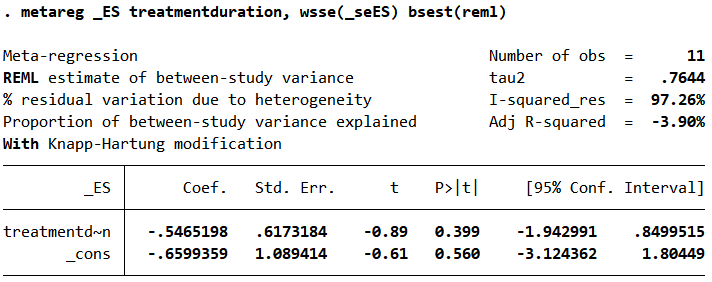
**

**Supplementary Material S6. Sensitivity analysis of TCMOLs + antibiotics versus antibiotics**

**The results of Sensitivity analysis of Response Rate**


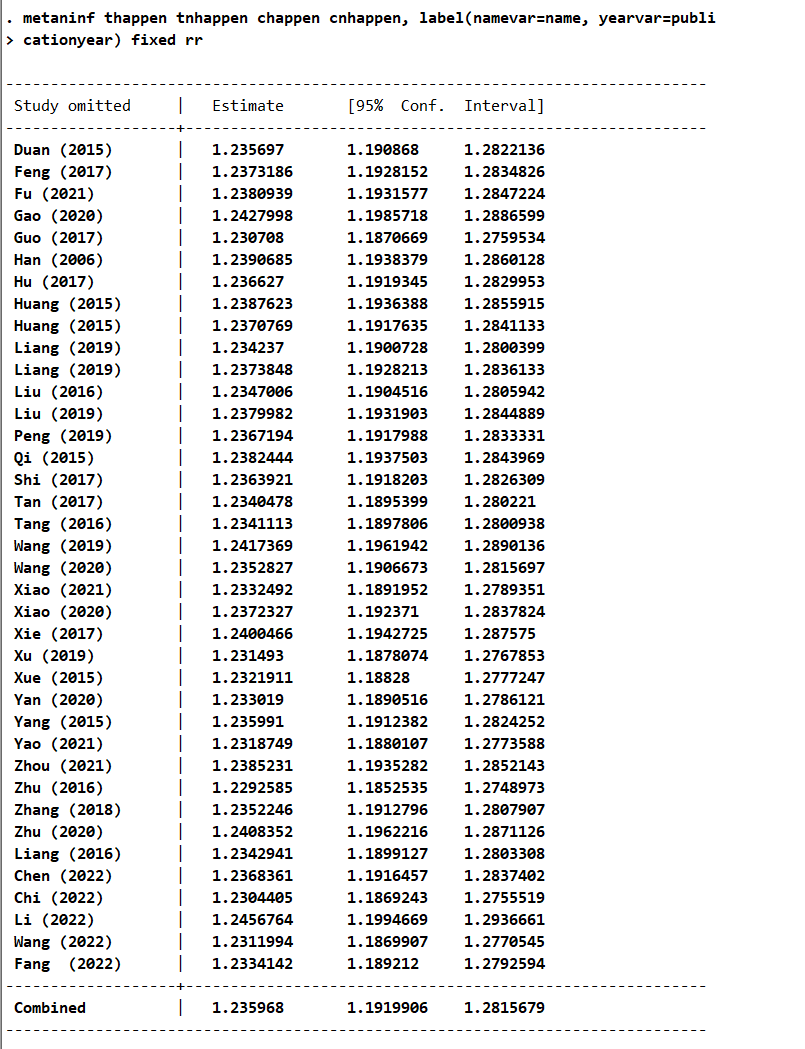


**The results of Sensitivity analysis of Microbiological response**


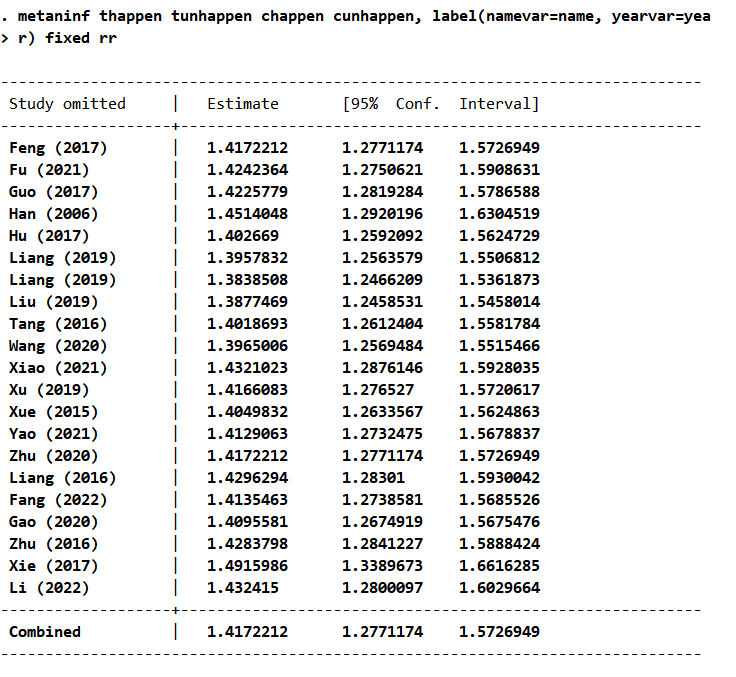


**The results of Sensitivity analysis of WBC count**


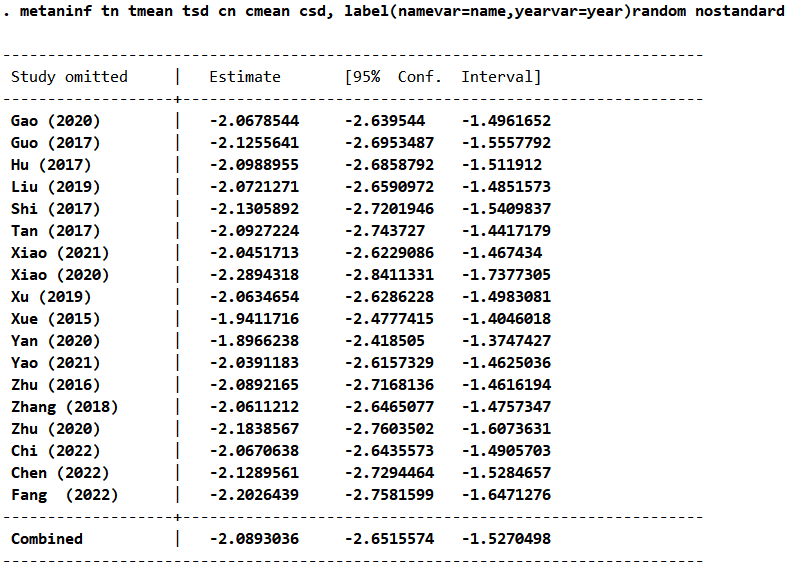


**The results of Sensitivity analysis of PCT level**


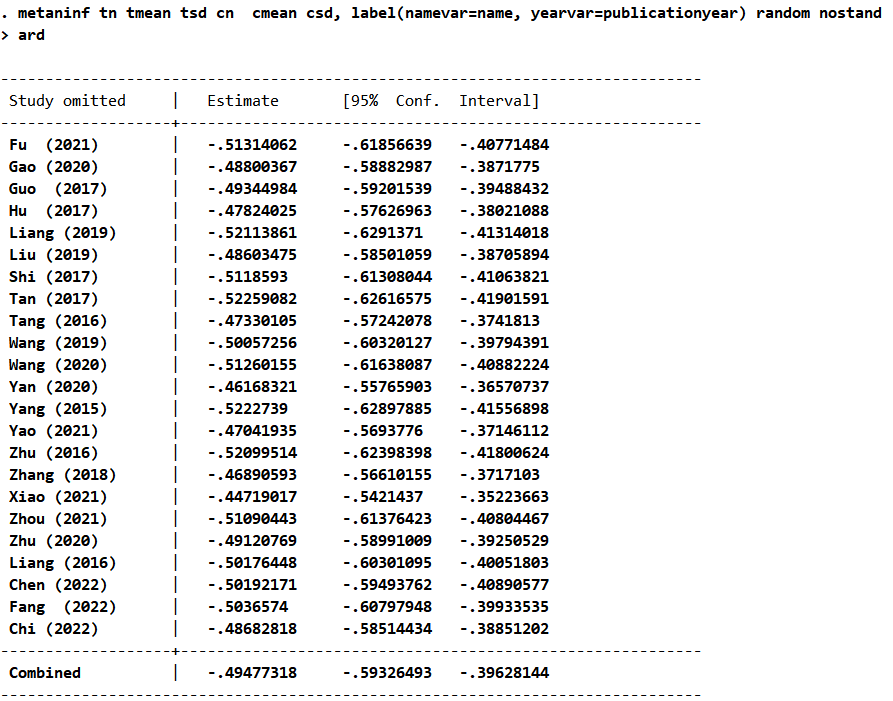


**The results of Sensitivity analysis of CRP level**


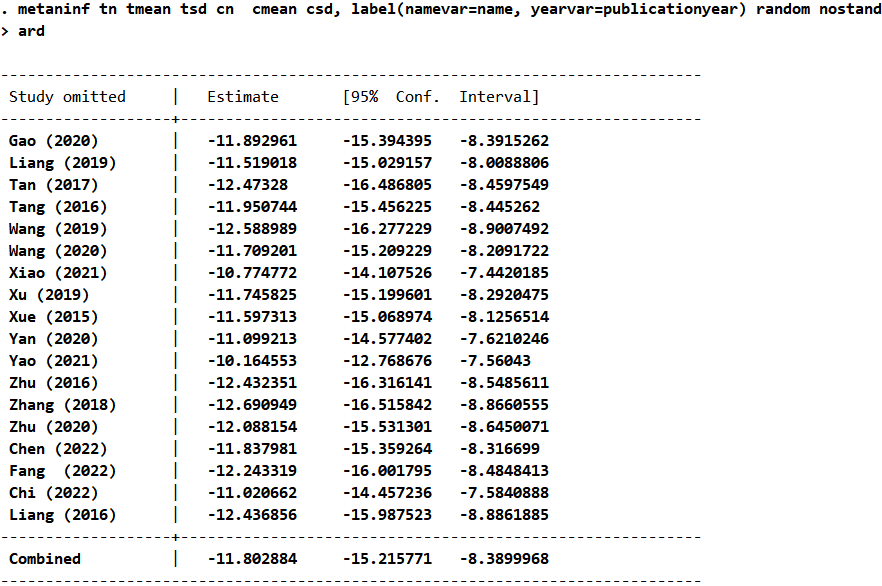


**The results of Sensitivity analysis of CIPS score**

**
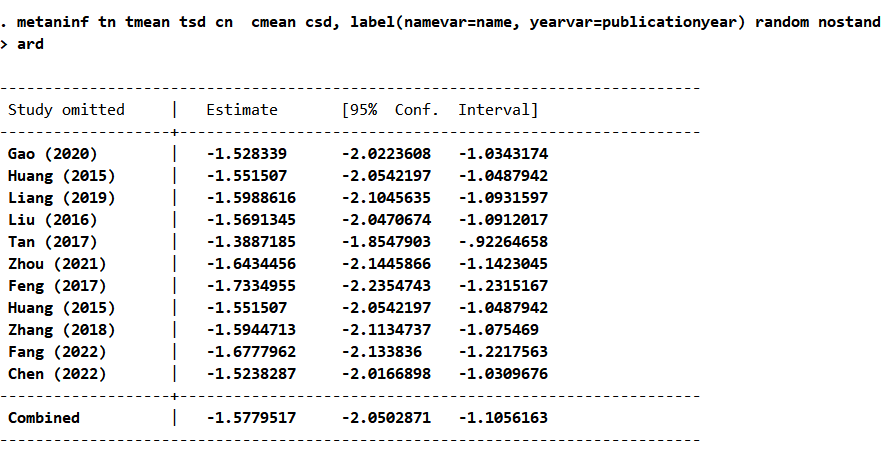
**

**The results of Sensitivity analysis of** **APACHEII score**
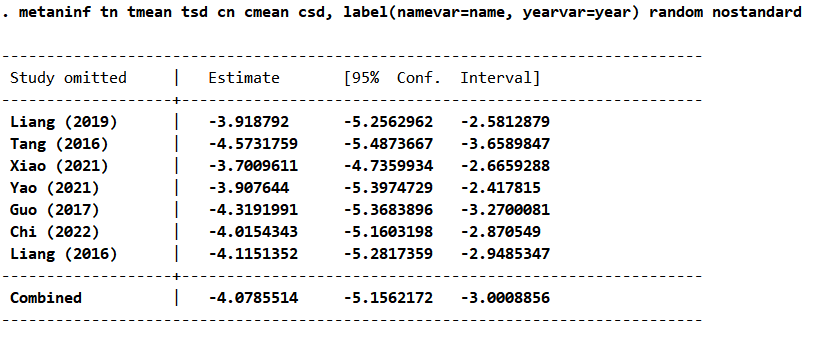


**The results of Sensitivity analysis of** **length of hospitalization**


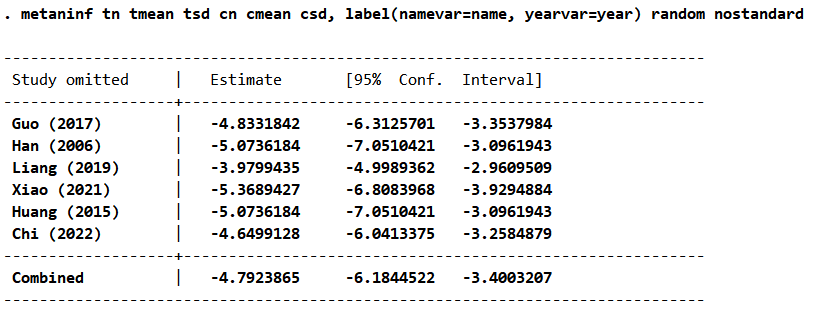


**The results of Sensitivity analysis of safety**


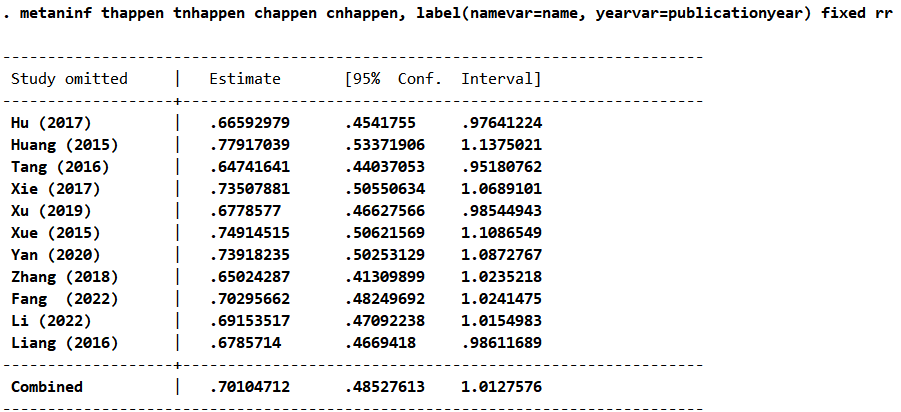


**Supplementary Material S7 Publication Bias**

**The results of Eegger and Begger analysis of Response Rate**


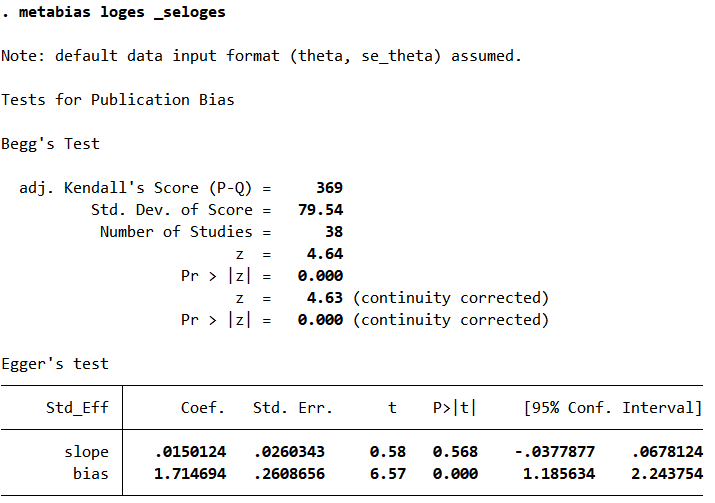


**The results of Trim and Fill analysis of Response Rate**

**
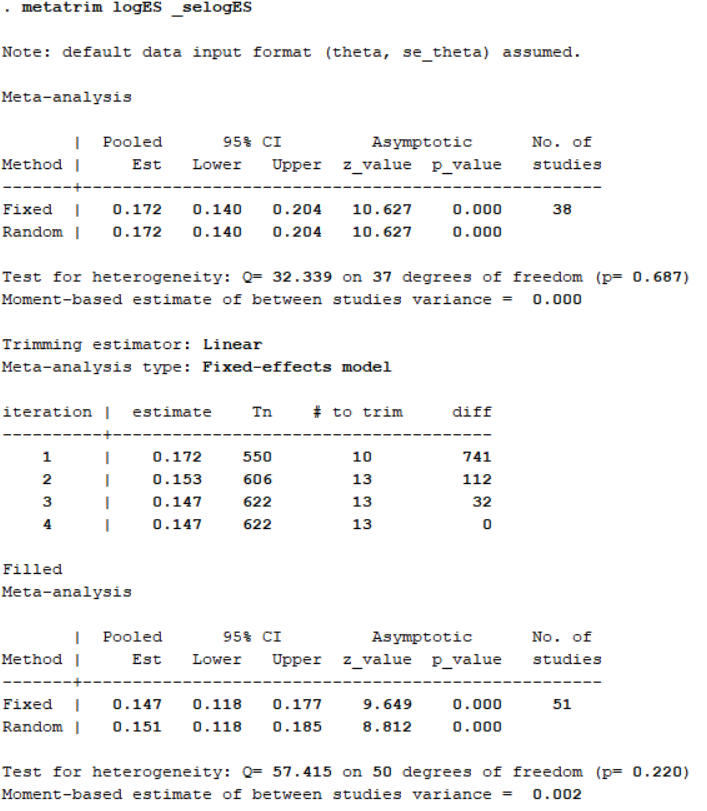
**

**The results of Eegger and Begger analysis of Microbiological response**

**
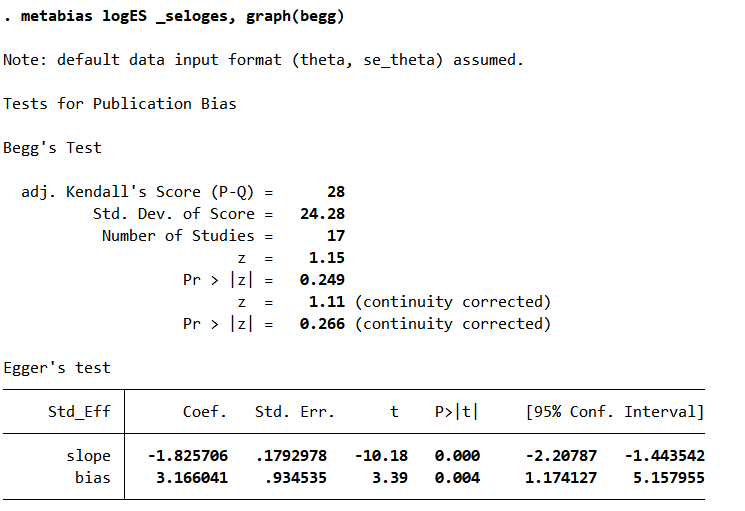
**

**The results of Trim and Fill analysis of Microbiological response**


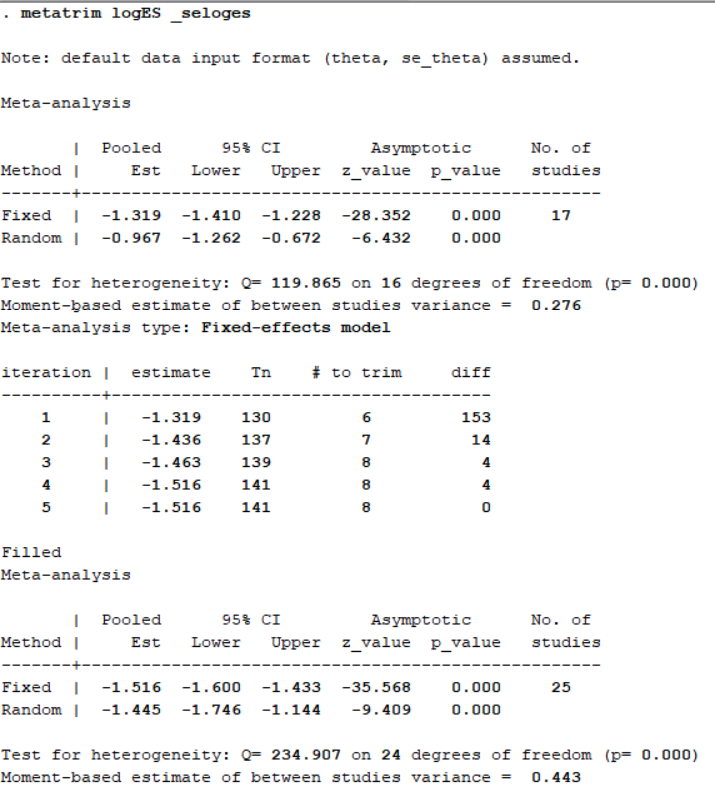


**The results of Eegger and Begger analysis of WBC count**

**
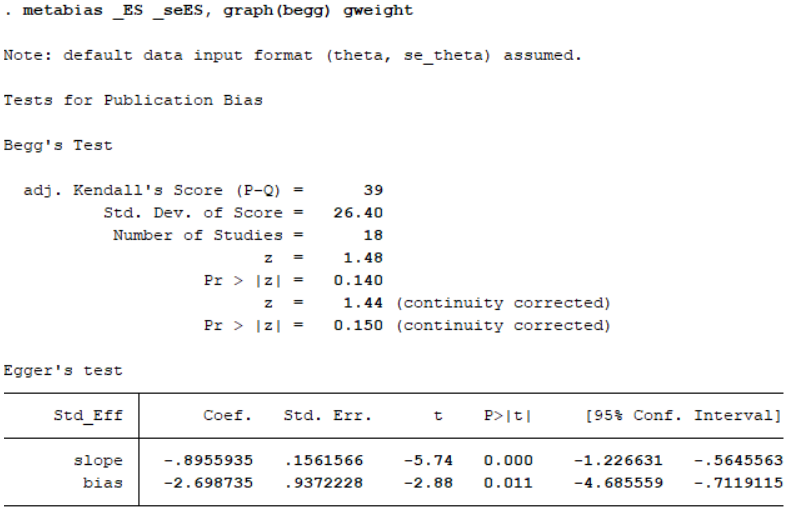
**

**The results of Eegger and Begger analysis of PCT level**


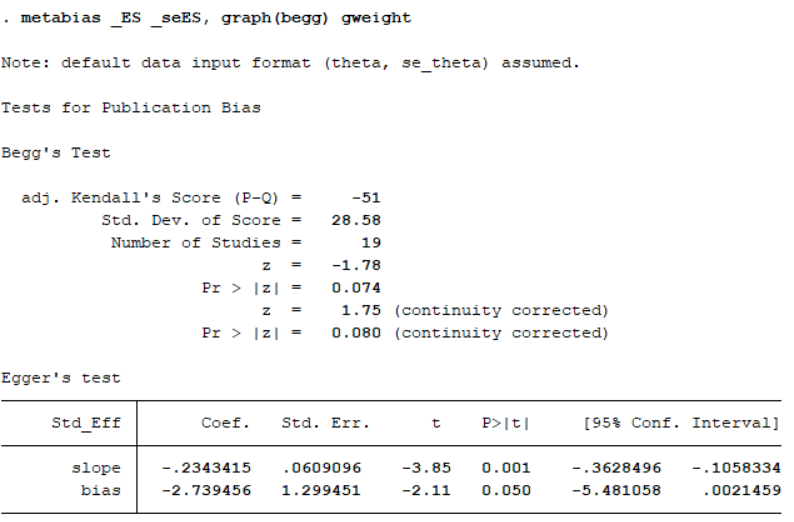


**The results of Eegger and Begger analysis of CRP level**


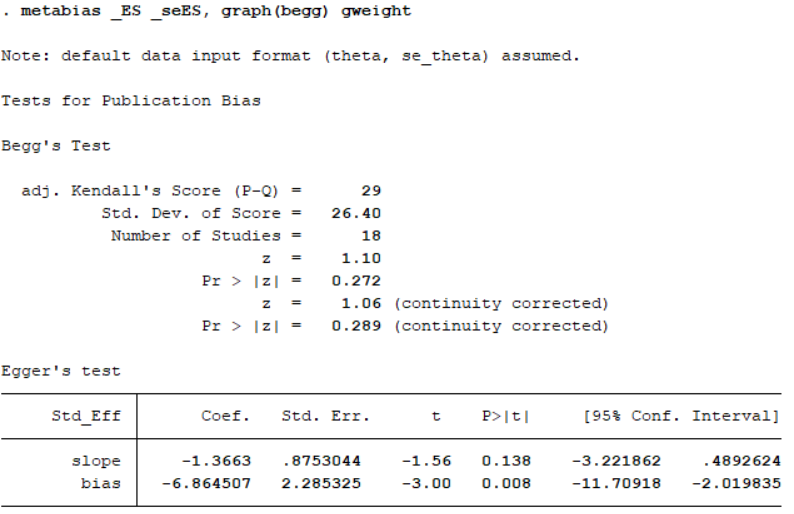


**The results of Eegger and Begger analysis of CIPS score**

**
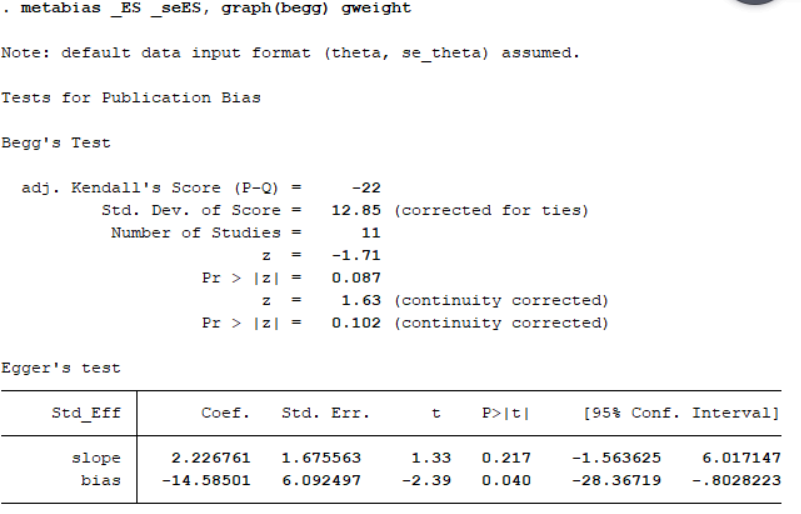
**

**The results of Eegger and Begger analysis of APACHEII score**


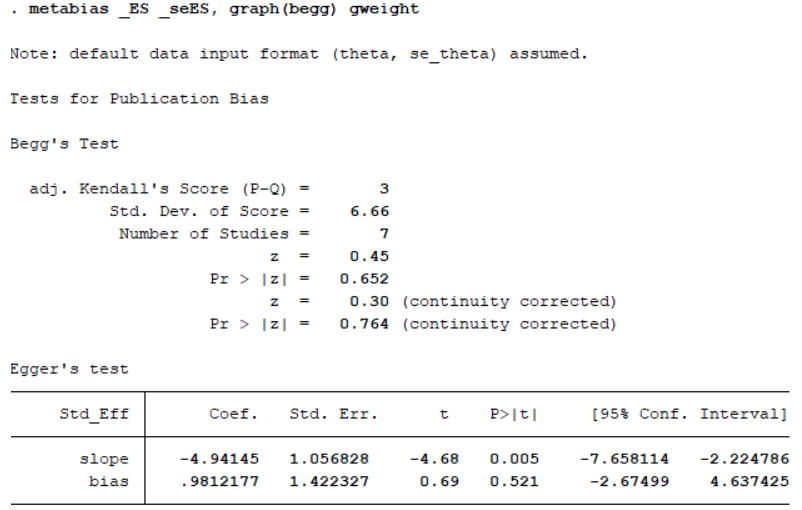


**The results of Eegger and Begger analysis of length of hospitalization**


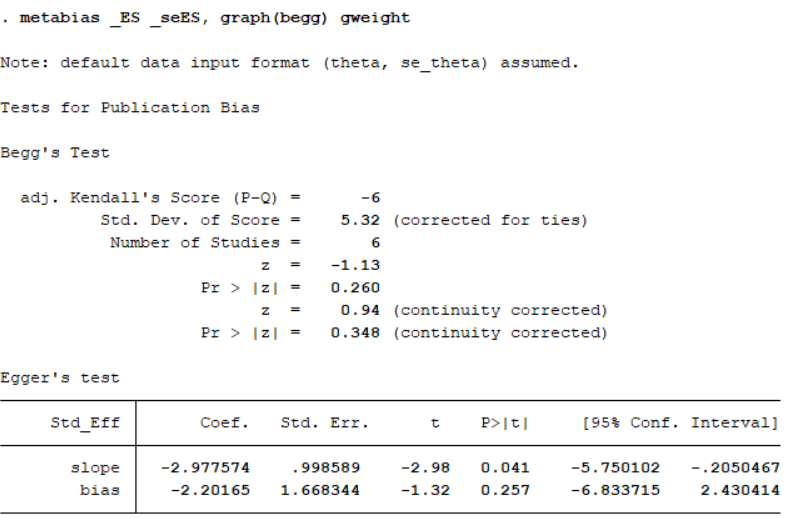


**The results of Eegger and Begger analysis of safety**


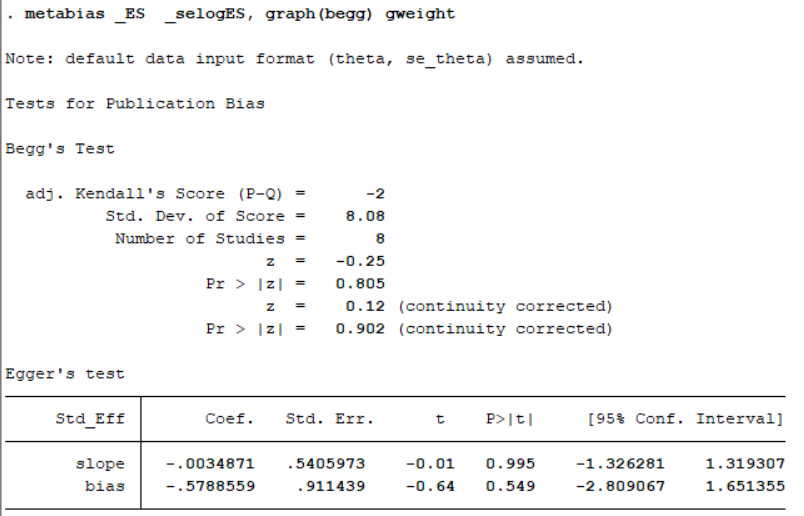


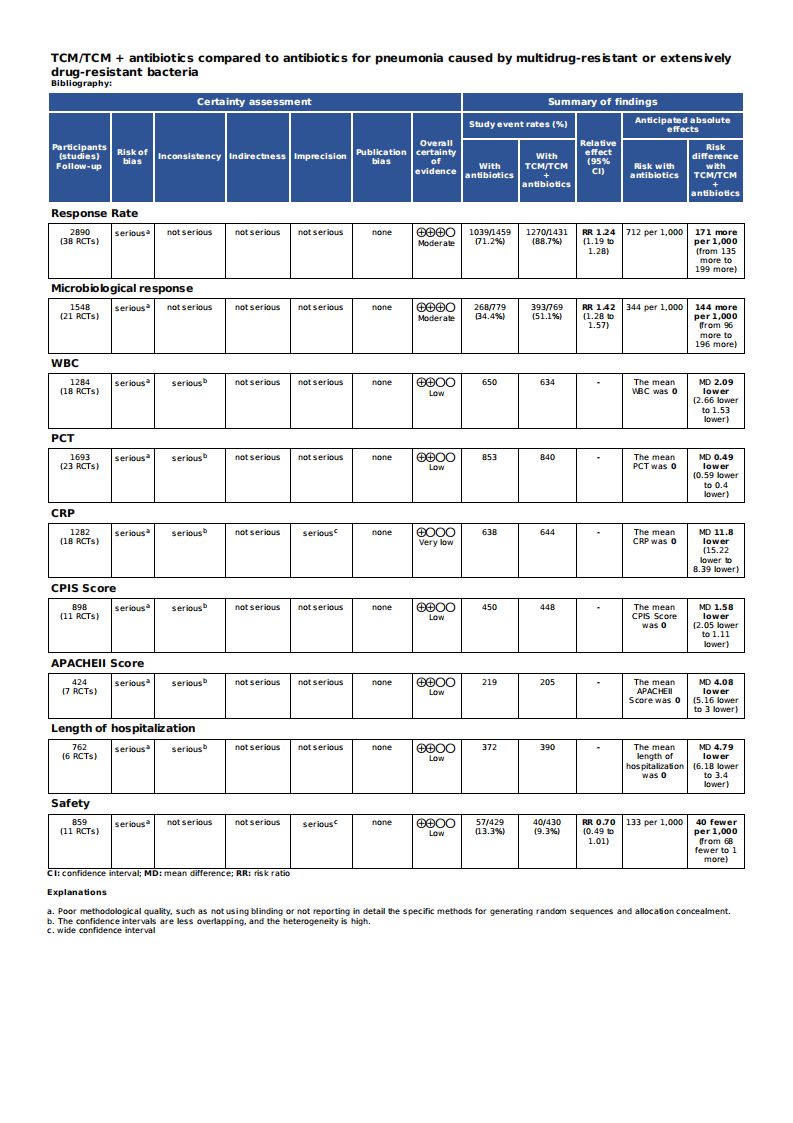
**Supplementary Material S8. Quality of Evidence According to Outcome Measures-GRADE**
